# High‐Resolution AFM Visualization of Nanocellulose Surface Grafting with Small Molecules

**DOI:** 10.1002/smll.202501435

**Published:** 2025-07-24

**Authors:** Lucas J. Andrew, Ayhan Yurtsever, Raksha Kandel, Seiya Ota, Keisuke Miyazawa, Takeshi Fukuma, Mark J. MacLachlan

**Affiliations:** ^1^ Department of Chemistry University of British Columbia 2036 Main Mall Vancouver British Columbia V6T 1Z1 Canada; ^2^ WPI Nano Life Science Institute Kanazawa University Kanazawa 920‐1192 Japan; ^3^ Stewart Blusson Quantum Matter Institute University of British Columbia 2355 East Mall Vancouver British Columbia V6T 1Z4 Canada; ^4^ UBC BioProducts Institute 2385 East Mall Vancouver British Columbia V6T 1Z4 Canada

**Keywords:** atomic force microscopy, characterization methods, functionalization, nanocellulose, sustainable materials

## Abstract

Nanocelluloses are attractive for bio‐based materials development due to their renewability, intrinsic properties, and ease of functionalization. However, characterization of nanocelluloses functionalized with small molecules at low grafting densities is challenging. Here, the use of high‐resolution frequency modulation atomic force microscopy (FM‐AFM) to directly visualize the covalent grafting of small molecules is demonstrated for the first time. Using epichlorohydrin as a linker, α‐cyclodextrin, β‐cyclodextrin, and tris(4‐*tert*‐butylphenyl)methanol are attached to the surface of bacterial nanocellulose. The presence of these groups is verified through spectroscopic measurements, and their covalent attachment is confirmed with FM‐AFM. Furthermore, the retention of cyclodextrin host‐guest activity after surface grafting is also demonstrated. This work represents a novel application of FM‐AFM for the characterization of small molecule‐grafted nanocelluloses, allowing for future development of advanced functionalization strategies.

## Graphical abstract

The covalent grafting of small molecules to the surface of nanocellulose is demonstrated for the first time using high‐resolution frequency modulation atomic force microscopy (FM‐AFM). The grafted molecules are distinctly visible and distinguishable from typical surface defects. Host‐guest activity of grafted cyclodextrins is also preserved after grafting, as shown by spectroscopic experiments. This work details a novel method for the historically difficult problem of characterizing surface‐grafted nanocelluloses.

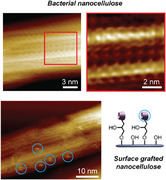

## 1 Introduction

Nanocelluloses are high aspect ratio crystalline nanoparticles produced from cellulose, an abundant component of biomass. With dimensions ranging from ≈100–300 nm in length and ≈5–20 nm in width, they are attractive for the design of next‐generation materials owing to their excellent mechanical properties, interesting self‐assembly behavior, and high tunability through surface modifications.^[^ ^1^, ^2^ ^]^ Nanocelluloses are also biosynthesized by some bacteria (termed bacterial nanocellulose, BC), resulting in increased crystallinity, higher aspect ratios, and higher chemical purity.^[^ ^3^ ^]^

Nanocellulose surfaces can be modified to greatly expand their functionality.^[^ ^2^ ^]^ For example, hydrophobic modifications can improve solubility in non‐aqueous solvents,^[^ ^4^, ^5^ ^]^ and grafting with cross‐linkable groups can improve the mechanical properties of nanocellulose‐based composites.^[^ ^6^, ^7^ ^]^ Applications have even expanded into biomedicine, enabled by the grafting of drug‐binding moieties^[^ ^8^ ^]^ or fluorophores.^[^ ^9^ ^]^

However, the characterization of functionalized nanocelluloses is very challenging. When small molecules or low‐density grafting strategies are employed, the grafted moieties constitute a small fraction of the material's overall composition. As a result, verifying functionalization and distinguishing between surface adsorption and covalent bonding can be difficult. Consequently, researchers often resort to indirect methods.^[^ ^10^, ^11^ ^]^ This challenge is further exacerbated when other bio‐based molecules, such as cyclodextrins, are grafted due to structural similarities to cellulose.^[^ ^12^ ^]^

Atomic force microscopy (AFM) is a useful technique for nanomaterial characterization, providing information on particle morphology, mechanical properties, and particle‐particle interactions.^[^ ^13^, ^14^, ^15^, ^16^, ^17^ ^]^ AFM can even resolve molecular‐scale detail on the surface of nanocelluloses and nanochitins.^[^ ^18^, ^19^ ^]^ Therefore, in this work, we applied high‐resolution frequency modulation AFM (FM‐AFM) to confirm the covalent grafting of small molecules on the surface of nanocellulose, acting as direct visual evidence of functionalization. We examined the grafting of α‐ and β‐cyclodextrin (α‐CD, β‐CD), as well as tris(4‐*tert*‐butylphenyl)methanol (supertrityl, ST) on the surface of BC. α‐CD and β‐CD were chosen due to their chemical similarity to BC and potential for future composite material development, and ST was chosen to assess this technique for analysis of grafted molecules with varying morphologies and hydrophilicities. The results demonstrate that FM‐AFM is a powerful technique for verifying the grafting of small molecules to the surface of nanocellulose.

## 2 Results and Discussion

### 2.1 Fabrication of Small Molecule‐Grafted Bacterial Nanocellulose

**Scheme 1:**
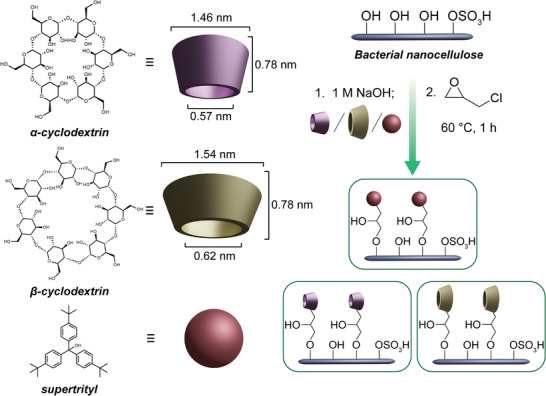
Structures of grafted molecules, and procedure for the surface grafting of BC using epichlorohydrin as a linker. Images are representations only and are not to scale.

**Figure 1:**
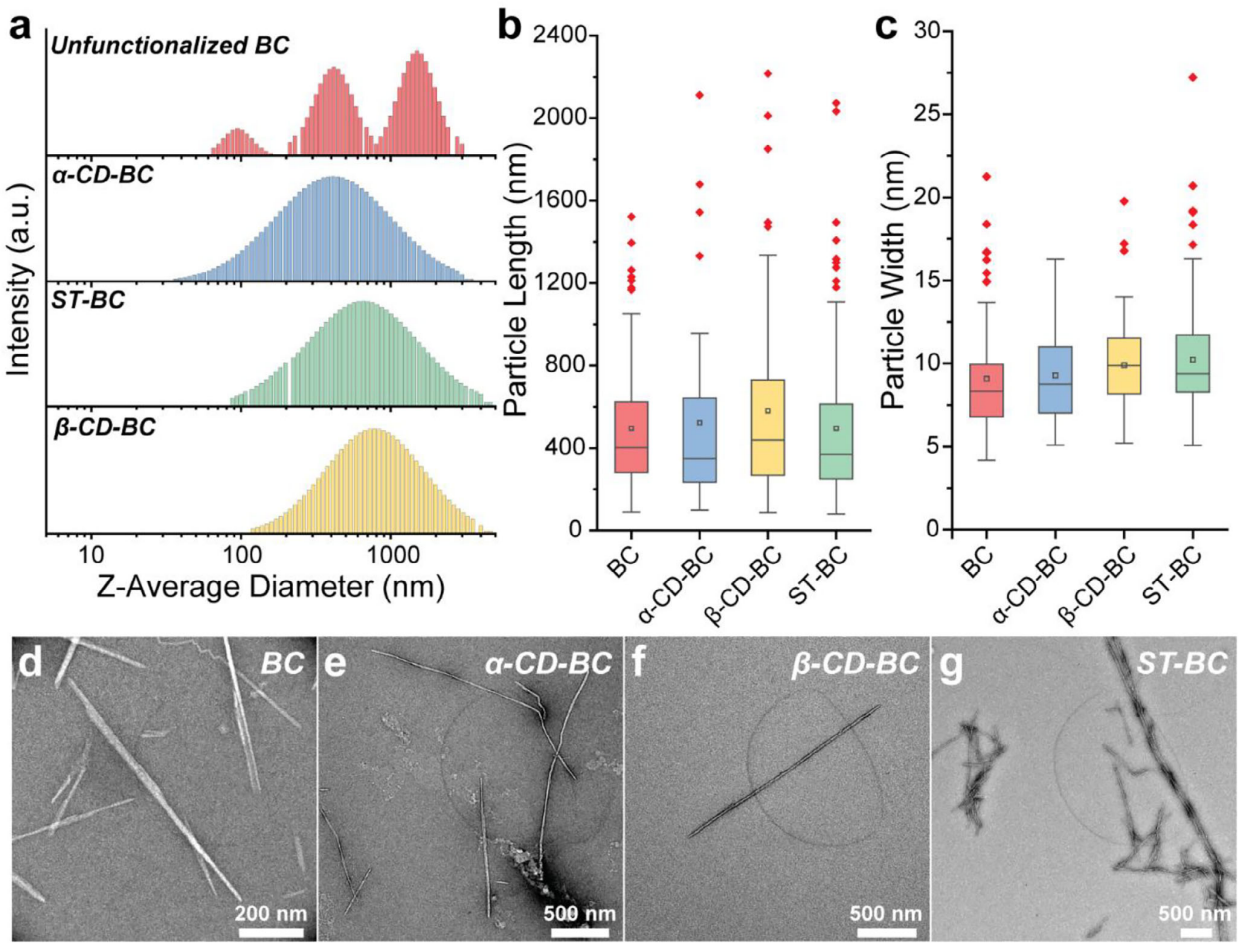
Characterization of unfunctionalized and grafted BC. a) Z‐average diameter size distributions of functionalized BCs as determined by DLS. CONTIN distributions are used for all samples except BC. Box plots illustrating b) length and c) width of functionalized BCs as determined by TEM. Boxes represent the Q1‐Q3 IQR, horizontal lines the median, squares the mean, and whiskers 0% and 100%. Outliers (red dots) are > 1.5 IQR. d‐g) Representative TEM micrographs of BC, α‐CD‐BC, β‐CD‐BC, and ST‐BC, respectively.

BC was isolated from coconut jellies (Figure S1, Supporting Information) through sulfuric acid hydrolysis,^[^ ^19^, ^20^ ^]^ affording partially sulfonated nanocrystals (S content = 191 mmol kg^−1^ by conductometric titration, Figure S2, Supporting Information). Grafting was performed using epichlorohydrin as a linker according to **Scheme** **1**. Transmission electron microscopy (TEM) and dynamic light scattering (DLS) revealed minimal changes in particle morphology after grafting (**Figure** **1**; Figure S3, Supporting Information). The ζ‐potentials of the grafted BC remained relatively constant at −27 ± 1 mV for unfunctionalized BC and −29 ± 1, −24 ±, and −26 ± 1 mV for α‐CD‐BC, β‐CD‐BC, and ST‐BC, respectively. Dimensional differences between samples were insignificant as determined by TEM (Supplementary Discussion 1).

### 2.2 Preliminary Characterization

Fourier transform infrared (FTIR) spectroscopy confirmed the presence of ST in ST‐BC, but results for α‐/β‐CD‐BC were inconclusive due to the chemical similarity between BC and cyclodextrins (Figure S4, Supporting Information). Therefore, we turned to UV–vis spectrophotometry. In the presence of α‐CD, methyl orange, a common azo dye and pH indicator, undergoes a hypsochromic shift in absorbance.^[^ ^21^ ^]^ Similarly, the pink color of phenolphthalein, a pH indicator, is attenuated in the presence of β‐CD.^[^ ^22^ ^]^ Taking advantage of these host‐guest interactions, we calculated the concentrations of grafted α‐CD and β‐CD as 7.2% and 10.3% by mass respectively for α‐CD‐BC and β‐CD‐BC (Figures S5, S6 and Supplementary Tables S1,S2, Supporting Information).

Solid‐state ^13^C Cross‐Polarization Magic Angle Spinning Nuclear Magnetic Resonance (CP/MAS NMR) experiments were also conducted to verify functionalization. This proved insufficient to confirm the attachment of α‐/β‐CD (Figures S7 and S8, Supporting Information), but the aromatic character of ST enabled its identification by NMR, showing clear evidence of ST grafting on ST‐BC (Figure S9, Supporting Information). Thermogravimetric analysis (TGA) also revealed slight differences in thermal degradation rates between grafted and unfunctionalized BC samples (Figure S10, Supporting Information). In all cases, the maximum rate of degradation (shown by peak height in the DTG traces) was lower for grafted samples, as the differential thermal stability of BC and CD resulted in a distribution of thermal degradation processes over a wider temperature range. Additionally, significant weight loss due to water evaporation was observed at low temperatures for neat CD, in contrast to BC‐containing samples. However, the differences in TGA traces were too minimal to make concrete conclusions.

### 2.3 High‐Resolution FM‐AFM Investigation of Bacterial Nanocellulose

**Figure 2:**
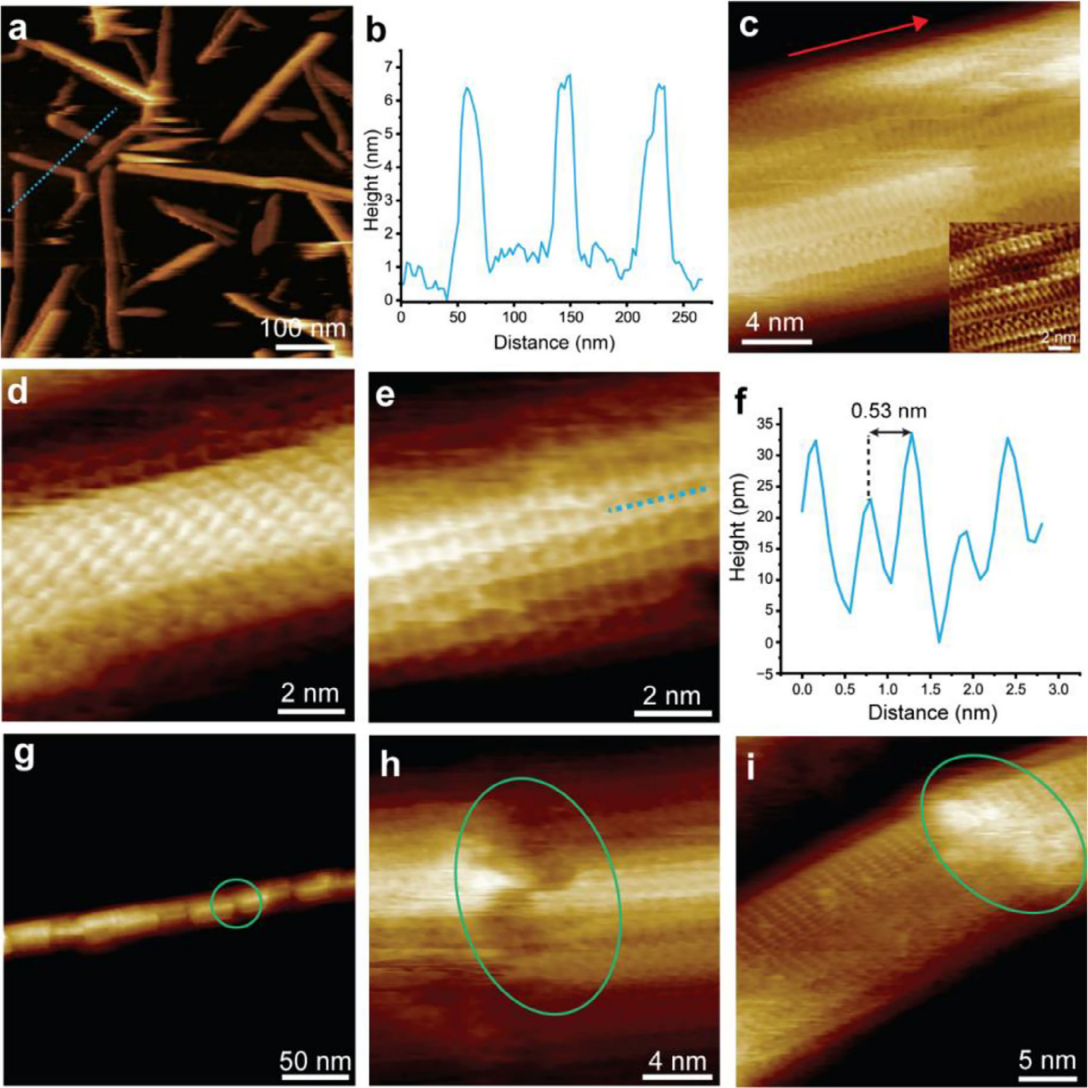
High‐resolution FM‐AFM images of unfunctionalized BC. a) Low magnification survey image. b) Height profile along the dotted blue line shown in a). c) High‐resolution FM‐AFM image acquired over a clean surface area, revealing the molecular arrangements of individual cellulose chains at the crystal surface. Inset in c) is an FFT‐reconstructed AFM image obtained by executing an inverse 2D FFT, which shows a well‐ordered chain organization. d‐e) Further high‐resolution images of clean BC surfaces, showing the structural organization of different crystalline planes. f) Height profile along the dotted blue line in e) illustrating the cellulose intermolecular spacing. g‐i) High‐resolution FM‐AFM images showing characteristic defects aligned perpendicularly across the BC nanocrystal surface (green circles). The red arrow shows the direction of the cellulose molecular axis.

Unable to verify the covalent attachment of α‐/β‐CD using conventional methods, we turned to FM‐AFM for further investigation. Low magnification images of unfunctionalized BC revealed similar morphologies as seen in TEM, with disperse lengths and relatively uniform widths (**Figure** **2**a,b). Notably, no significant difference in particle widths/diameters was observed between FM‐AFM and TEM measurements or when mounting AFM samples on HOPG substrate, suggesting that the AFM sample fixation process did not introduce any particle morphology bias (Figure S11, Supporting Information). Higher resolution images enabled visualization of the cellulose crystalline lattice (Figure 2c–e) and characteristic BC surface defects (Figure 2g–i; Figure S12, Supporting Information). Previous works have demonstrated the presence of similar defects through electron tomography^[^ ^23^ ^]^ and cryo‐SEM.^[^ ^24^ ^]^ Nanocellulose does not possess a natively smooth surface, and additional defects can also be introduced during processing. Such features can also arise as a result of loosely‐bound water or water adlayers on the particle surface, as shown in previous AFM studies.^[^ ^18^, ^19^ ^]^ Further guidance for identifying defects is provided later in Supplementary Discussion 2 (Figures S22–S25, Supporting Information).

**Figure 3:**
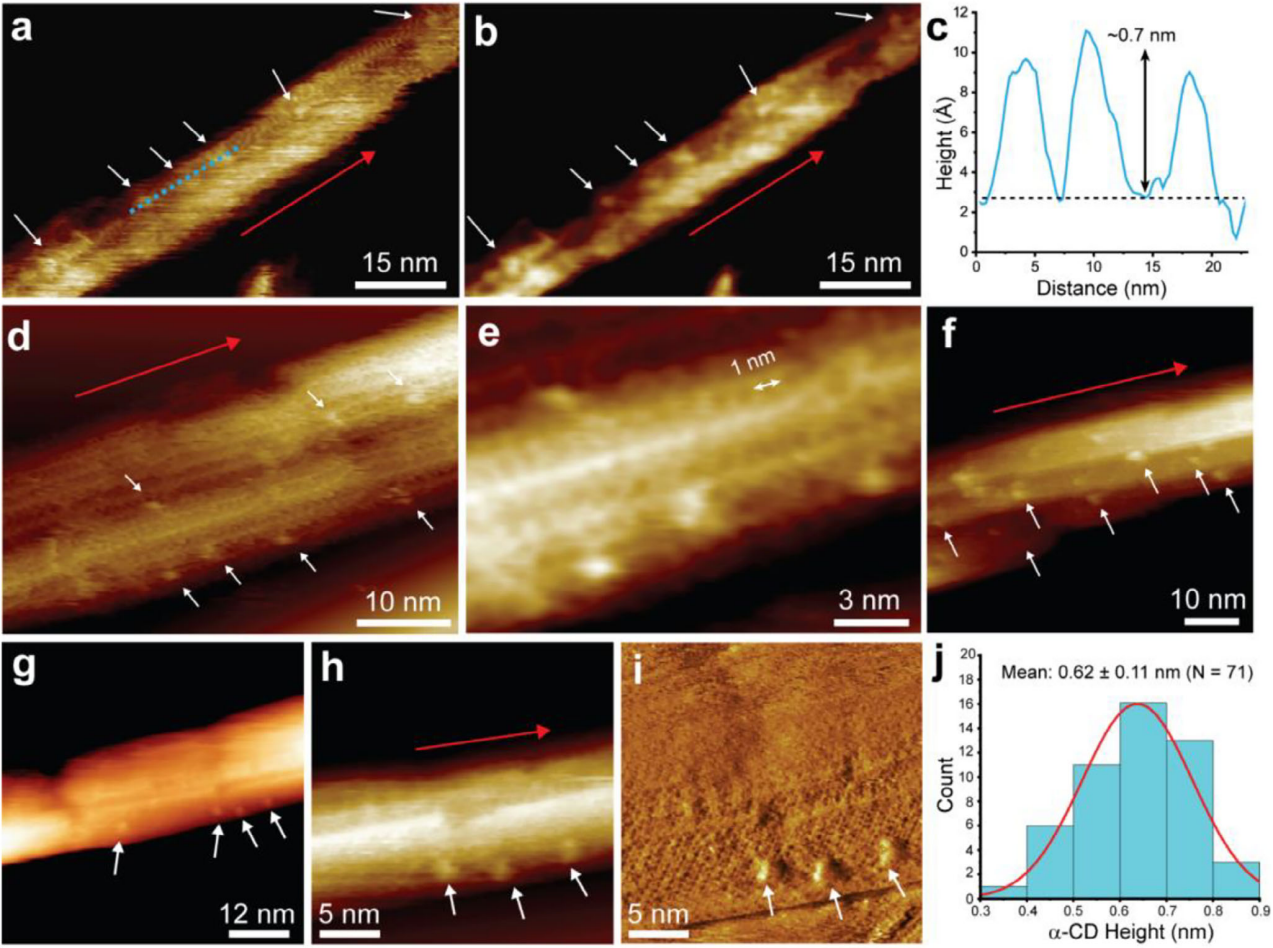
High‐resolution FM‐AFM images of α‐CD‐BC. a–b) Lower magnification image of a portion of a single α‐CD‐BC nanocrystal with grafted α‐CD. c) Height profile along the dotted blue line shown in (a). d–h) FM‐AFM images from a separate batch of α‐CD‐BC, illustrating smooth BC surfaces with α‐CD particles dispersed throughout. The image in (i) presents the dissipation map recorded simultaneously with the surface topology in (h). It shows that grafted α‐CD molecules are located on the crystal surface, while no particles are observed elsewhere, indicating the absence of contamination. j) Height distribution of grafted α‐CD particles. White arrows indicate grafted α‐CD; red arrows indicate the direction of the cellulose molecular lattice.

We then proceeded to image α‐CD‐BC, β‐CD‐BC, and ST‐BC. Low magnification images of α‐CD‐BC revealed a similar size distribution and morphology compared to unfunctionalized BC (**Figure** **3**a; Figure S13a,b, Supporting Information). Upon magnification, well‐defined protruding molecular features can be seen on the surface of the particles (Figure 3b–h; Figure S13c–h and S14, Supporting Information). These features are discrete and relatively non‐diffuse, suggesting covalent attachment (non‐covalently bound particles are expected to be significantly disturbed by the rastering AFM probe). These features have an average height of 0.62 ± 0.11 nm, matching well with α‐CD (Figure 3c,j). While it is possible in theory to also estimate grafting density using these data, the 3D structure of BC makes this much more difficult. Particles may bind to both sides of and beneath the nanocrystals. Most importantly, resolving grafted particles requires localized scanning of small surface areas, making it very time‐consuming to scan the entire surface of an individual BC fiber. Therefore, achieving molecular‐resolution imaging of all accessible surface areas – accounting for grafted particles attached to the CNC surface from the edges as well as below – makes it impractical to quantify the density of functionalization by AFM. Consequently, calculation of grafting density using complementary techniques is recommended instead. Functionalization also appears to not affect the crystallinity of BC, as the intact lattice structure can be clearly observed around grafting sites (Figure 3d‐i; Figure S13c,e–h and S14b–h, Supporting Information).

Control samples were also prepared to assess the effect of processing conditions on the BC surface. Samples prepared using identical processing conditions but excluding α‐CD showed only the presence of characteristic defects; no features reminiscent of grafted α‐CD were observed (Figure S15, Supporting Information). Samples prepared using identical processing conditions but excluding epichlorohydrin showed the same result (Figure S16, Supporting Information). A third set of control samples prepared by simple mixing of BC and α‐CD also revealed that non‐covalently bound CD molecules were not visible by FM‐AFM, reinforcing the capability of high‐resolution FM‐AFM for assessing covalent grafting (Figure S17, Supporting Information). Finally, we also employed extended resolution fluorescence microscopy using calcofluor white, a common dye for labeling plant cell walls, as a comparison to high‐resolution FM‐AFM. It was not possible to resolve grafted α‐CD in α‐CD‐BC samples by this technique (Figure S18, Supporting Information), due to a few key factors. First, calcofluor white does not label CD specifically, making identification of grafted features difficult. Furthermore, to truly resolve molecular features on the BC surface, a single molecule localization microscopy (SMLM) technique such as STORM would be more appropriate. However, this requires material design centered around this technique from the beginning. In contrast, high‐resolution FM‐AFM can be performed on the samples as‐is without any careful consideration during material design, making it a more versatile characterization technique for small molecule‐grafted nanocelluloses.

**Figure 4:**
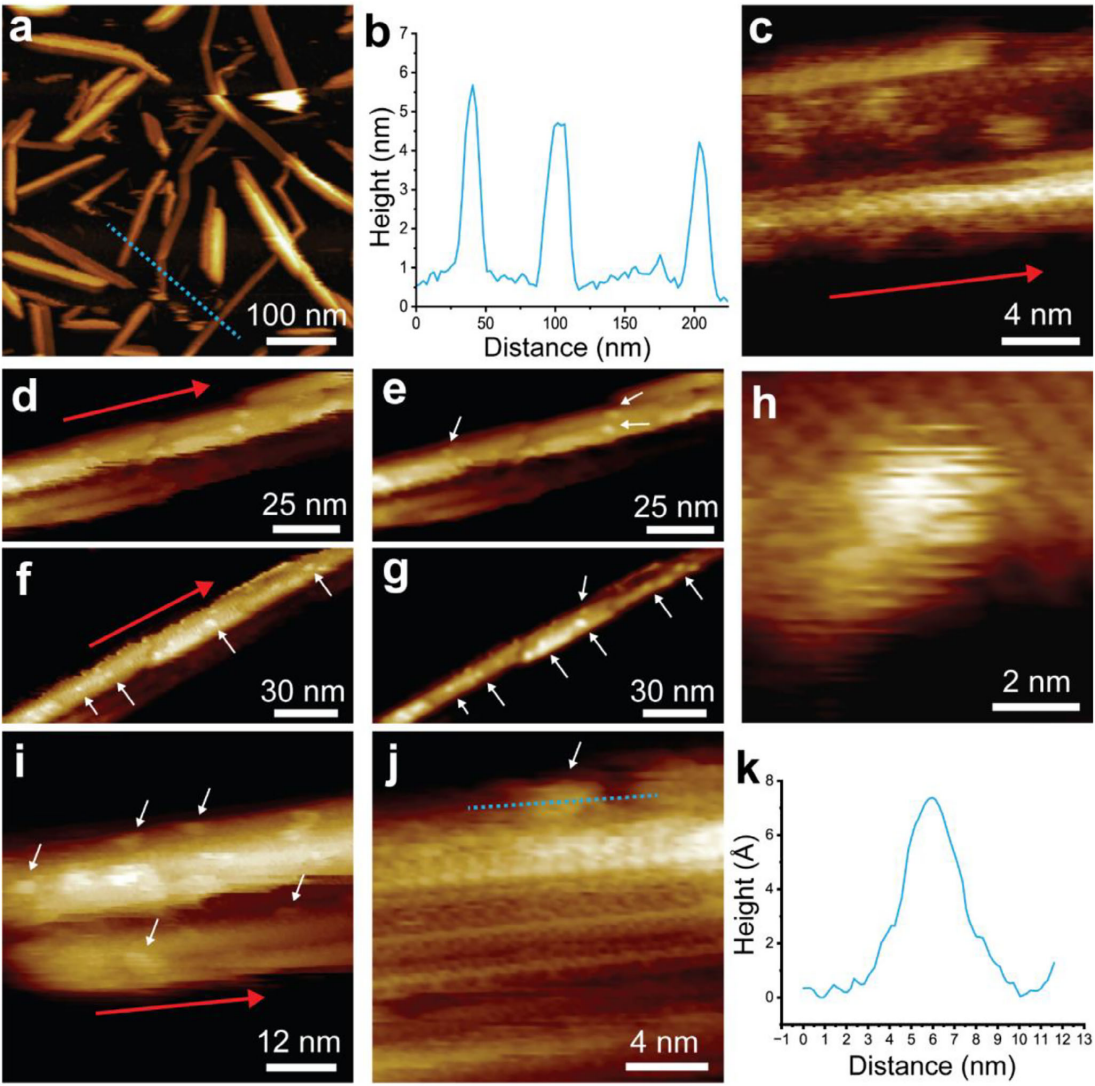
High‐resolution FM‐AFM images of β‐CD‐BC. a) Low magnification image of β‐CD‐BC nanocrystals and b) height profile along the dotted blue line. c) Higher magnification image of a portion of a nanocrystal, where grafted β‐CD can be seen along with diffuse patches indicating surface defects. d,f) Lower magnification images of two separate nanocrystals where β‐CD‐BC particles can be seen attached to the surface. e,g) Smoothed images of panels d) and f), respectively. h) Image of an individual grafted β‐CD‐BC particle. i‐j) Additional higher magnification images of β‐CD‐BC, illustrating some regions of higher β‐CD grafting density and k) height profile along the dotted blue line in j). White arrows indicate grafted particles; red arrows indicate the direction of the cellulose molecular axis.

Similar features were observed for β‐CD‐BC. Low magnification scans showed little change compared to unfunctionalized BC (**Figure** **4**a,b). At higher magnification, discrete circular features with a height comparable to β‐CD can be seen resting on top of the well‐defined cellulosic crystal lattice (Figure 4c–j; Figure S19, Supporting Information).

**Figure 5:**
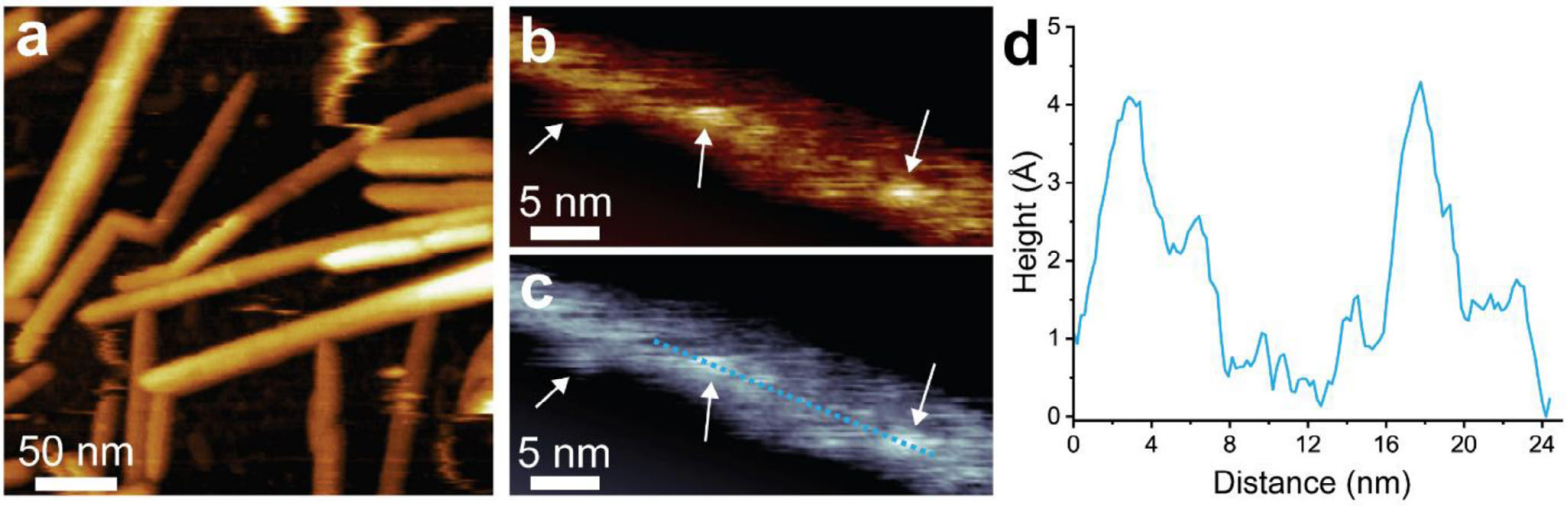
High‐resolution FM‐AFM images of ST‐BC. a) An AFM overview image of ST‐BC nanocrystals spread onto a PLL‐modified mica substrate in water, revealing the presence of well‐dispersed ST‐BC nanostructures. b‐c) Image of a single ST‐BC nanocrystal in two color schemes, showing grafted moieties along the surface (indicated by white arrows) attributed to ST. d) Height profile along the blue dotted line shown in c), illustrating a size of ≈4–5 Å for the grafted ST molecules.

FM‐AFM analysis of ST‐BC also revealed distinctive features on the BC surface (**Figure** **5**; Figure S20, Supporting Information). These features have a height of ≈4‐5 Å and a somewhat oblong shape, matching well with the optimized structure calculated by DFT (Figure S21 and Supplementary Table S3, Supporting Information). They appear more diffuse than α‐ and β‐CD but are still distinct from larger surface patches caused by defects or water adlayers. The visibility of these hydrophobic, more flexible, non‐macrocyclic molecules on the surface of nanocrystalline BC is a promising sign for the versatility of this technique.

Overall, high‐resolution FM‐AFM shows great promise for the confirmation of small molecule grafting on the surface of nanocellulose. However, care must be taken to ensure accurate differentiation of defects from grafted molecules. A step‐by‐step guide to the analysis of nanocellulose defects and grafted species is therefore given in Supplementary Discussion 2 (Figures S22–S25, Supporting Information). We expect this technique to be generalizable for the identification of a wide range of conformationally well‐defined species, such as macrocycles, rigid small molecules, and inorganic nanoparticles/quantum dots. It may be limited in its ability to identify more flexible species, such as shorter linear hydrocarbons or low MW polymers. Even so, the identification of larger species is easily performed with lower resolution AFM and/or complementary techniques. As a result, we believe that high‐resolution FM‐AFM has great potential for small molecule‐functionalized materials.

### 2.4 Host‐Guest Chemistry of Grafted Cyclodextrins

**Figure 6:**
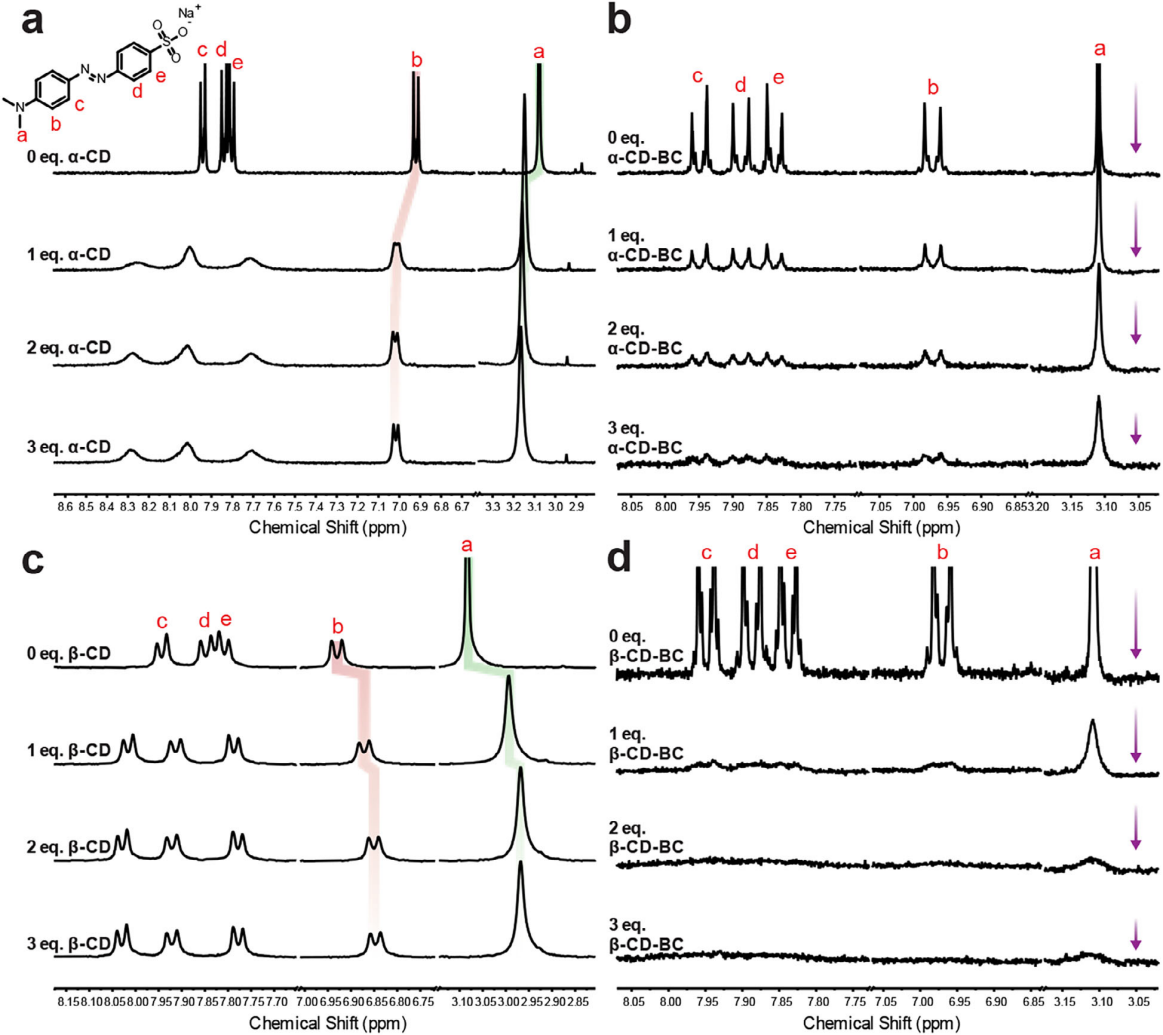
Host‐guest chemistry of grafted α‐/β‐CD. Partial ^1^H NMR spectra (400 MHz, D_2_O) of a) methyl orange (2 mm) and α‐CD, b) methyl orange (59 µm) and α‐CD‐BC, c) methyl orange (2 mm) and β‐CD, and d) methyl orange (90 µm) and β‐CD‐BC. Only peaks corresponding to methyl orange are shown; all peaks are labeled according to the scheme in panel a).

Having successfully imaged the grafted α‐CD, β‐CD, and ST, we endeavored to further demonstrate grafting of α‐ and β‐CD by probing their host‐guest chemistry while attached to the BC surface. Using ^1^H NMR spectroscopy, we examined changes in the signals for methyl orange upon the addition of free α‐/β‐CD, α‐/β‐CD‐BC, and free BC as a control. In the case of free α‐/β‐CD, the methyl orange signals undergo an easily observable change. For α‐CD, peak broadening and shifting are observed upon host‐guest complexation, and for β‐CD, broadening is not observed but peak positions clearly change (**Figure** **6**a,c, full spectra in Figures S29 and S30, Supporting Information). In contrast, the addition of α‐/β‐CD‐BC results in attenuation of methyl orange peaks without a prominent change in chemical shifts (Figure 6b,d, full spectra in Figures S31 and S32, Supporting Information). This effect is attributed to the transfer of methyl orange from solution to suspension upon complexation with grafted CD, rendering it undetectable by ^1^H NMR. Attenuation was also observed in BC‐only controls (Figures S33–S36, Supporting Information) due to adsorption of methyl orange onto the nanocrystal surface. However, this effect is much less pronounced and cannot account for the dramatic attenuation observed upon α‐/β‐CD‐BC addition. Control experiments employing both BC and free α/β‐CD showed comparable behavior to free α/β‐CD experiments, demonstrating the disparate binding behavior of free and grafted α‐/β‐CD (Figures S37–S40, Supporting Information).

The host‐guest chemistry of grafted α‐/β‐CD was also probed by fluorescence spectroscopy. Previous work has demonstrated host‐guest chemistry with nanocellulose‐grafted cyclodextrins using guest‐linked fluorescent tags,^[^ ^25^ ^]^ but here we examined the change in fluorescence of the guest itself, again using methyl orange. For α‐CD‐BC, a general increase in methyl orange fluorescence intensity was observed with increasing α‐CD concentration, in line with previous reports (Figure S43, Supporting Information).^[^ ^26^, ^27^ ^]^ Fluorescence intensity decreased again at higher α‐CD‐BC concentration, however, possibly due to reduced excitation of the bound methyl orange. For β‐CD‐BC, fluorescence intensity initially dropped sharply before returning to its original level (Figure S45, Supporting Information). We postulate that the different behavior observed for α‐/β‐CD‐BC is due to the higher binding constant of β‐CD (≈2 x K_α‐CD_).^[^ ^28^ ^]^ Addition of higher equivalents of α‐CD‐BC may have resulted in the same effect, but limitations of grafted α‐CD concentration prevented further investigation.

## 3 Conclusions

In conclusion, we have shown that grafted α‐CD, β‐CD, and ST can be visualized on the surface of BC nanocrystals by high‐resolution FM‐AFM, suggesting covalent grafting. The grafted molecules are easily distinguished from common surface defects, allowing for reliable identification. Control experiments ruled out the possibility of grafted molecule‐like features arising from processing. Furthermore, simple mixing of BC and α‐CD revealed that adsorbed molecules cannot be observed by FM‐AFM, further confirming covalent grafting. Finally, we have demonstrated the host‐guest chemistry of BC‐grafted CD, opening the door for advanced composite materials development. We expect that these results will inspire researchers to delve further into the development of small molecule‐functionalized nanocelluloses, using high‐resolution FM‐AFM as a reliable direct characterization method.

## 4 Experimental Section

### Materials

Epichlorohydrin was purchased from Sigma–Aldrich, NaOH from Fisher, α‐cyclodextrin hydrate from Oakwood Chemical, and β‐cyclodextrin hydrate from Acros Organics. All chemicals were used as received without further purification. Coconut jellies (nata de coco) used to fabricate bacterial cellulose were purchased from a local grocery store (Pearl Delight Brand, Philippines).

### Isolation of Bacterial Nanocellulose (BC)

1 kg of coconut jellies was drained and washed thoroughly under running water to remove syrup, followed by a final wash with Milli‐Q water. The washed jellies were blended to a homogenous pulp, and 200 g were added to a 2 L round bottom flask filled with 1 L of 1 wt.% NaOH solution. The mixture was heated at reflux for 2 h, quenched with ice cold water, then vacuum filtered, followed by 5 x 200 mL washes with Milli‐Q water and 3 x 200 mL washes with 0.2 wt.% AcOH. The vacuum was left on until the pulp formed a dry cake. This initial NaOH purification step was repeated 5x in total until all 1 kg of coconut jellies were used.

The NaOH‐purified pulp was then resuspended in 2.5 L of distilled water, and 500 mL was added at a time to a 2 L round bottom flask. 500 mL of 65% H_2_SO_4_ was added, and the mixture was heated at 50 °C with overhead stirring for 1 h. The reaction was quenched with ice cold water, and the resulting suspension was centrifuged and washed several times with Milli‐Q water to retrieve the bacterial nanocellulose. The hydrolysis procedure was performed 5x in total, until all 2.5 L of the pulp was used. The nanocellulose was then dialyzed against Milli‐Q water until the pH and conductivity of the dialysis bath remained constant (pH 7, ≈0 µS cm^−1^) for 3 consecutive days. The resulting suspension was ultrasonicated to ensure adequate dispersion and concentrated to 12 mg mL^−1^ by evaporation under ambient room conditions.

### Synthesis of Tris(4‐*tert*‐butylphenyl)methanol (Supertrityl, ST)

Synthesis of ST was carried out based on a previous literature report.^[^ ^29^ ^]^ To a stirred suspension of magnesium (673 mg, 25.9 mmol, washed with 1 M HCl and MeOH before use) in dry THF (20 mL) was added dropwise (15 min) a solution of 1‐bromo‐4‐tert‐butylbenzene (4.5 mL, 26 mmol) in dry THF (20 mL). The mixture was then vigorously heated to initiate the reaction and was then stirred at room temperature for 1 h. A solution of methyl 4‐tert‐butylbenzoate (2 mL, 10.4 mmol) in dry THF (20 mL) was added dropwise over 20 min, and the reaction was stirred at room temperature overnight, after which time a saturated aqueous solution of NH_4_Cl (200 mL) was added. The reaction mixture was next extracted with CH_2_Cl_2_ (4 x 50 mL). The combined organic layers were dried over Na_2_SO_4_ and dried by rotary evaporation. The crude product was purified by washing with hexane (3 x 50 mL) to give tris(4‐tert‐butylphenyl)methanol as a white powder (3.17 g, 7.38 mmol, 71%). ^1^H NMR data were in agreement with literature values. ^1^H NMR (CDCl_3_, 400 MHz): δ = 1.31 (s, 27H), 7.19 (*dt*, J = 2.1, 8.7 Hz, 6H), 7.32 (*dt*, J = 2.4, 8.4 Hz, 6H). The OH peak was not observed due to broadening.

### Grafting of Bacterial Nanocellulose

3.5 mmol of α‐cyclodextrin (α‐CD) or β‐cyclodextrin (β‐CD) were added to 15 mL of 2.67 m aqueous NaOH and stirred until dissolved in a 100 mL round bottom flask. 25 mL of 12 mg mL^−1^ bacterial nanocellulose (BC) suspension was then added (0.3 g dry mass equivalent), reaching a final volume of 40 mL and NaOH concentration of 1 M. The mixture was brought to 60 °C and stirred for 30 min with an overhead mechanical stirrer to equilibrate, after which 31 mmol (2.43 mL, 2.87 g) of epichlorohydrin was added dropwise over ≈2 min. The mixture was allowed to stir further for 1 h, at which point it was quenched with ice cold water, then centrifuged (5000 rpm, 10 min) and washed repeatedly with distilled water until the pH was neutral. The gel‐like product was resuspended in Milli‐Q water, then dialyzed against Milli‐Q water for 1 week (changing the dialysis bath water once per day). Finally, the suspension was ultrasonicated to ensure adequate dispersion, filtered, and concentrated to 4.8 mg mL^−1^ (α‐CD‐BC) or 6.1 mg mL^−1^ (β‐CD‐BC) by evaporation under ambient room conditions.

For ST‐grafted BC, the same ratio of reagents and procedure was used, but a different purification procedure was carried out. After 3x centrifugation wash cycles, the ST‐BC was resuspended in Milli‐Q water and added to a separatory funnel, followed by 3x washes with dichloromethane (DCM) to remove free ST. The aqueous layer was then filtered and rotovapped to remove residual DCM, followed by dialysis against Milli‐Q water for 1 week (changing the dialysis bath water once per day). Finally, the suspension was ultrasonicated to ensure adequate dispersion and concentrated to 4.2 mg mL^−1^ by evaporation under ambient room conditions. The DCM wash fractions were rotovapped to retrieve free ST and provide an estimate of the grafting efficiency – this value was calculated as ∼18%, but we note that this may be an overestimate due to free ST lost during recovery.

### Characterization—Conductometric Titration

Strong acidic cation exchange resin (Amberlite® IRC120 H, hydrogen form) was washed with EtOH until the rinse was colorless, then dried overnight at 60 °C. Then, it was added to 5 mL of BC suspension at a ratio of 0.25 g resin per g of BC (dry basis), and allowed to sit for 72 h. The resin was filtered off, and the BC suspension was diluted to 5 mg mL^−1^ with Milli‐Q water. The suspension was then titrated against a standardized aqueous solution of NaOH (1.724 mm) to determine the S content, in line with previous literature reports.^[^ ^30^ ^]^

### Characterization—Transmission Electron Microscopy (TEM)

TEM images were captured using a Tecnai G20 electron microscope (FEI) operating at 200 kV. Samples were diluted to 0.05 mg mL^−1^ and stained with 2% uranyl acetate, then deposited onto 400‐mesh Formvar‐coated Cu TEM grids before imaging. Particle dimensions were determined from the micrographs using ImageJ.

### Characterization—Dynamic Light Scattering (DLS)/ζ‐Potential

DLS and ζ‐potential measurements were collected using a NanoBrook Omni particle size analyzer (Brookhaven Instruments). Samples were diluted to 1 mg mL^−1^ (BC) or 0.5 mg mL^−1^ (α‐CD‐BC, β‐CD‐BC, ST‐BC) with MilliQ water before measurement. DLS measurements were run in triplicate, with each measurement consisting of 3 x 5 min measurement intervals. ζ‐Potential measurements were run in triplicate, with 30 scans per measurement. Reported values are expressed as averages ± standard deviations.

### Characterization—Fourier Transform Infrared (FTIR) Spectroscopy

FTIR spectra were collected using a Frontier FTIR (Perkin Elmer) equipped with an attenuated total reflection (ATR) detector (ZnSe crystal). Samples were lyophilized before measurement, and spectra were captured from 4000 to 650 cm^−1^ at a resolution of 4 cm^−1^, with a total of 16 scans per sample.

### Characterization—UV–Vis Spectrophotometry

UV‐vis data were collected using a Cary 5000 UV–vis spectrophotometer (Agilent) using a wavelength range of 250–900 nm, resolution of 1.5 nm, and a scan rate of 600 nm min^−1^.

### Characterization—Fluorescence Spectroscopy

Fluorescence measurements were carried out using a Cary Eclipse fluorescence spectrophotometer (Varian). Emission from methyl orange‐containing samples in the range of 350–550 nm was measured using a λ_ex_ of 310 nm and 20 nm excitation/emission slits, at a scan rate of 300 nm min^−1^ (0.5 nm resolution).

### Characterization—Atomic Force Microscopy (AFM)

Experiments were carried out in the liquid state using a custom‐built frequency modulation atomic force microscope (FM‐AFM) equipped with a low‐noise cantilever deflection sensor.^[^ ^31^ ^]^ The homemade AFM scanning process was controlled by a commercially available SPM controller (ARC2, Asylum Research), and an infrared laser beam with a wavelength of 785 nm was used to drive the oscillation of the cantilever. To maintain a constant amplitude of cantilever oscillation, a commercially available controller (OC4, SPECS) was used to adjust the amplitude of the excitation signal. Measurements were conducted in a constant frequency shift (*df*) mode, in which the distance between the tip and sample was regulated to keep *df* constant. AFM image data were acquired using a commercially available silicon cantilever (NCHAuD) with a nominal spring constant of 42 N m^−1^. The nominal radius of the Si tip apex was ≈7 nm. Cantilever spring constants were calibrated using the thermal noise method after each experiment.

For each measurement, an aqueous suspension of BC (grafted or non‐grafted) was first diluted to a concentration of 0.05 mg mL^−1^. The suspension was then sonicated using an ultrasonic homogenizer (MITSUI, 3 mm diameter tip, maximum power of 500 W). The aqueous suspensions of the cellulose samples were placed in an ice bath to prevent excessive heating, and sonicated for 5 x 3 min. The BC suspensions were prepared for imaging by placing a drop of diluted sample (120 µL) onto a freshly cleaved mica substrate. The mica substrate was coated with 50 µL of 0.01% poly‐L‐lysine (PLL) solution (Merck) for ≈10 min, then rinsed several times with 100 µL of Milli‐Q water. In some cases, the mica substrate was exposed to APTES vapors in a vacuum desiccator for 20 min to effectively reverse the surface charge of the mica. We have also used HOPG without any functionalization as a substrate for the characterization of grafted BC. After incubation with the sample suspension for 25 min, the mica substrate was rinsed with 100 µL of Milli‐Q water several times to further reduce the concentration of BC and remove unbound particles, followed by imaging. Raw AFM data were processed by flattening and plane fitting to eliminate background tilt where necessary. To achieve optimal contrast in the AFM images, scanning parameters were also varied such as scanning angle, imaging set point, and scan rate.

A large number of AFM images were captured to ensure the uniformity of grafted features. In addition to lower resolution images, higher resolution images were taken along the length of BC fibers. In this case, multiple images were required to capture the entire length of a fiber. For example, a total of 837 images were taken for α‐CD‐BC, resulting in a total of 80 individual fibers imaged at high resolution. From these images were extracted height profile data for grafted α‐CD particles. Similarly, a total of 70 individual fibers were imaged at high resolution for β‐CD‐BC, and 49 were imaged for ST‐BC. Representative high‐resolution images were selected from these datasets for inclusion in the manuscript and Supporting Information.

### Characterization—Extended‐Resolution Fluorescence Microscopy

Imaging was performed using an Olympus/Evident IXplore SpinSR system (Evident, Tokyo, Japan), equipped with an inverted microscope (IX83; Evident, Japan), a CSUW1‐SoRa spinning disk confocal unit ((Yokogawa, Tokyo, Japan) and a Hamamatsu ORCA Fusion BT camera (Hamamatsu Photonic K.K., Tokyo, Japan). A 60x oil objective (UPLAPO 60xOHR/1.50 NA) with a 3.2x magnification changer was used to yield a total magnification of 192x. Fluorescent signals from calcofluor white‐stained cellulose were detected using 405 nm excitation and a 447/60 nm emission filter.

For extended‐resolution imaging, Z‐stacks were acquired with a step size of 200 nm or 500 nm in super‐resolution mode using the SoRa disk. Deconvolution was performed using a constrained iterative method with the following default settings: Modality, Laser Scanning Confocal or Spinning Disk Confocal and/or OSR; Algorithm: Advanced Maximum Likelihood; Iterations, 5. Images acquisition and processing were performed using were exported using cellSens Dimension software (Evident, Tokyo, Japan).

For images collected with the Leica Stellaris 8, the observation method was:

A Leica Stellaris 8 Confocal Scanning Laser Microscope (CSLM) from Leica Microsystems (Germany), operating in LIGHTNING Deconvolution mode, was used to acquire high‐resolution images. Fluorescence was excited using a 405 nm diode laser, and emission was detected between 425 and 480 nm. All CSLM images were collected with a HC PL APO 63x/1.40 Oil CS2 objective lens, and the pinhole was set to 1.00 AU. Maximum resolution in these images was achieved through LAS X Lightning adaptive deconvolution.

### Characterization—Nuclear Magnetic Resonance (NMR) Spectroscopy

^1^H NMR spectra were recorded on Bruker AV III HD 400 MHz and Bruker Avance 400 MHz spectrometers. Chemical shifts (δ) are reported in parts per million (ppm) and referenced to the residual solvent signal. Except for controls without BC: 800 scans were collected for each measurement, keeping the total volume and water volume constant, and the same solvent suppression power was applied for each sample and relevant controls.

CP/MAS ^13^C NMR measurements were performed at room temperature on a 400 MHz Bruker solid state widebore AVANCE spectrometer with 4 mm rotors. The operating frequencies of ^1^H and ^13^C are 400.13 and 100.62 MHz, respectively. The conventional CP/MAS method was used for high‐resolution solid‐state ^13^C NMR measurements. The rotors containing lyophilized nanocellulose samples were spun at ≈6 kHz, and the 90° pulse, contact time, and repetition time were 4.4 µs, 5 ms, and 5s, respectively. ≈32000 scans were collected for each measurement. Spectra were calibrated using the ^13^C resonance of adamantane C‐H at 37.78 ppm.

## Conflict of Interest

The authors declare no conflict of interest.

## Author Contributions

L.J.A. and A.Y. contributed equally to this work. L.J.A. was responsible for conceptualization, methodology, investigation, writing the original draft, reviewing and editing the manuscript, and visualization. A.Y. contributed to methodology, investigation, writing, review, and editing, and visualization. R.K. contributed to methodology, investigation, writing, review, and editing. S.O. was involved in the investigation. K.M. was involved in the investigation. T.F. handled project administration and funding acquisition. M.J.M. contributed through supervision, project administration, funding acquisition, and writing, review, and editing.

## Supporting information



Supporting Information

## Data Availability

The data that support the findings of this study are available in the supplementary material of this article.
