# Supplementary material for: High‐Resolution AFM Visualization of Nanocellulose Surface Grafting with Small Molecules
Source: Small. 2025 Jul 24;21(36):e01435. doi: 10.1002/smll.202501435 (PMC12423920; doi:10.1002/smll.202501435)
Supplement: Supplementary file 1 — Supporting Information [file SMLL-21-e01435-s001.pdf]

NANO · MICRO
small

Supporting Information

for *Small*, DOI 10.1002/smll.202501435

High-Resolution AFM Visualization of Nanocellulose Surface Grafting with Small Molecules

Lucas J. Andrew, Ayhan Yurtsever, Raksha Kandel, Seiya Ota, Keisuke Miyazawa, Takeshi Fukuma and Mark J. MacLachlan**

Supporting Information

High-resolution AFM visualization of nanocellulose surface grafting with small molecules

Lucas J. Andrew[a†], Ayhan Yurtsever[b†], Raksha Kandel[a], Seiya Ota[a], Keisuke Miyazawa[b], Takeshi Fukuma[b*], Mark J. MacLachlan[a,b,c,d*]

†LJA and AY contributed equally to this work.

AUTHOR ADDRESS

[a] Department of Chemistry, University of British Columbia, 2036 Main Mall, Vancouver, British Columbia, V6T 1Z1, Canada

[b] WPI Nano Life Science Institute, Kanazawa University, Kanazawa, 920-1192, Japan

[c] Stewart Blusson Quantum Matter Institute, University of British Columbia, 2355 East Mall, Vancouver, British Columbia, V6T 1Z4, Canada

[d] UBC BioProducts Institute, 2385 East Mall, Vancouver, British Columbia, V6T 1Z4, Canada

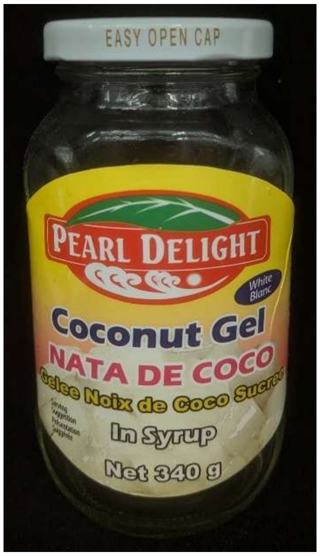

Figure S1. Coconut jellies used as the source of bacterial cellulose in this work.

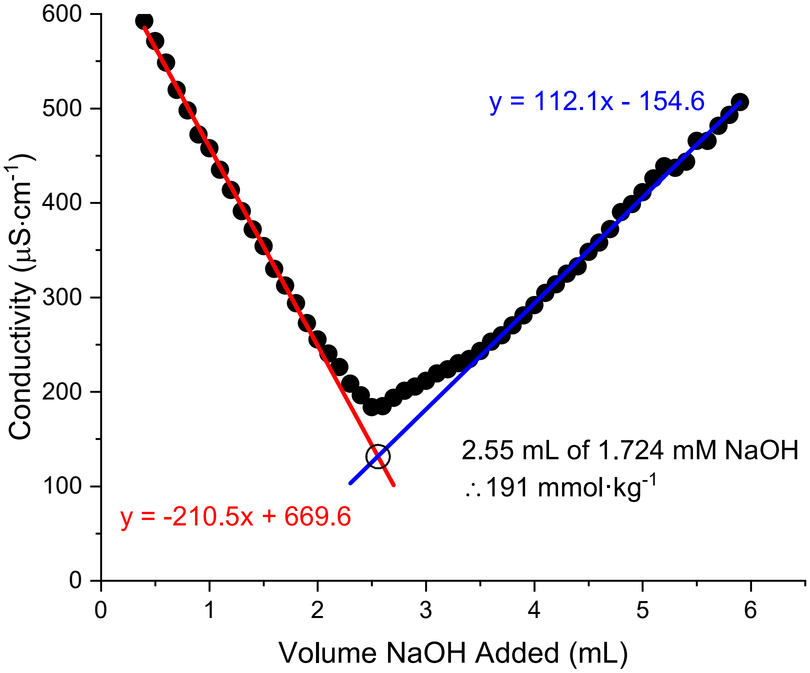

Figure S2. Conductometric titration of nanocrystalline BC for determination of S content.

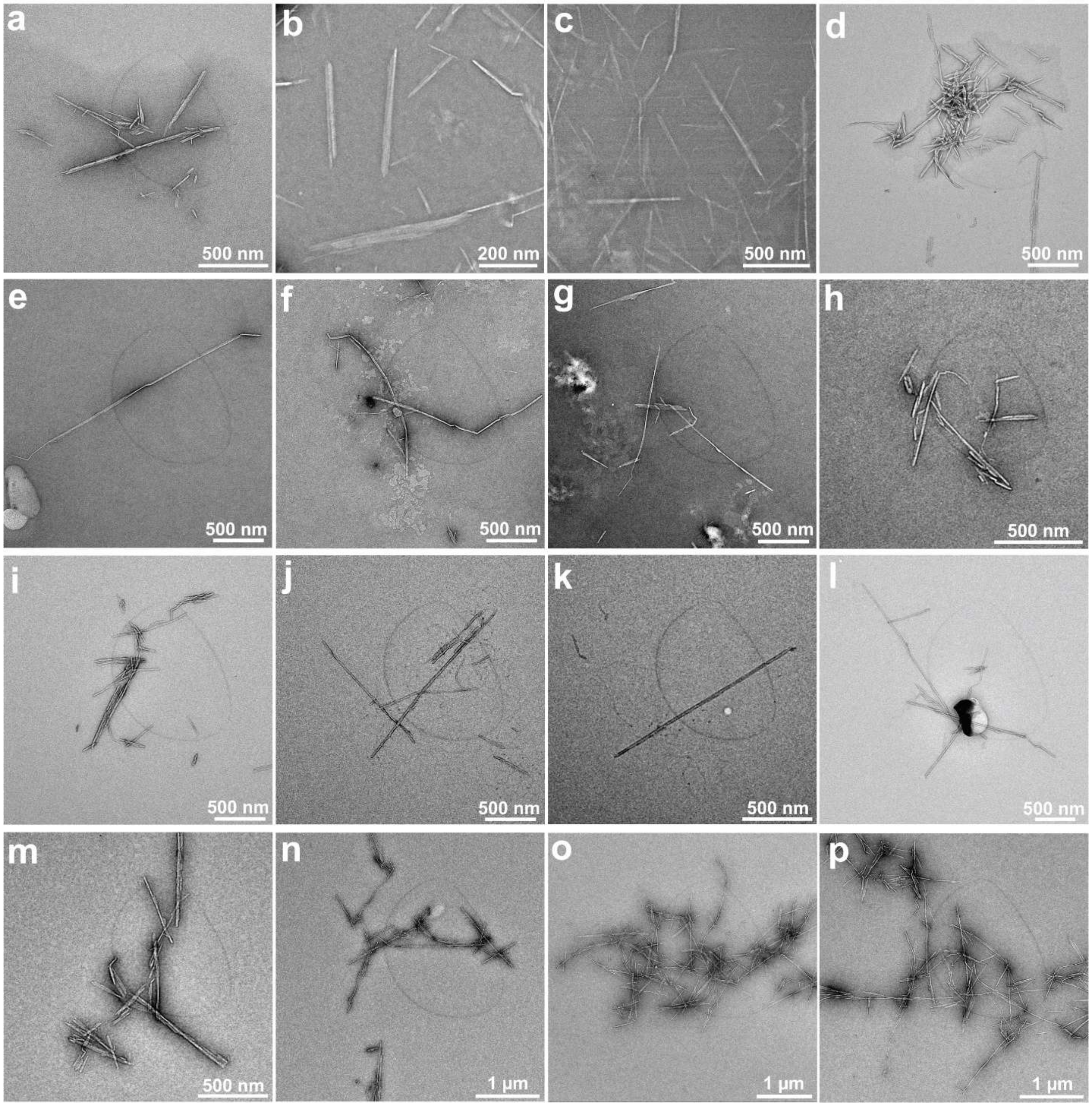

Figure S3. Supplemental TEM micrographs of a-d) BC, e-h) α-CD-BC, i-l) β-CD-BC, and m-p) ST-BC.

SUPPLEMENTARY DISCUSSION 1

Single-factor ANOVA analysis was used to assess the statistical similarity of grafted BC dimensions as determined by TEM. Analysis was carried out using Microsoft Excel (Microsoft 365) on full datasets of length and width for each set of samples, and the output is shown below:

PARTICLE LENGTH

SUMMARY

Groups	*Count*	*Sum*	*Average*	*Variance*
BC	108	53460.48	495.0044	99677.25
α-CD-BC	70	38129.17	544.7024	176284.4
β-CD-BC	66	38347.11	581.0168	210146.5
ST-BC	105	51985.27	495.0978	152058.7

ANOVA

Source of Variation	*SS*	*df*	*MS*	*F*	*P-value*	*F crit*
Between Groups	420464.4	3	140154.8	0.924491	0.429064	2.630792
Within Groups	52302725	345	151602.1			
Total	52723189	348				

PARTICLE WIDTH

SUMMARY

Groups	*Count*	*Sum*	*Average*	*Variance*
BC	102	926.216	9.080549	10.27521
α-CD-BC	77	809.083	10.50757	122.8095
β-CD-BC	82	811.622	9.897829	6.81947
ST-BC	133	1361.273	10.23514	10.58488

ANOVA

Source of Variation	*SS*	*df*	*MS*	*F*	*P-value*	*F crit*
Between Groups	111.709	3	37.23634	1.178661	0.317591	2.627789
Within Groups	12320.9	390	31.59205			
Total	12432.61	393				

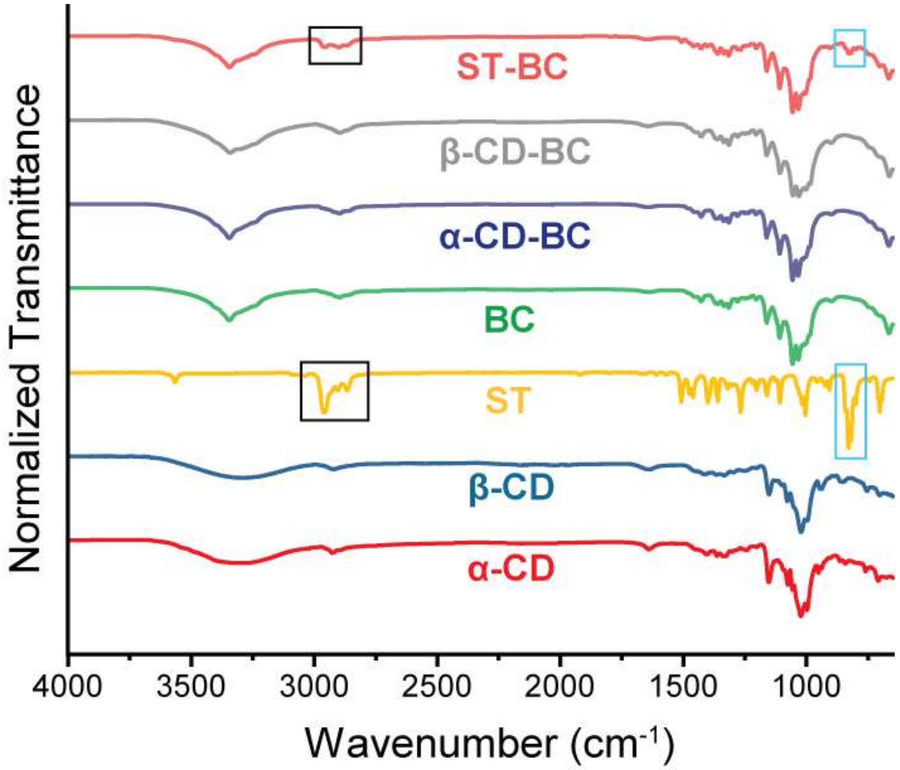

Figure S4. FTIR spectra of molecular grafts, unfunctionalized BC, and functionalized BCs. Regions highlighted with a box show spectral similarities between free ST and ST-BC, suggesting the presence of ST grafted on the surface of ST-BC.

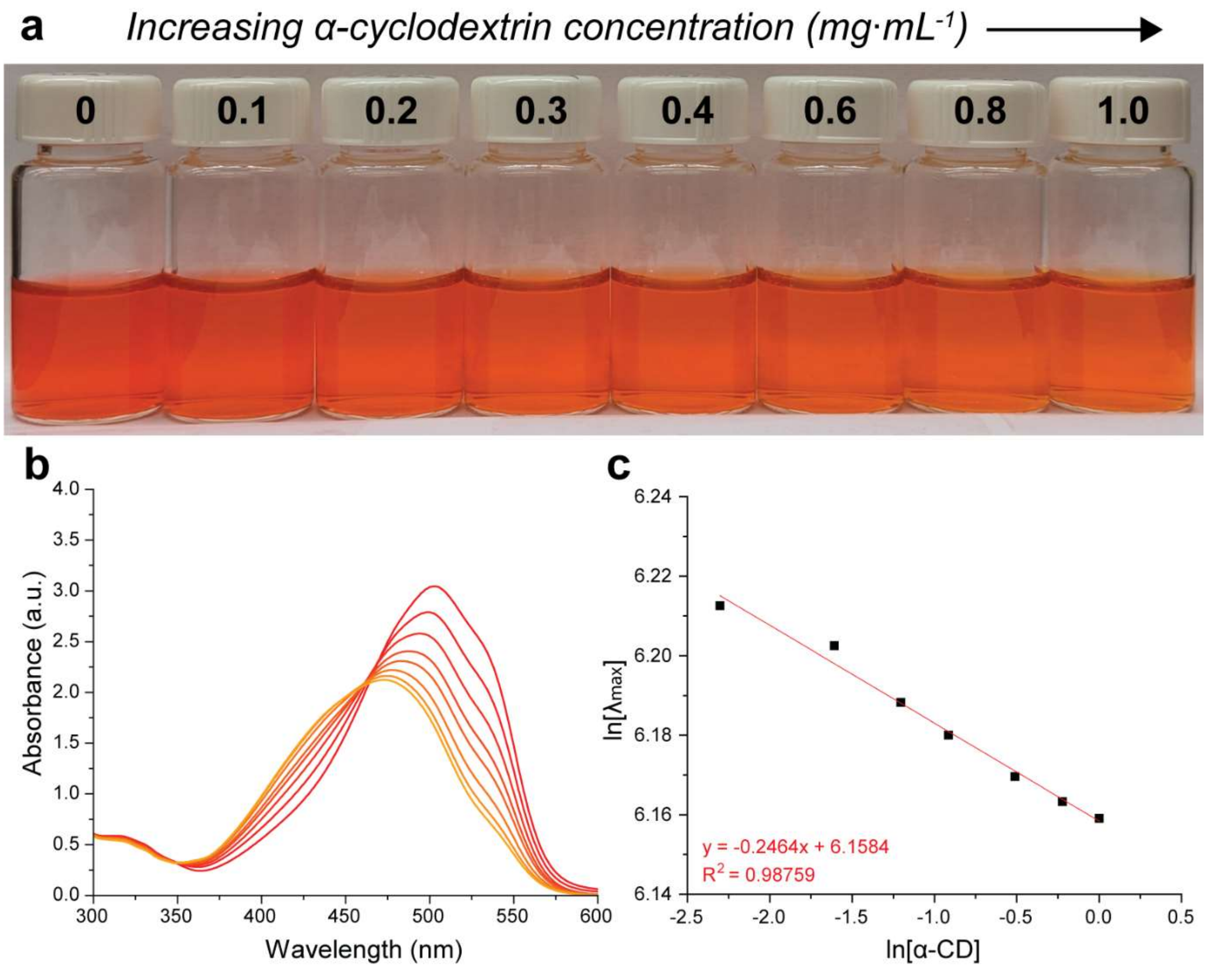

Figure S5. Photometric titration calibration curve for calculation of α-CD grafting extent through monitoring the shift in peak wavelength of methyl orange absorbance. a) Photo illustrating the effect of adding α-CD to an aqueous solution of methyl orange at constant concentration. Note that BC has not been added to the samples shown in a) to more clearly show the color change. B) UV-vis traces for calibration curve standards. c) Generated linear calibration curve for calculating the concentration of α-CD.

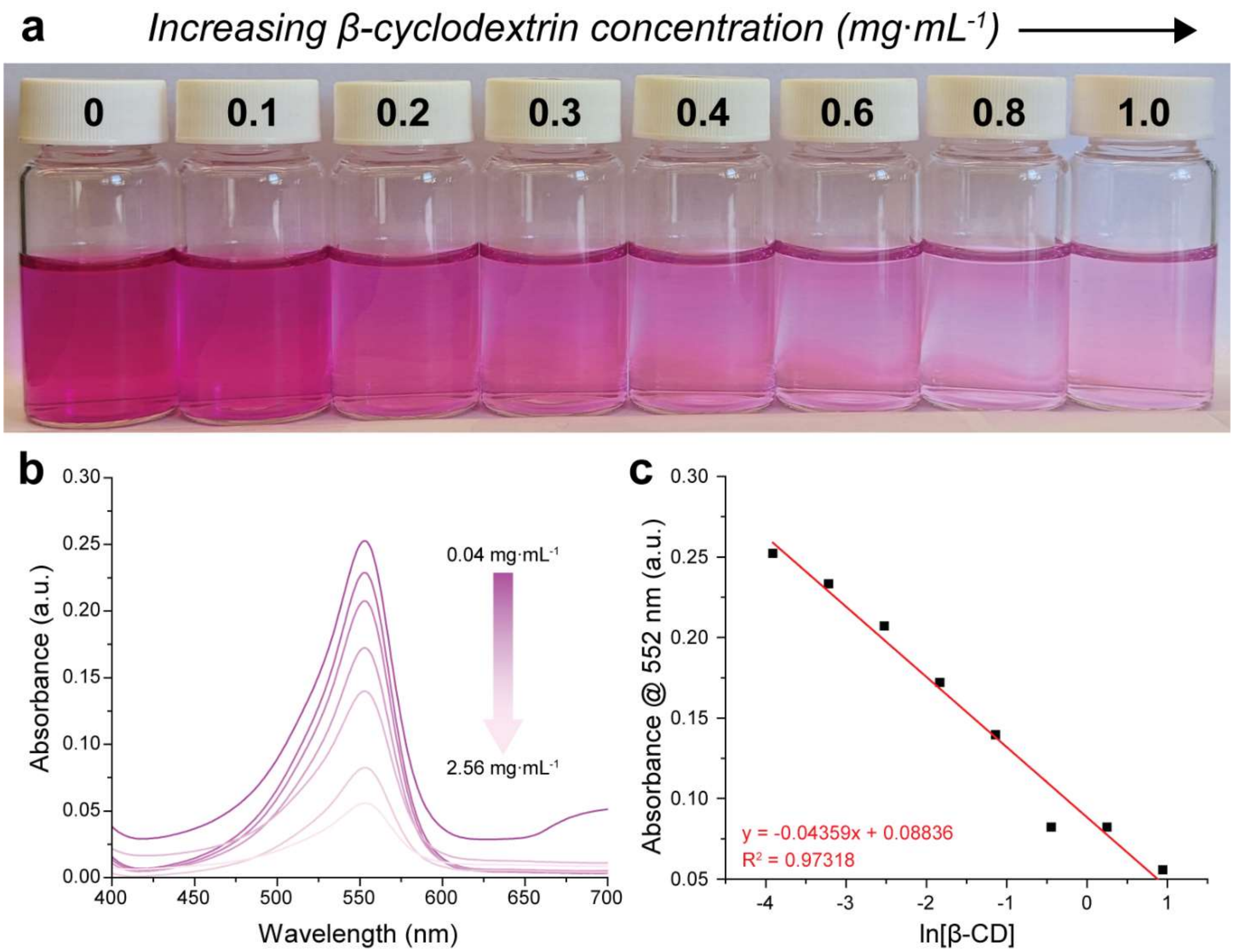

Figure S6. Photometric titration calibration curve to calculate the extent of β-CD grafting through monitoring the attenuation of phenolphthalein absorbance at 552 nm. a) Photo illustrating the effect of adding β-CD to an aqueous, pH 10 solution of phenolphthalein at constant concentration. Note that BC has not been added to the samples shown in a) to more clearly show the color change. b) UV-vis traces for calibration curve standards. c) Generated linear calibration curve for calculating the concentration of β-CD.

Supplementary Table S1. Calibration curve for the UV-vis quantification of α-CD content in α-CD-BC with methyl orange.* Grey highlighted row represents the α-CD-BC sample.

[Methyl orange] (mM)	[α-CD] (mg mL^{-1})	[BC] (mg mL^{-1})	[α-CD-BC] (mg mL^{-1})
1	0	1	0
1	0.1	1	0
1	0.2	1	0
1	0.3	1	0
1	0.4	1	0
1	0.6	1	0
1	0.8	1	0
1	1.0	1	0
1	0	0	1

* All solutions prepared in pH 3 citrate-phosphate buffer.

Supplementary Table S2. Calibration curve for the UV-vis quantification of β-CD content in β-CD-BC with phenolphthalein. Grey highlighted row represents the β-CD-BC sample.

[Phenolphthalein] (mM)	[Na_2CO_3] (mM)	[β-CD] (mg mL^{-1})	[BC] (mg mL^{-1})	[β-CD-BC] (mg mL^{-1})
0.1	20	0	1	0
0.1	20	0.01	1	0
0.1	20	0.02	1	0
0.1	20	0.04	1	0
0.1	20	0.08	1	0
0.1	20	0.16	1	0
0.1	20	0.32	1	0
0.1	20	0.64	1	0
0.1	20	1.28	1	0
0.1	20	2.56	1	0
0.1	20	0	0	1

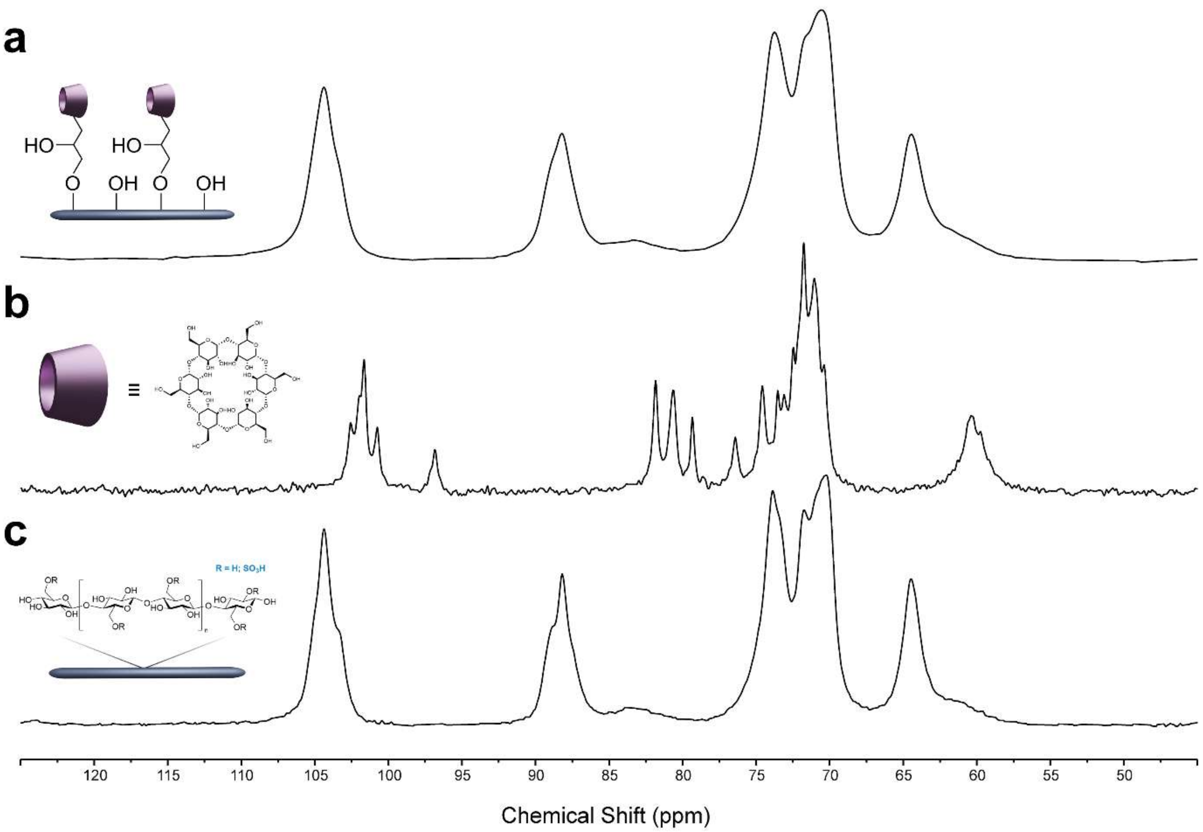

Figure S7. Solid state ^{13}C CP/MAS NMR spectra for a) α-CD-BC, b) α-CD, and c) BC.

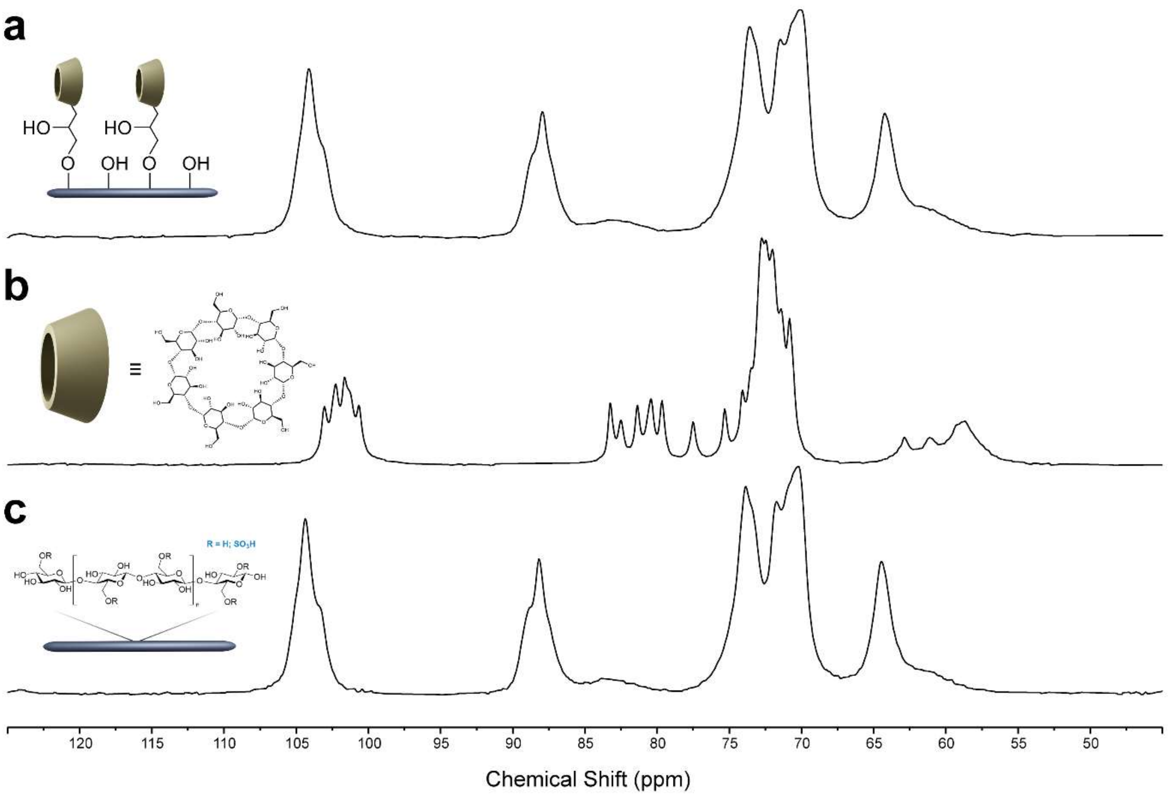

Figure S8. Solid state ^{13}C CP/MAS NMR spectra for a) β-CD-BC, b) β-CD, and c) BC.

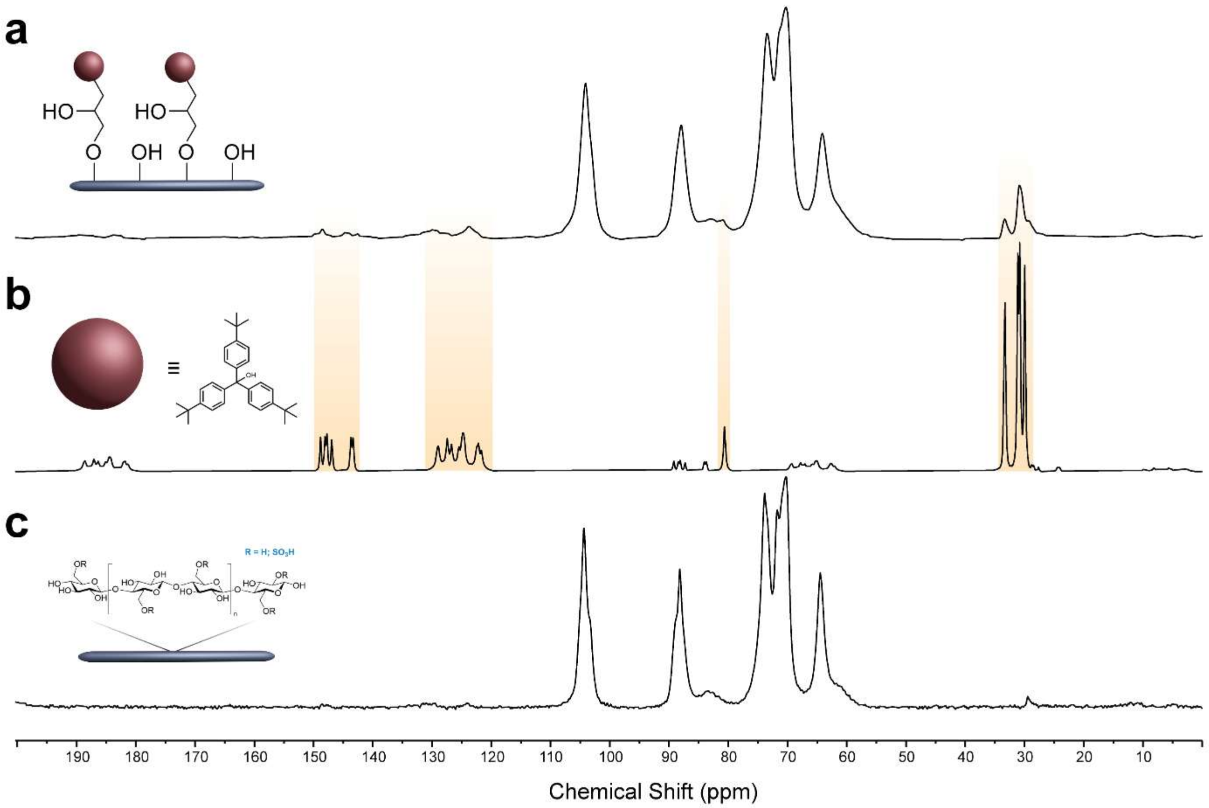

Figure S9. Solid state ^{13}C CP/MAS NMR spectra for a) ST-BC, b) ST, and c) BC. Selected peaks are highlighted in a) and b) to illustrate the successful grafting of ST onto BC.

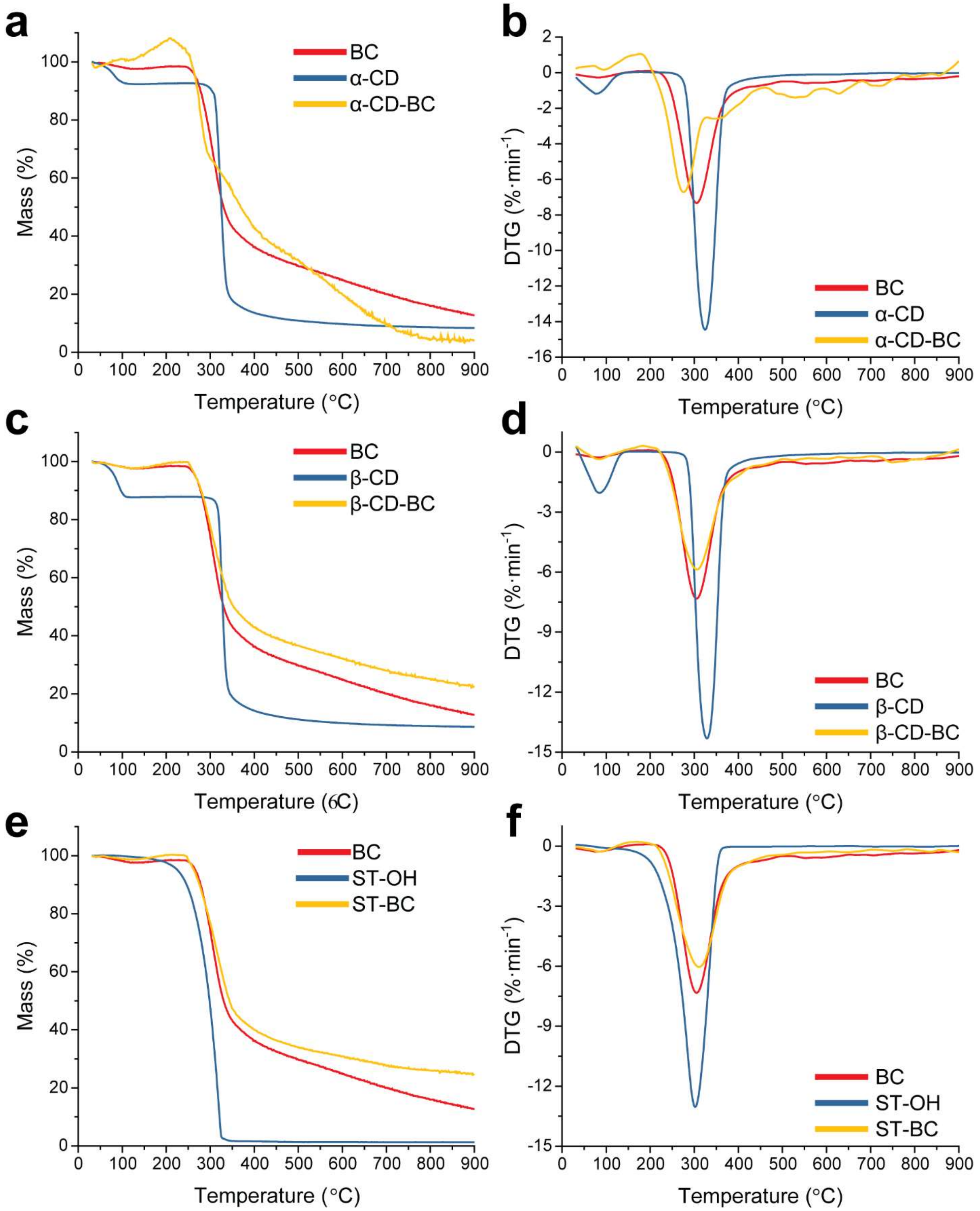

Figure S10. a) TGA and b) differential TG traces for α-CD-BC, c) TGA and d) differential TG traces for β-CD-BC, and e) TGA and f) differential TG traces for ST-BC. DTG traces have been smoothed in OriginPro 2021b using the Loess algorithm.

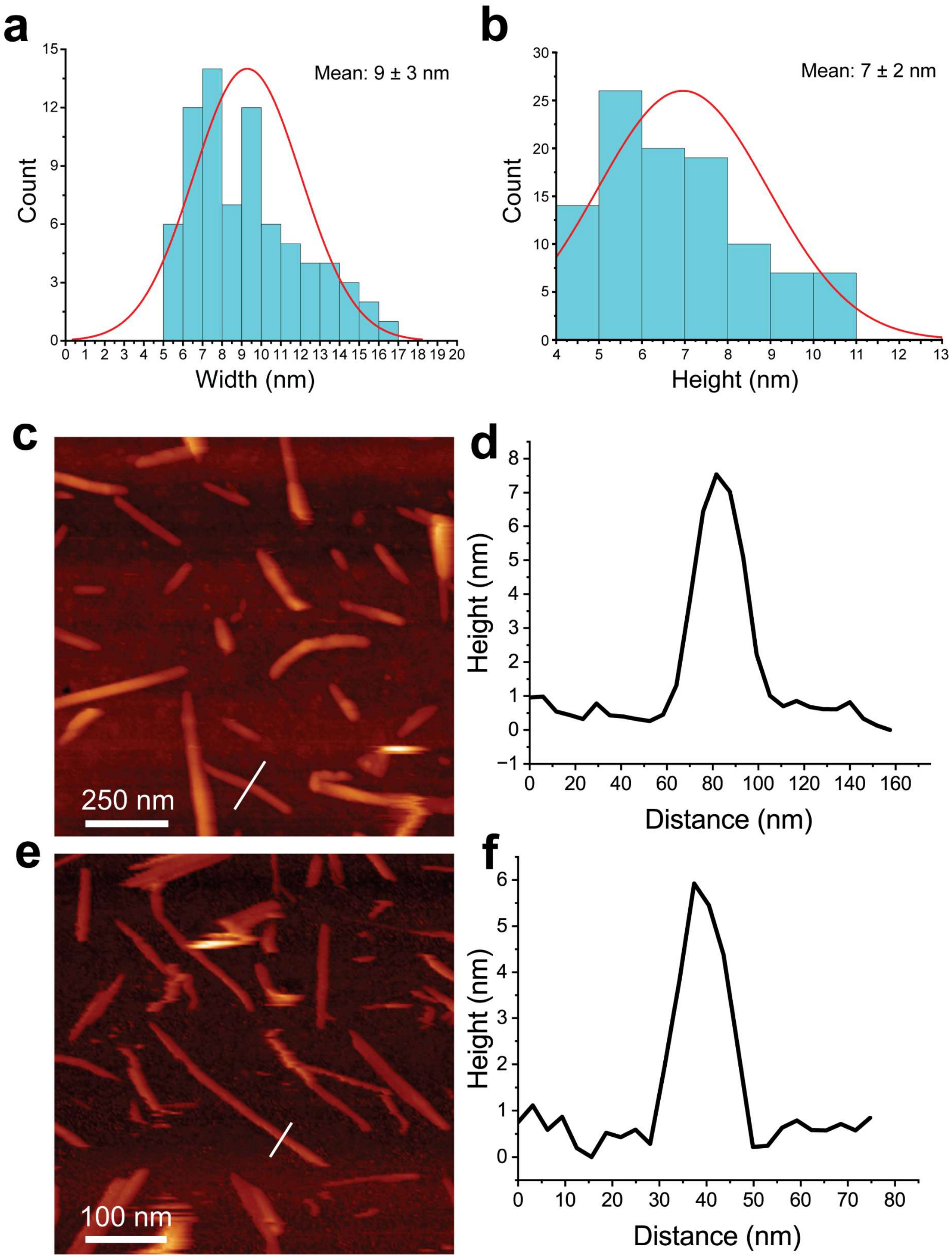

Figure S11. a) Width distribution of α-CD-BC nanocrystals as determined from TEM measurements. b) Height distribution of α-CD-BC nanocrystals as determined from FM-AFM height profile measurements. c,e) FM-AFM images of α-CD-BC and β-CD-BC affixed on HOPG substrate and d,f) corresponding height profiles along the paths outlined in c,e), respectively.

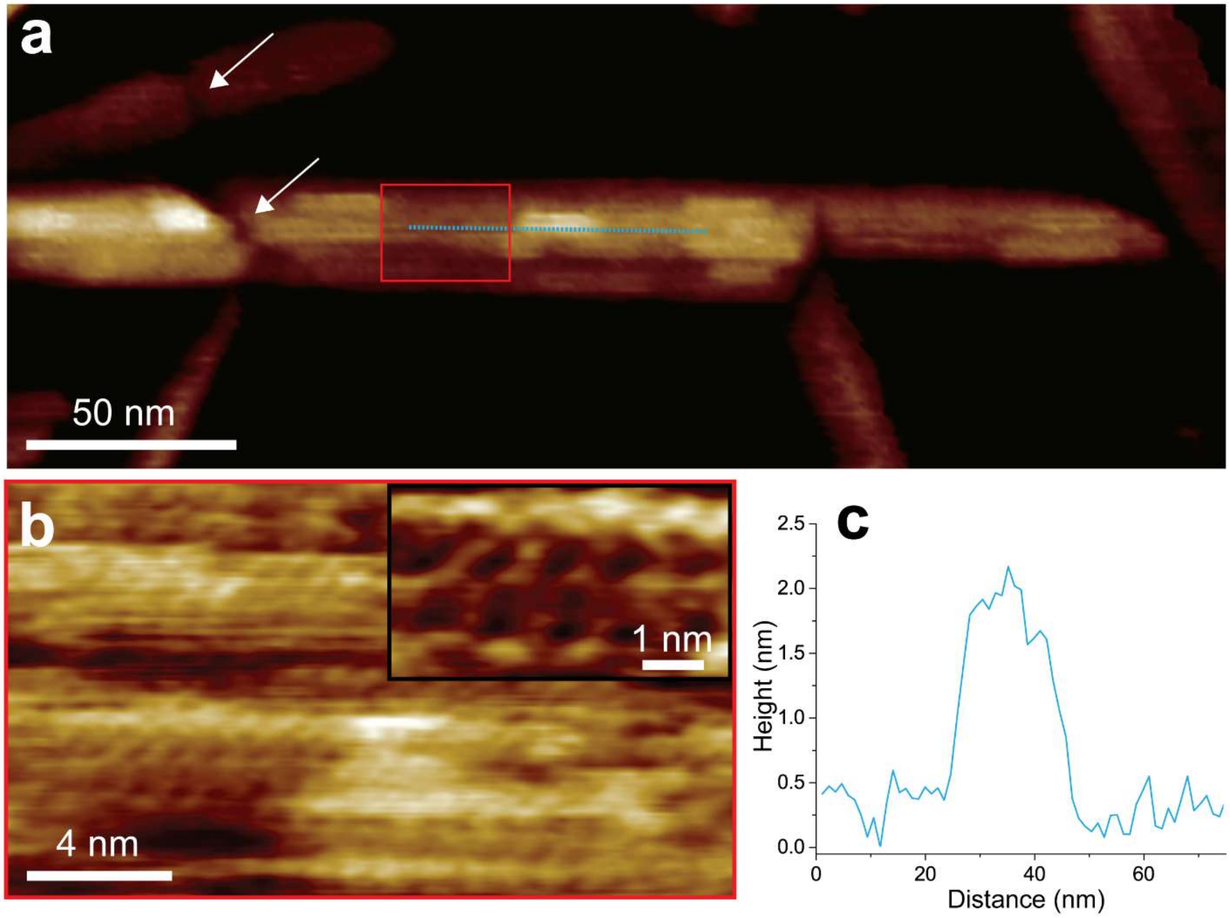

Figure S12. Additional FM-AFM images of unfunctionalized BC. a) Image of a single BC nanocrystal. White arrows illustrate large defects in the crystalline structure. Additionally, large bright patches are observed, which attributed to water adlayers or loosely-bound water at the interface, making it difficult to resolve the cellulose lattice. b) High-resolution FM-AFM image of the red-outlined, predominantly clean area in panel a) revealing the cellobiose repeating unit (inset). c) Height profile from left to right along the blue dotted line shown in a), outlining a diffuse bright patch attributed to loosely bound water or water adlayers.

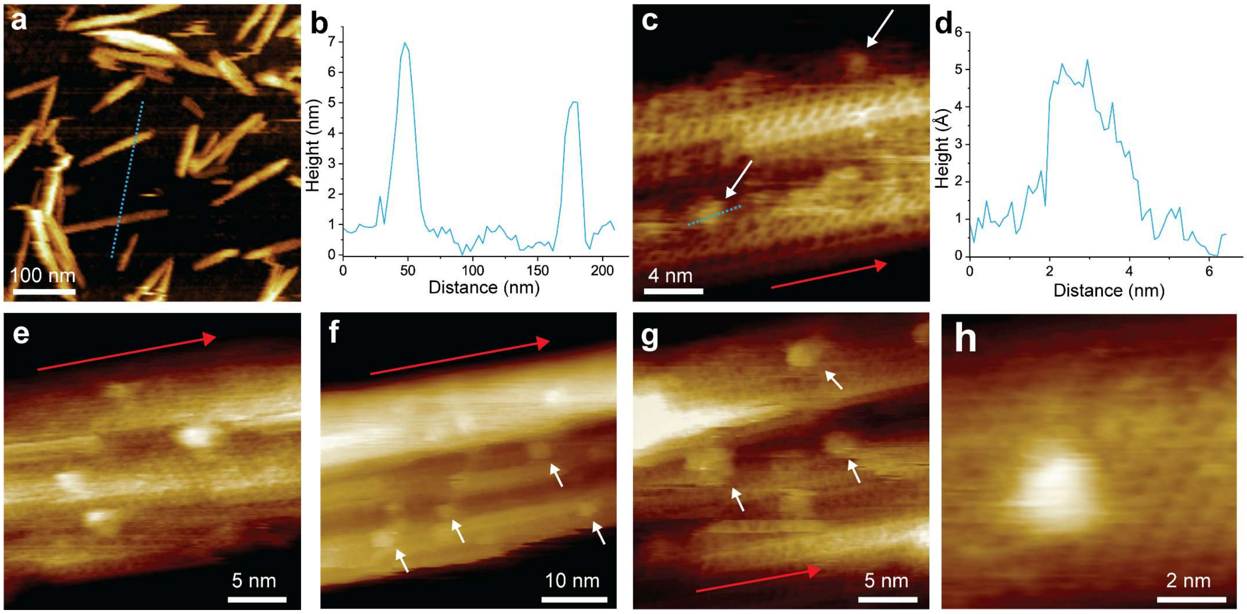

Figure S13. Additional high-resolution FM-AFM images of α-CD-BC. a) Low magnification overview image and b) the corresponding height profile taken along the dotted blue line, demonstrating that the BC nanocrystal width is relatively unaltered compared to unfunctionalized BC. c) High-resolution FM-AFM image of α-CD grafted on the surface of BC and d) the corresponding height profile along the dotted blue line. e-h) Additional high-resolution images of α-CD-BC obtained from different BC fibers. White arrows indicate grafted α-CD molecules of uniform size, and red arrows indicate the direction of cellulose molecular chains.

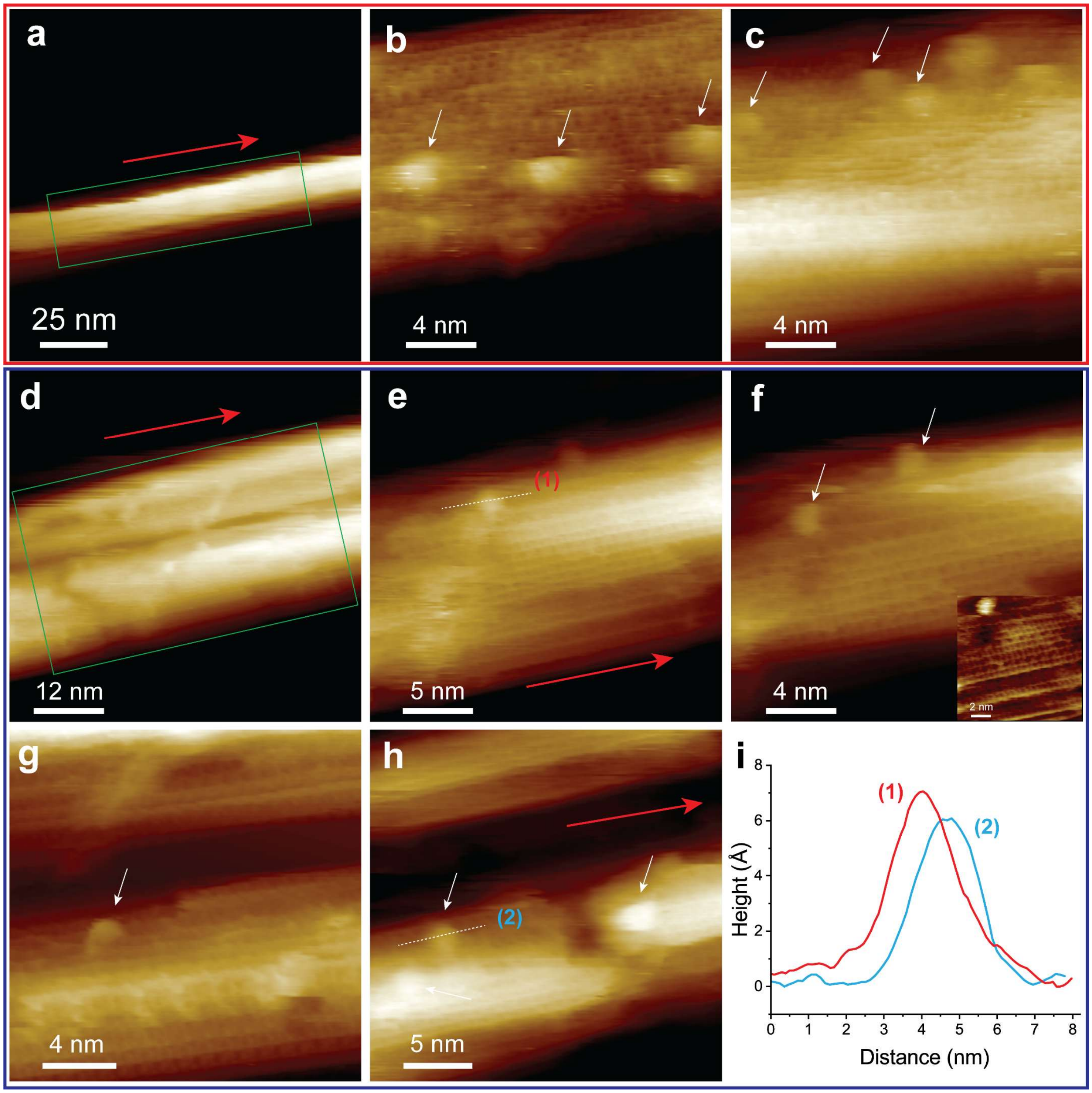

Figure S14. Additional high-resolution FM-AFM images of α-CD-BC. Images a-c) and d-h) represent sets of images each taken from two different BC fibers. The height profiles in i) correspond to the dotted white lines in e) and h), and are labeled and color-coded respectively. White arrows represent grafted α-CD molecules, the green boxes represent the scanned region along each fiber, and red arrows represent the direction of the cellulose molecular axis.

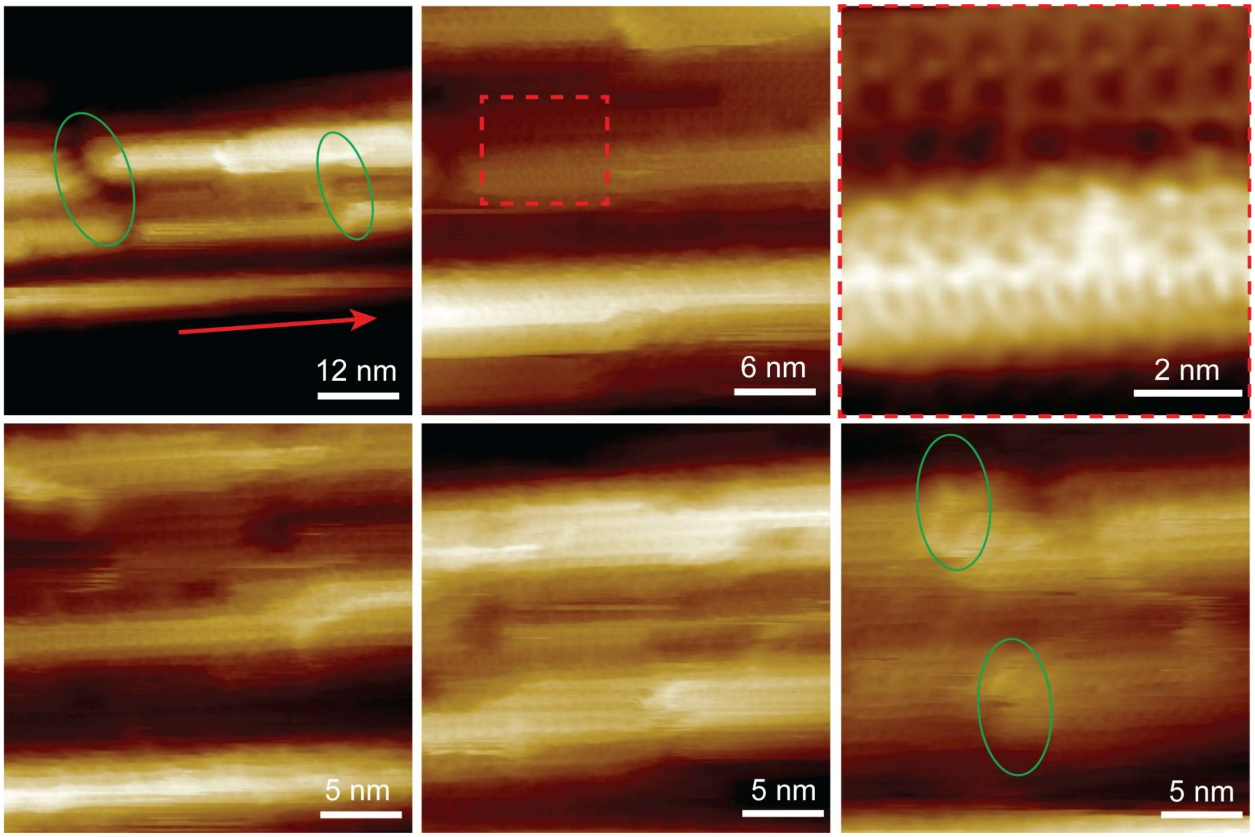

Figure S15. Assortment of high-resolution FM-AFM images of processing control sample 1; i.e. BC samples subjected to identical processing as compared to grafted BC samples, but forgoing the addition of α-CD. No features similar to grafted α-CD can be observed; only large characteristic defects (highlighted by green circles) and clean BC surfaces are visible.

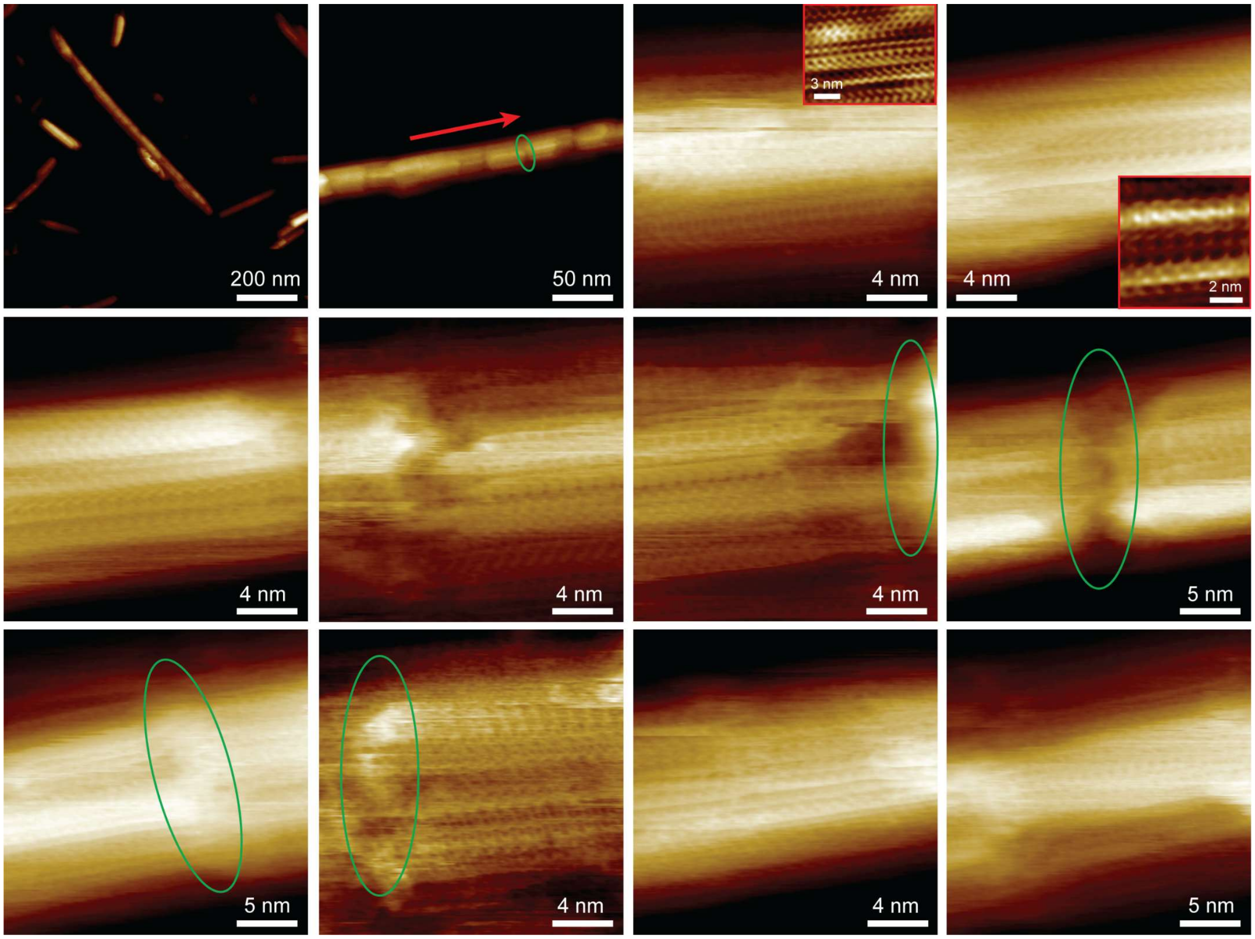

Figure S16. Assortment of high-resolution FM-AFM images of processing control sample 2; i.e. BC samples subjected to identical processing conditions as compared to grafted BC samples, but forgoing the addtion of epichlorohydring. No features similar to grafted α-CD can be observed; only large characteristic defects (highlighted by green circles) and clean BC surfaces are visible.

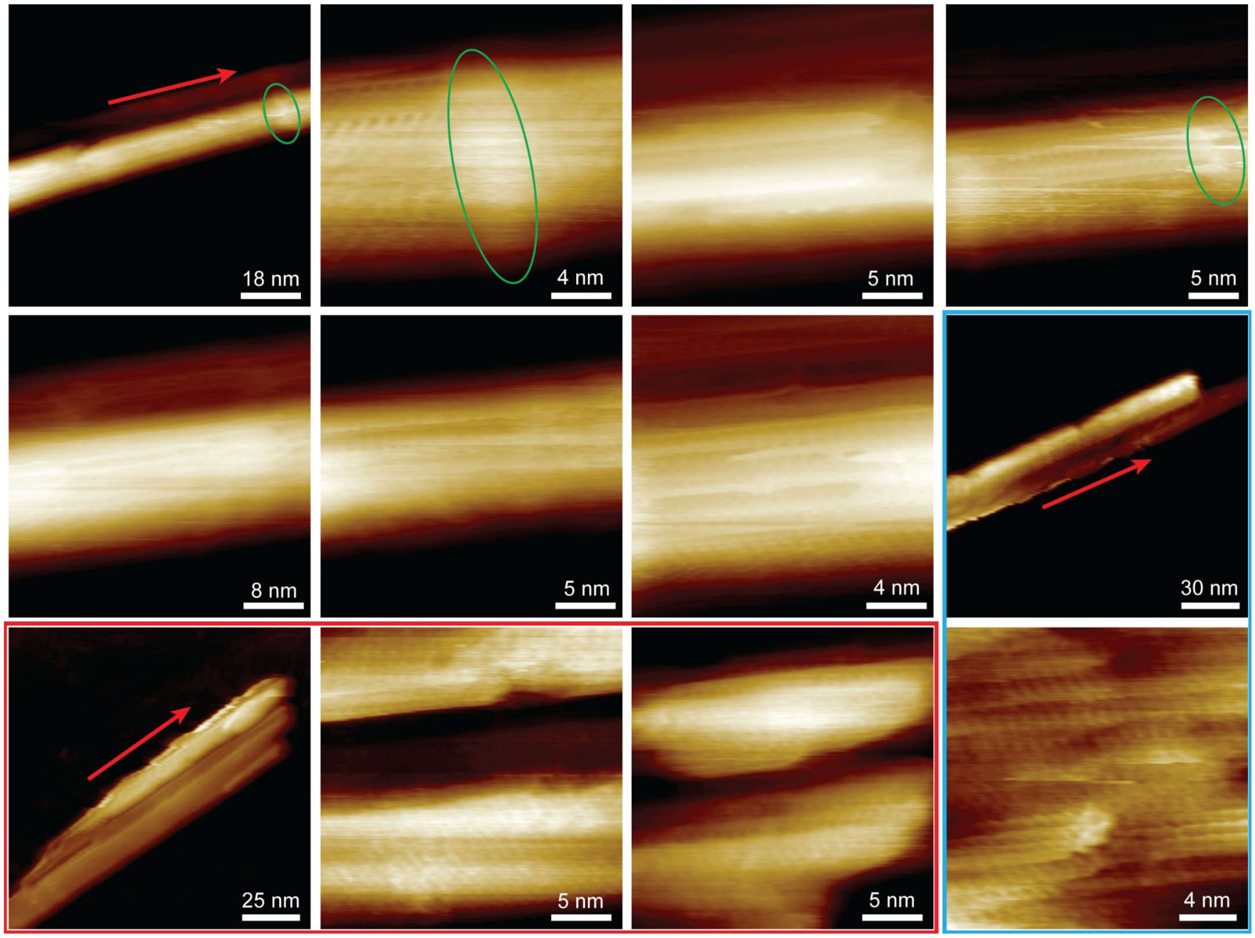

Figure S17. Assortment of high-resolution FM-AFM images of control sample 3 prepared by simple mixing of BC and α-CD. α-CD cannot be observed on any of the imaged particles. Only large characteristic defects (highlighted by green circles) and clean BC surfaces are visible. Red arrows represent the direction of the cellulose molecular axis. Images grouped by red and blue boxes were taken from the same nanocrystal.

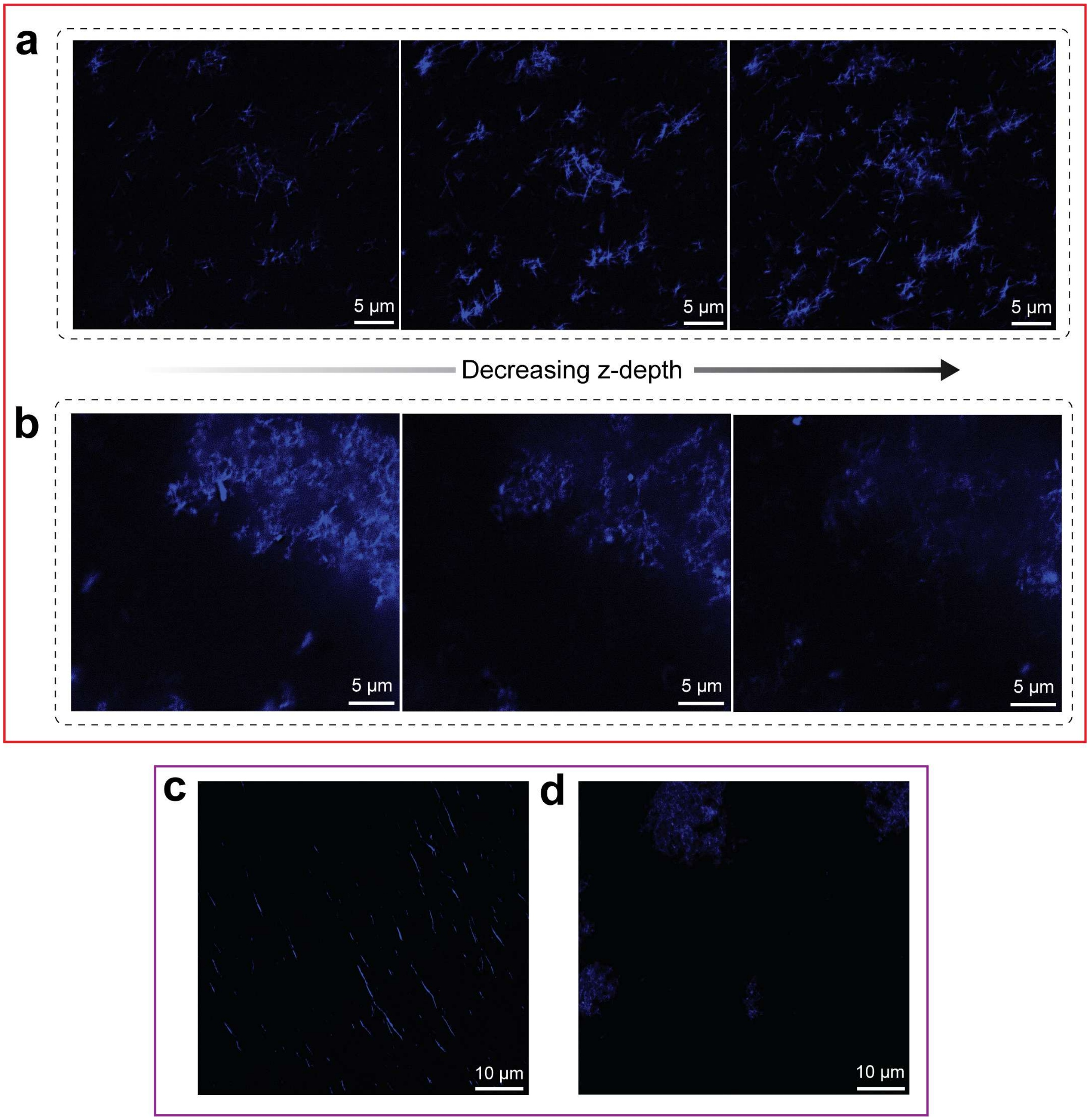

Figure S18. Extended resolution light microscopy images of a,c) α-CD-BC and b,d) unfunctionalized BC. Panels a-b) were captured using an Olympus/Evident IXplore Spin SR system, and panels c-d) were captured using a Leica Stellaris 8 Confocal Laser Microscope. All samples were stained with calcofluor white at pH 10 before imaging.

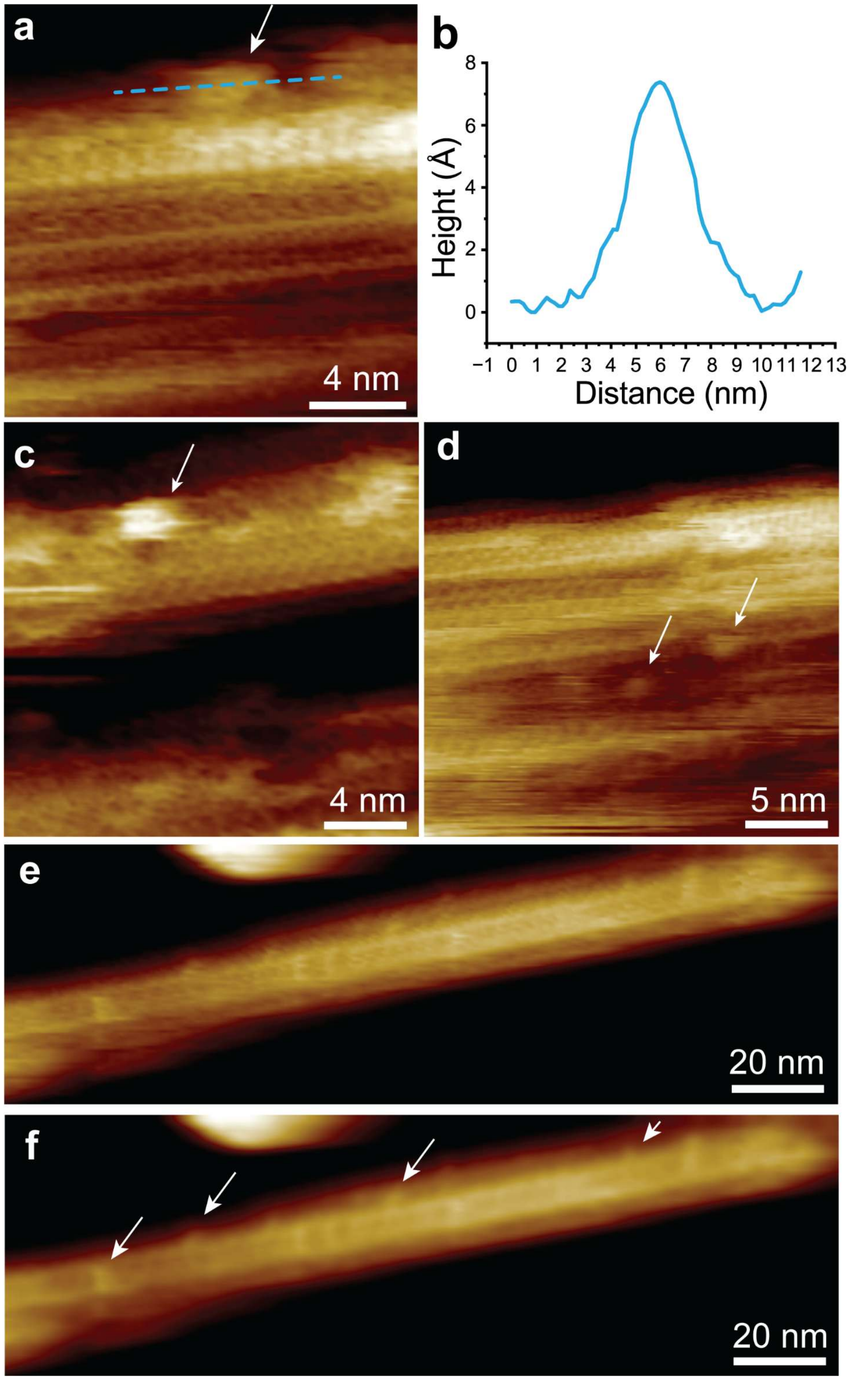

Figure S19. Additional high-resolution FM-AFM images of β-CD-BC, illustrating a) β-CD grafted at the edge of a BC particle, and b) height profile along the blue dotted line shown in b). c-f) Further high-resolution images of β-CD-BC. The image in f) is a smoothed version of e). White arrows indicate grafted β-CD, and the red arrow shows the direction of the cellulose molecular chains.

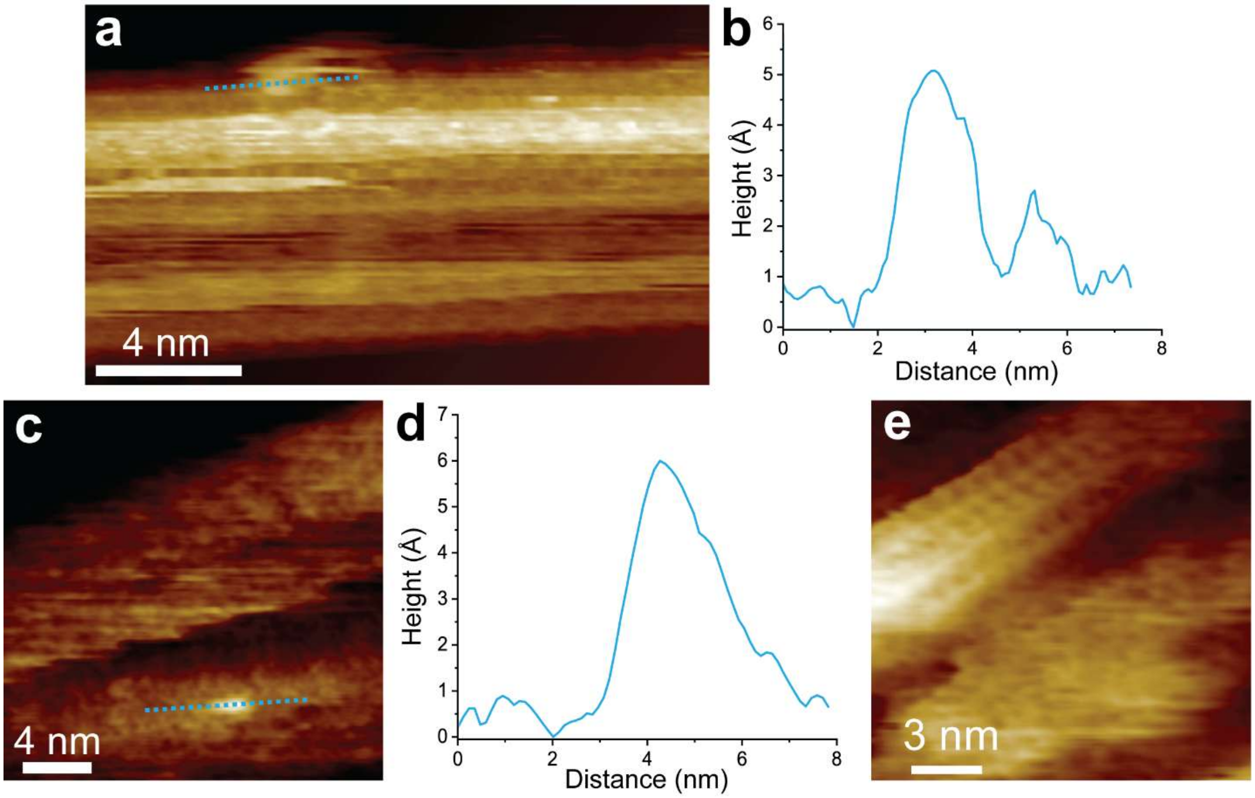

Figure S20. Additional higher resolution FM-AFM images of ST-BC. a) Nanocrystal surface with a single grafted ST molecule on the top edge and b) corresponding height profile along the dotted blue line. c) Nanocrystal surface with a single grafted ST molecule near the bottom and d) corresponding height profile along the dotted blue line. e) High-resolution FM-AFM image of an ST-grafted BC nanocrystal, showing the cellulose crystal lattice with defects visible in the bottom half of the image.

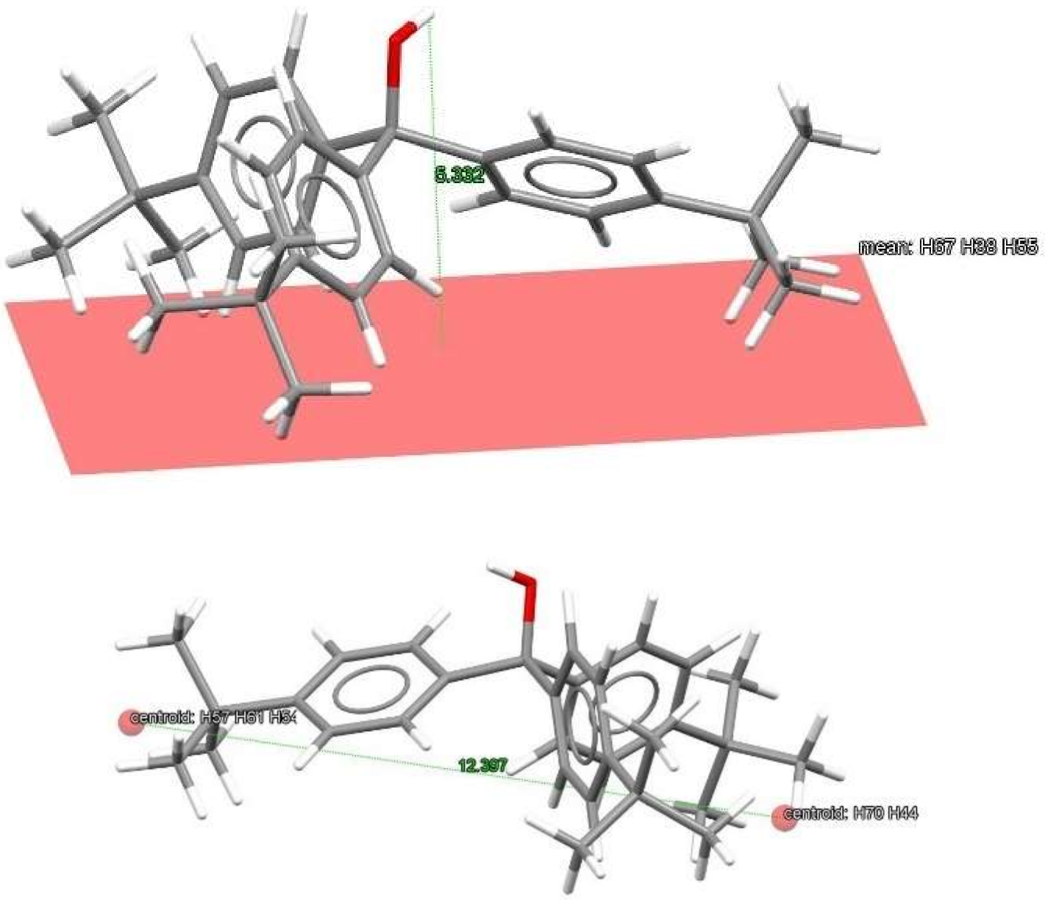

Figure S21. Dimensions of the optimized structure of ST (Å) obtained from DFT calculations at the B3LYP/6-31G(d,p) level of theory.

Supplementary Table S3. Cartesian coordinates (Å) of the optimized geometry of ST obtained from DFT calculations at the B3LYP/6-31G(d,p) level of theory.

Atom	x	y	z
C	-0.71850000	-3.12380000	-0.91500000
C	-1.69670000	-3.75380000	-0.13820000
C	-2.04810000	-3.12620000	1.07020000
C	-1.46010000	-1.93390000	1.47750000
C	-0.47860000	-1.31420000	0.69230000
C	-0.11750000	-1.92880000	-0.50750000
C	-2.37670000	-5.07230000	-0.54880000
C	-1.86260000	-5.59950000	-1.90160000
C	-2.10010000	-6.15100000	0.52520000
C	-3.90310000	-4.84910000	-0.66780000
C	0.11470000	0.03040000	1.14240000
C	1.55580000	0.22940000	0.63740000
C	-0.84150000	1.16330000	0.71400000
C	-1.37100000	2.05310000	1.64830000
C	-2.23870000	3.07970000	1.26250000
C	-2.61570000	3.25950000	-0.07230000
C	-2.08550000	2.35110000	-1.00600000
C	-1.22610000	1.32640000	-0.62560000
C	1.98010000	1.31750000	-0.12530000
C	3.32320000	1.46110000	-0.49380000
C	4.29420000	0.53090000	-0.11190000
C	3.85820000	-0.55820000	0.66650000
C	2.52770000	-0.70580000	1.03490000
C	5.77830000	0.65480000	-0.50020000
C	6.05910000	1.91070000	-1.34630000
C	6.64100000	0.73410000	0.78180000
C	6.19880000	-0.58580000	-1.32360000
C	-3.56800000	4.37590000	-0.53650000
C	-4.03920000	5.26480000	0.62970000
C	-2.84350000	5.27560000	-1.56570000
C	-4.81690000	3.74630000	-1.19800000
O	0.17770000	-0.04560000	2.58090000
H	-0.40000000	-3.56110000	-1.85440000
H	-2.79990000	-3.57580000	1.71260000
H	-1.75350000	-1.47840000	2.41640000
H	0.65120000	-1.48570000	-1.13280000

Atom	x	y	z
H	-2.37450000	-6.53420000	-2.15240000
H	-2.05320000	-4.89060000	-2.71400000
H	-0.78820000	-5.80880000	-1.87560000
H	-2.58370000	-7.09580000	0.25170000
H	-2.48050000	-5.85430000	1.50700000
H	-1.02570000	-6.33580000	0.62610000
H	-4.12990000	-4.09480000	-1.42820000
H	-4.40450000	-5.78120000	-0.95260000
H	-4.33980000	-4.51150000	0.27660000
H	-1.12570000	1.93220000	2.69730000
H	-2.62270000	3.73950000	2.03220000
H	-2.35360000	2.43660000	-2.05510000
H	-0.85840000	0.63790000	-1.38010000
H	1.26700000	2.07030000	-0.44120000
H	3.59810000	2.32500000	-1.08800000
H	4.57220000	-1.30840000	0.99410000
H	2.22670000	-1.55340000	1.64240000
H	7.12350000	1.95510000	-1.59820000
H	5.49780000	1.90260000	-2.28630000
H	5.80940000	2.83000000	-0.80670000
H	7.70260000	0.81500000	0.52240000
H	6.51910000	-0.15260000	1.41060000
H	6.37050000	1.60890000	1.38200000
H	5.60980000	-0.66310000	-2.24320000
H	7.25650000	-0.51800000	-1.60210000
H	6.06180000	-1.51370000	-0.76080000
H	-4.70960000	6.04370000	0.25250000
H	-4.59050000	4.69130000	1.38200000
H	-3.20060000	5.76330000	1.12660000
H	-3.51080000	6.07240000	-1.91320000
H	-2.51700000	4.70930000	-2.44290000
H	-1.95850000	5.74330000	-1.12210000
H	-5.35450000	3.10760000	-0.48970000
H	-5.50360000	4.52910000	-1.53980000
H	-4.55310000	3.13350000	-2.06490000
H	0.76560000	0.66470000	2.87230000

SUPPLEMENTARY DISCUSSION 2

To accurately discern grafted molecules from nanocellulos defects or loosely-bound water adlayers, it is necessary to thoroughly examine the different types of features that can be observed under AFM investigation. Therefore, in addition to the additional images provided in **Figures S12-S17, S19-S20**, the information below should serve as a guideline for the interpretation of AFM images of grafted nanocellulose surfaces.

Appearance of clean, non-functionalized, defect-free BC surfaces:

The images below represent smooth, clean BC surfaces, where no surface defects or adsorbed contaminants can be seen. The periodic crystal lattice of nanocellulose can be visualized, revealing a cellobiose repeat unit of approximately 1 nm. Additionally, the interval between glucose units, around 0.53 nm, is also observable, reflecting the molecular organization within the crystalline cellulose structure.

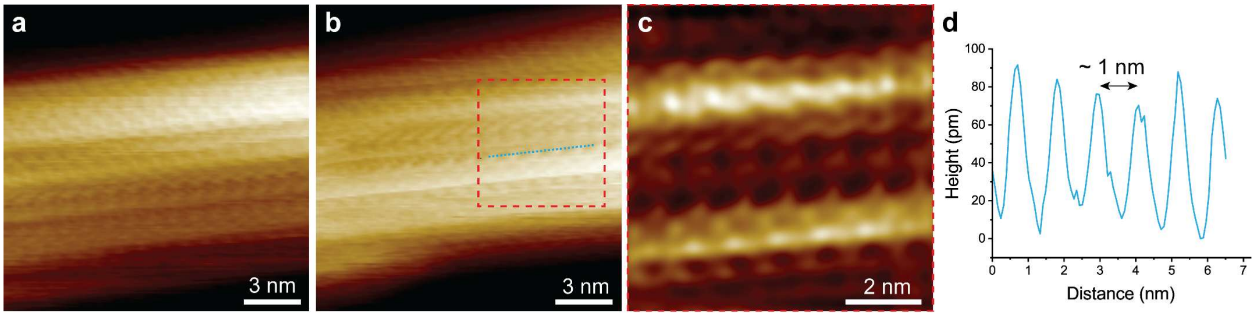

Figure S22. a-c) High resolution FM-AFM images of a clean BC surface lacking any significant defects and d) height profile along the dotted blue line in b) illustrating the spacing of the cellulose lattice on the BC surface.

Appearance of BC with irregular/layered surfaces:

During processing, layers of cellulose can sometimes be stripped away from the surface of the BC nanocrystals. This gives rise to large, bright defects under AFM. Below, examples of these types of defects are shown *via* a series of images taken along the surface of a single nanocrystal.

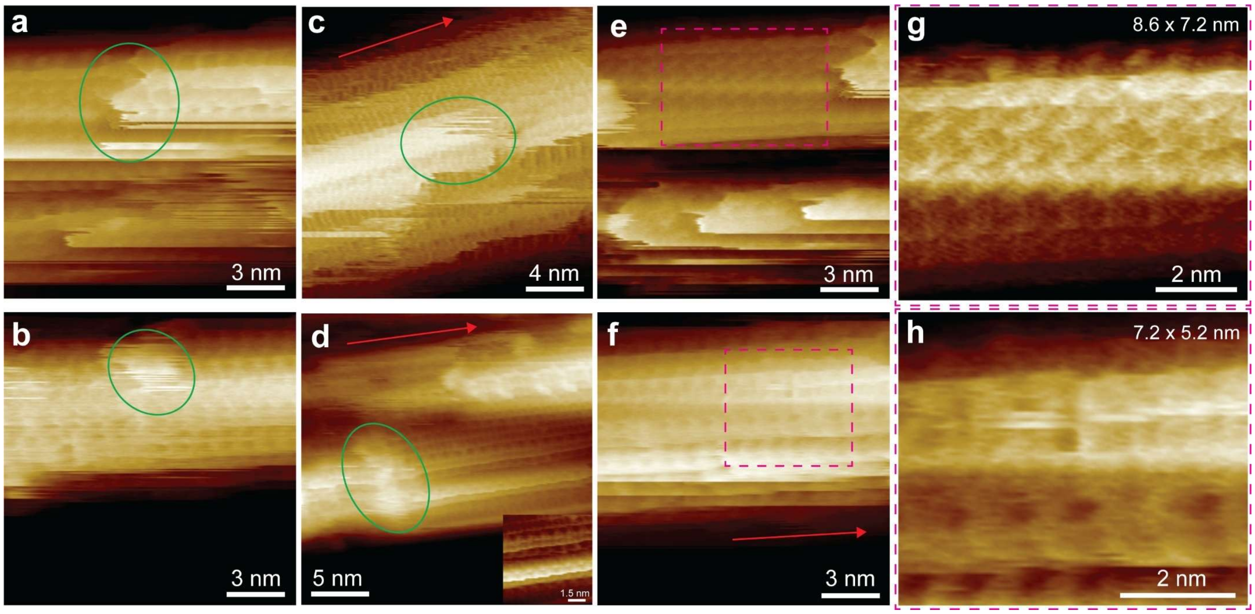

Figure S23. High resolution FM-AFM images illustrating large defects that manifest as stacked layers of cellulose chains. The BC surfaces are clean, but large defects resulting from processing or the irregular surface of nanocellulose are present. Green circles in a-d) outline some of these defects, and red rectangles in g-h) represent clean areas shown at higher magnification in panels e-f), respectively.

In these images, the clean surface of BC along with the crystalline cellulose lattice can clearly be seen, but layered structures at the surface of the nanocrytal also persist. These large defects are quite easily distinguishable from grafted molecules. There are fluctuations at the boundary between the layered cellulose structures, which may arise either from the dynamic motion of cellulose chains or from fluctuations in the water hydration layer at the interface. It is not clear whether these fluctuations are due to the cellulose itself or the associated interfacial water, as both the cellulose chains and the hydration layers can exhibit such behavior at these boundaries. Also, there are bright, disordered domains almost perpendicularly oriented with respect to the cellulose molecular axis. These are typical defects commonly observed on cellulose surfaces in water.

Appearance of disordered cellulose domains on the BC surface:

Disordered cellulose domains can appear as clusters of small particles on the surface, possibly caused by hydrolysis products produced during BC fabrication. They possess some characteristics that make them distinguishable from grafted molecules:

1. They are much smaller in height, often <0.3 nm (<3 Å).
2. They are often found in large clusters on the surface, not attached at regular intervals.

Some examples of these types of defects are shown below:

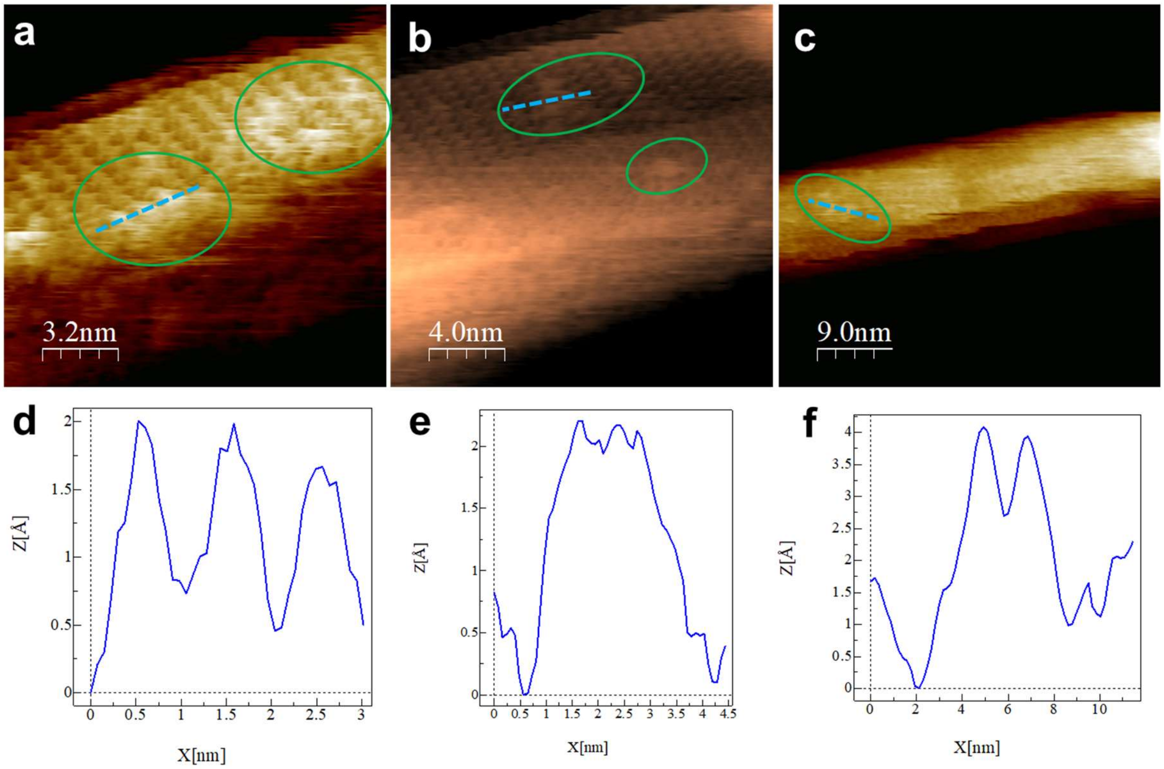

Figure S24. a-c) High-resolution FM-AFM images of the non-functionalized surface of several BC nanocrystals, illustrating the presence of disordered cellulosic domains at the surface (highlighted with green circles) and d-f) corresponding height profiles highlighted by the blue dotted lines in panels a-c), respectively.

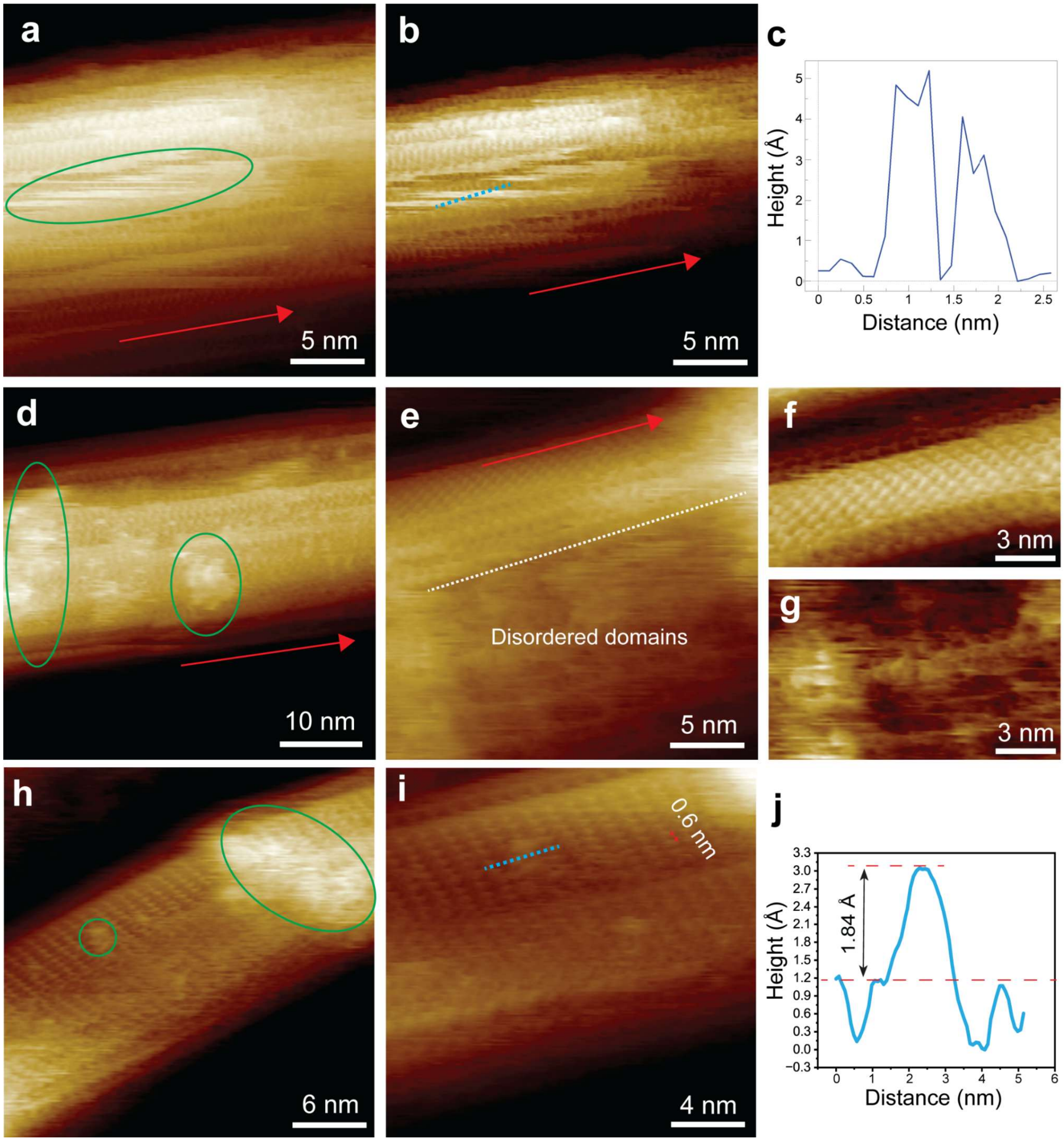

Figure S25. Additional high-resolution FM-AFM images providing detailed characterization of potential surface defects. a-b) Section of a BC fiber with defects running down the length, and c) height profile along the dotted blue line in b). d) Another section of BC fiber illustrating several large defect patches. e) A section of BC fiber illustrating the surface texture that results from disordered cellulosic domains. Comparison of f) a well-ordered section of BC surface in which the cellulose crystal lattice is visible and g) a disordered section. h-i) Further images of defects on the BC surface, with the height profile of a defect (outlined with a dotted blue line in i)) shown in panel j). Defects are outlined with green circles, and red arrows show the direction of the cellulose crystal lattice.

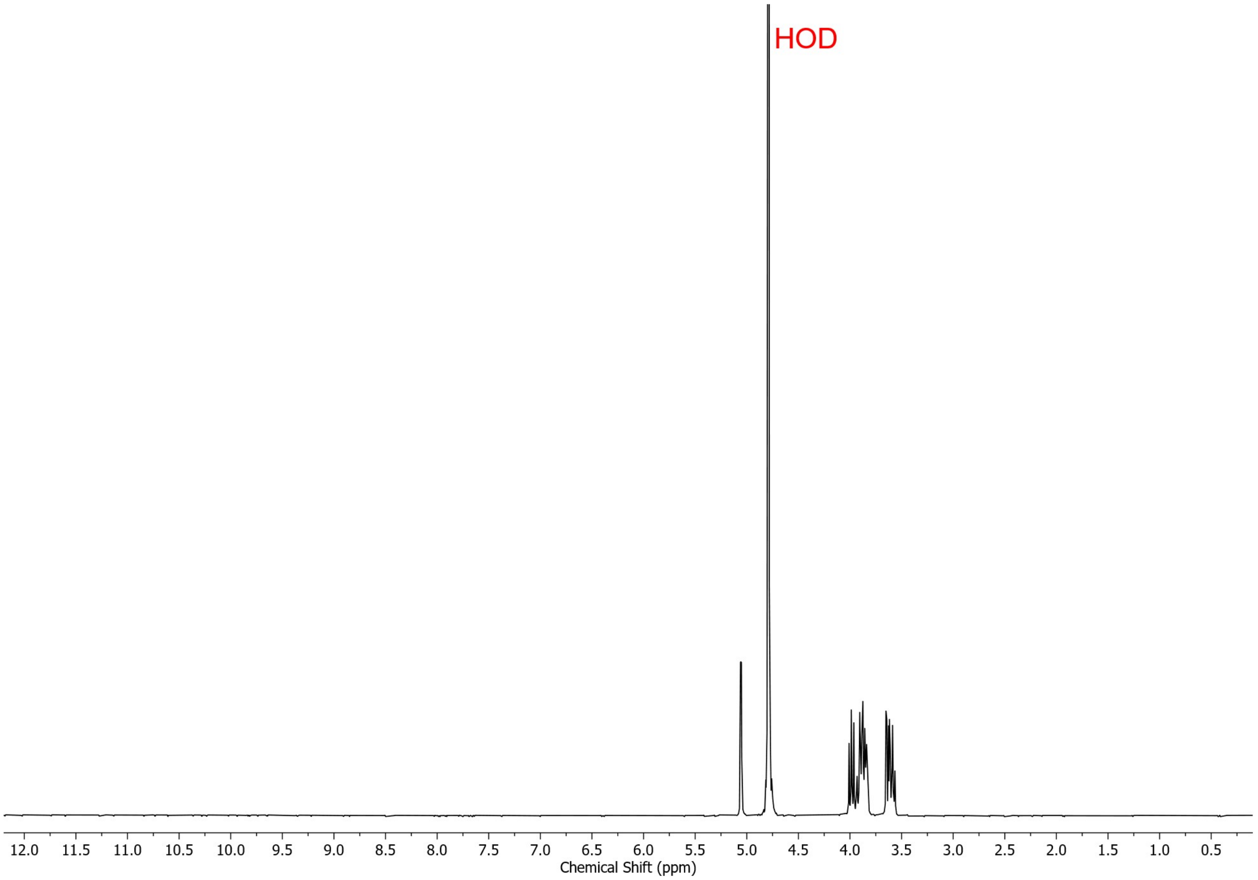

Figure S26. ^{1}H NMR spectrum (400 MHz, D_2O) of α-CD.

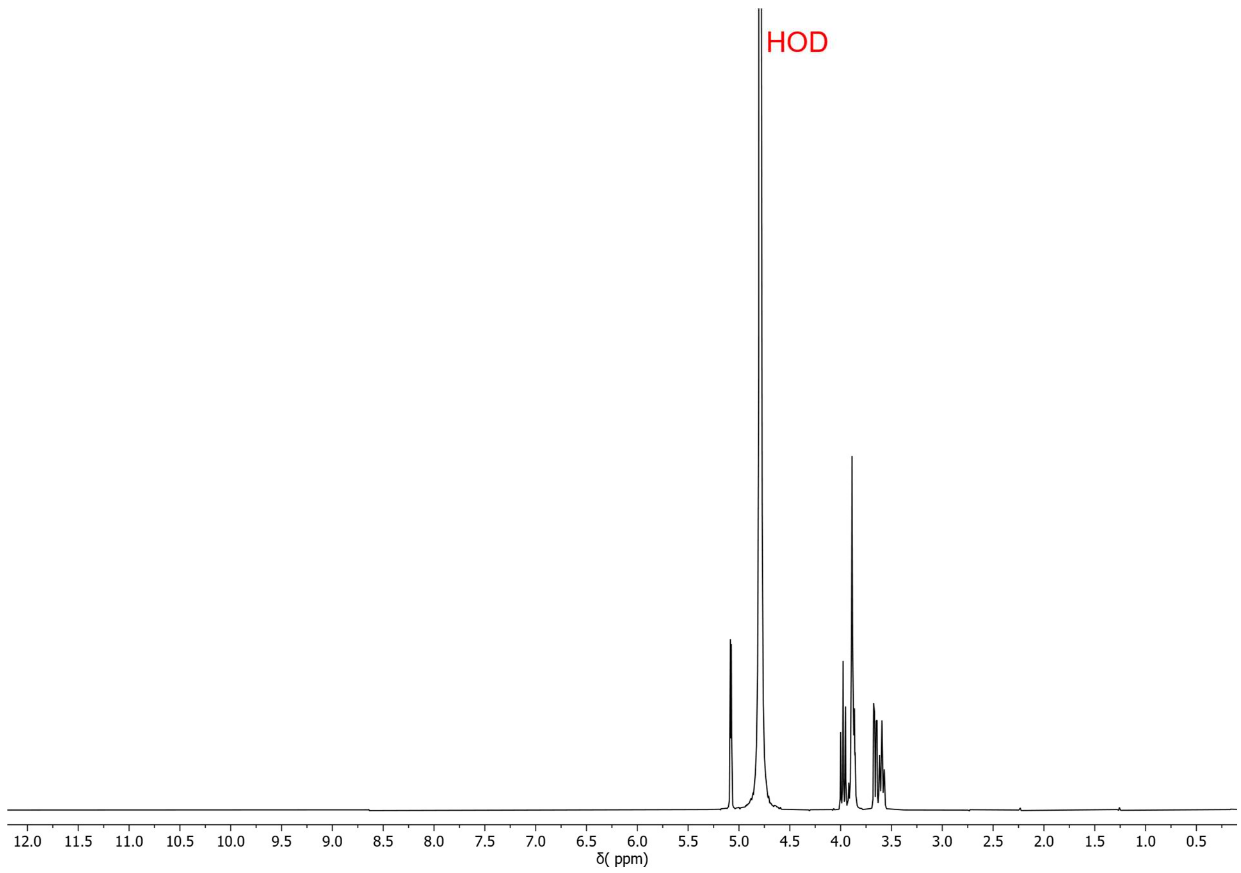

Figure S27. ^{1}H NMR spectrum (400 MHz, D_2O) of β-CD.

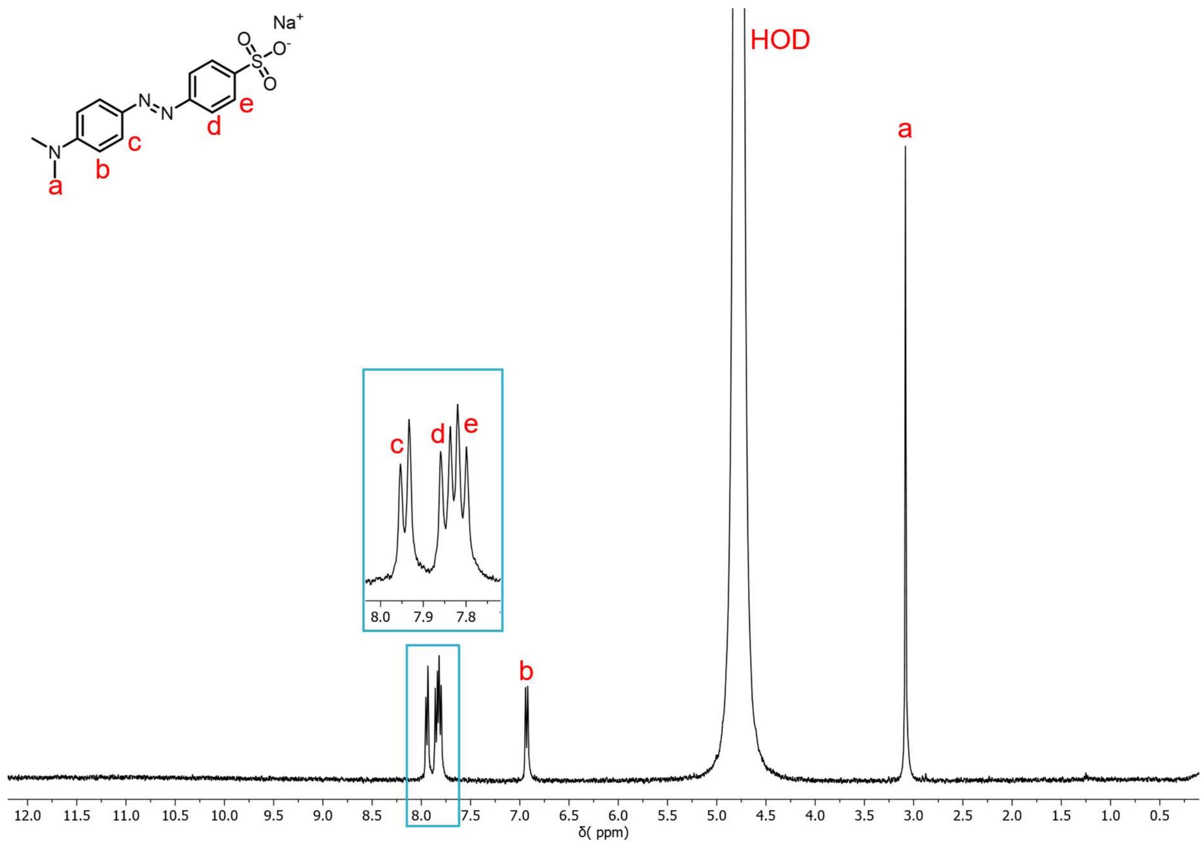

Figure S28. ^{1}H NMR spectrum (400 MHz, D_2O) of methyl orange.

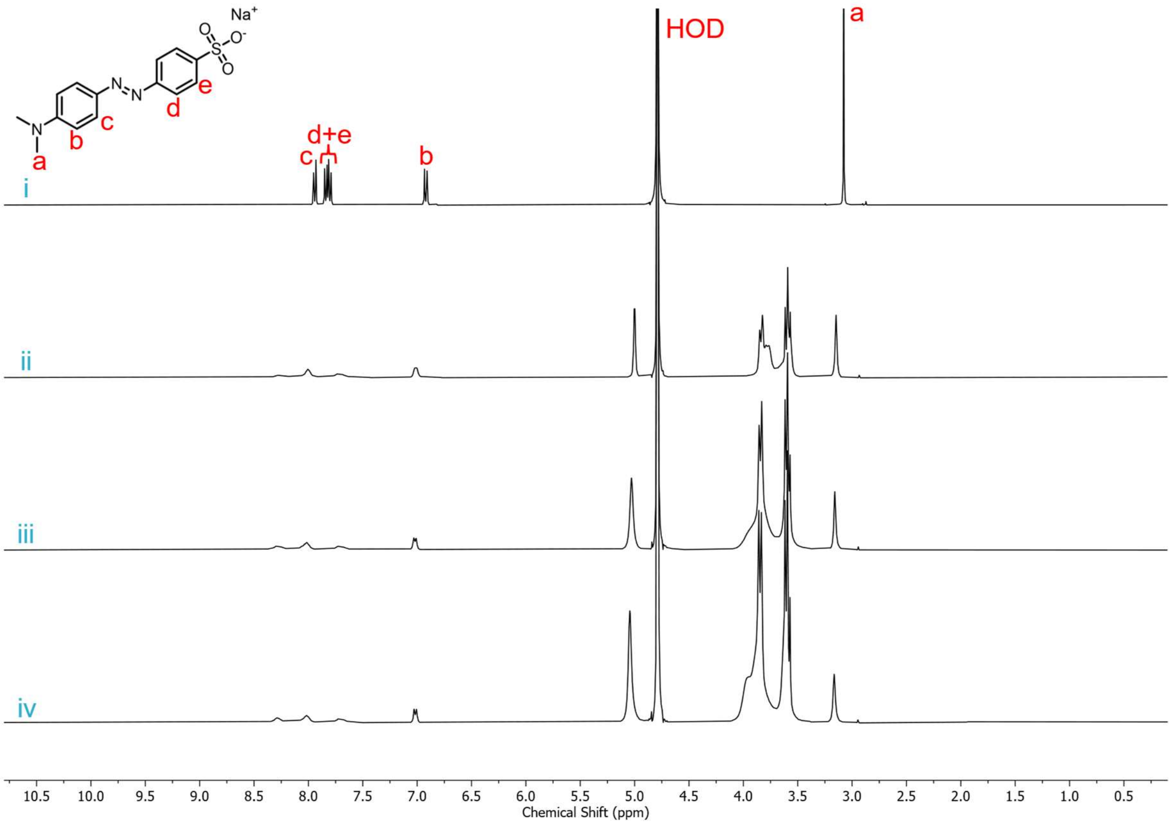

Figure S29. (From top to bottom) ^{1}H NMR spectra (400 MHz, D_2O) of i) methyl orange (2 mM), and inclusion complexes formed by mixing methyl orange and α-CD in ii) 1:1, iii) 1:2, and iv) 1:3 molar ratios.

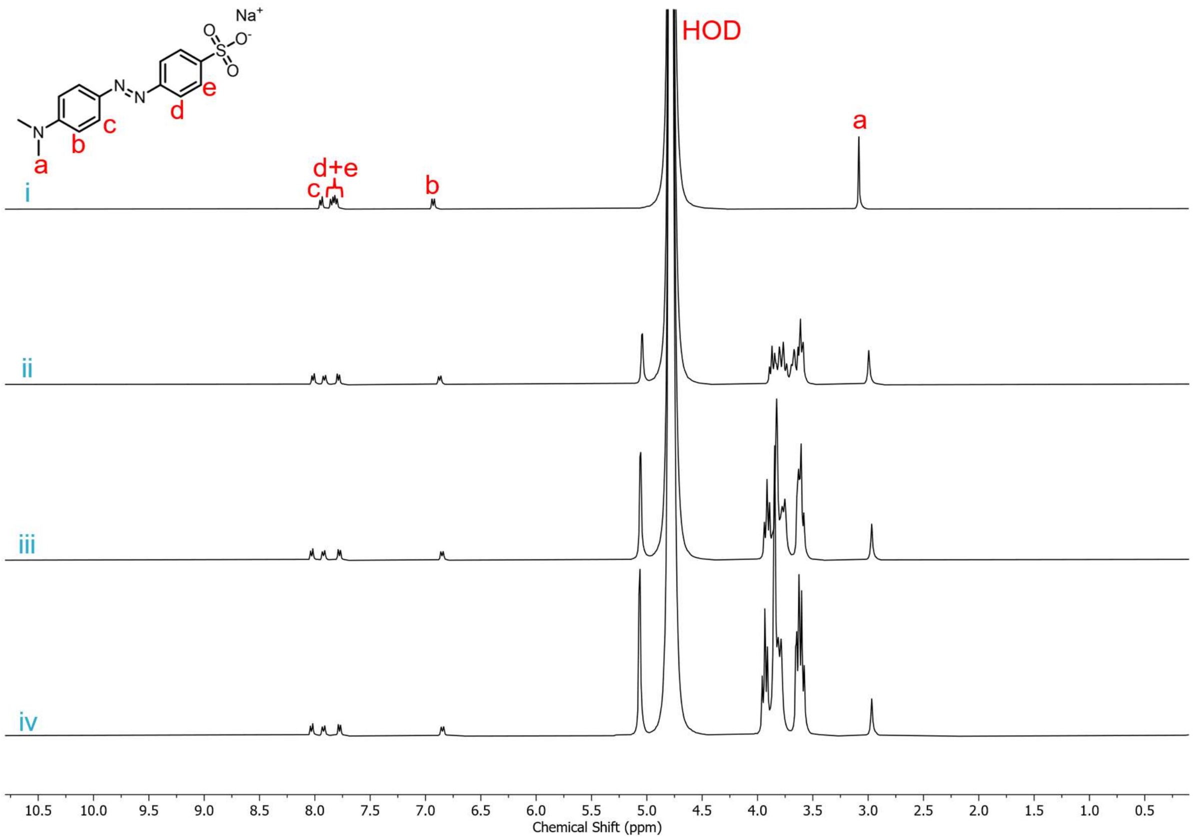

Figure S30. (From top to bottom) ^{1}H NMR spectra (400 MHz, D_2O) of i) methyl orange (2 mM), and inclusion complexes formed by mixing methyl orange and β-CD in ii) 1:1, iii) 1:2, and iv) 1:3 molar ratios.

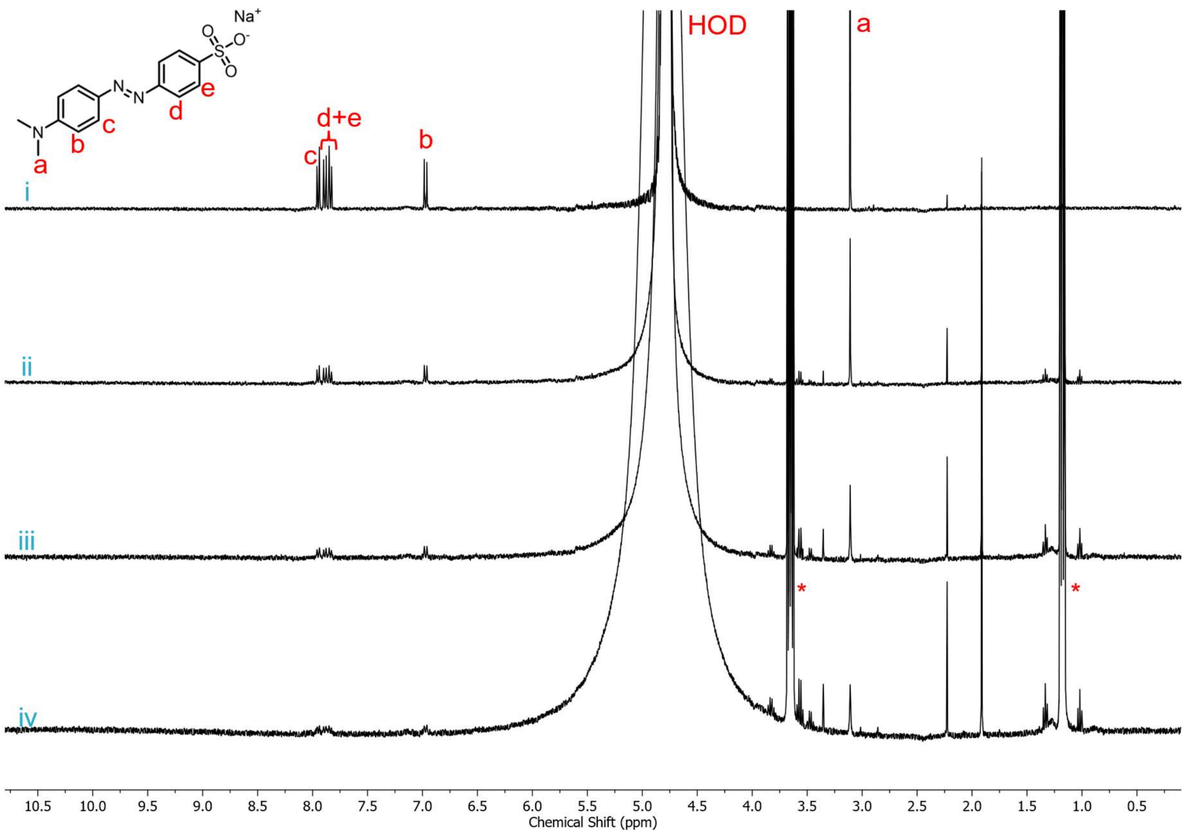

Figure S31. (From top to bottom) ^{1}H NMR spectra (400 MHz, D_2O:H_2O (1:1, v/v)) of i) methyl orange (59 μM), and inclusion complexes formed by mixing methyl orange and α-CD-BC in ii) 1:1, iii) 1:2, and iv) 1:3 molar ratios. * = residual ethanol from ultrasonication.

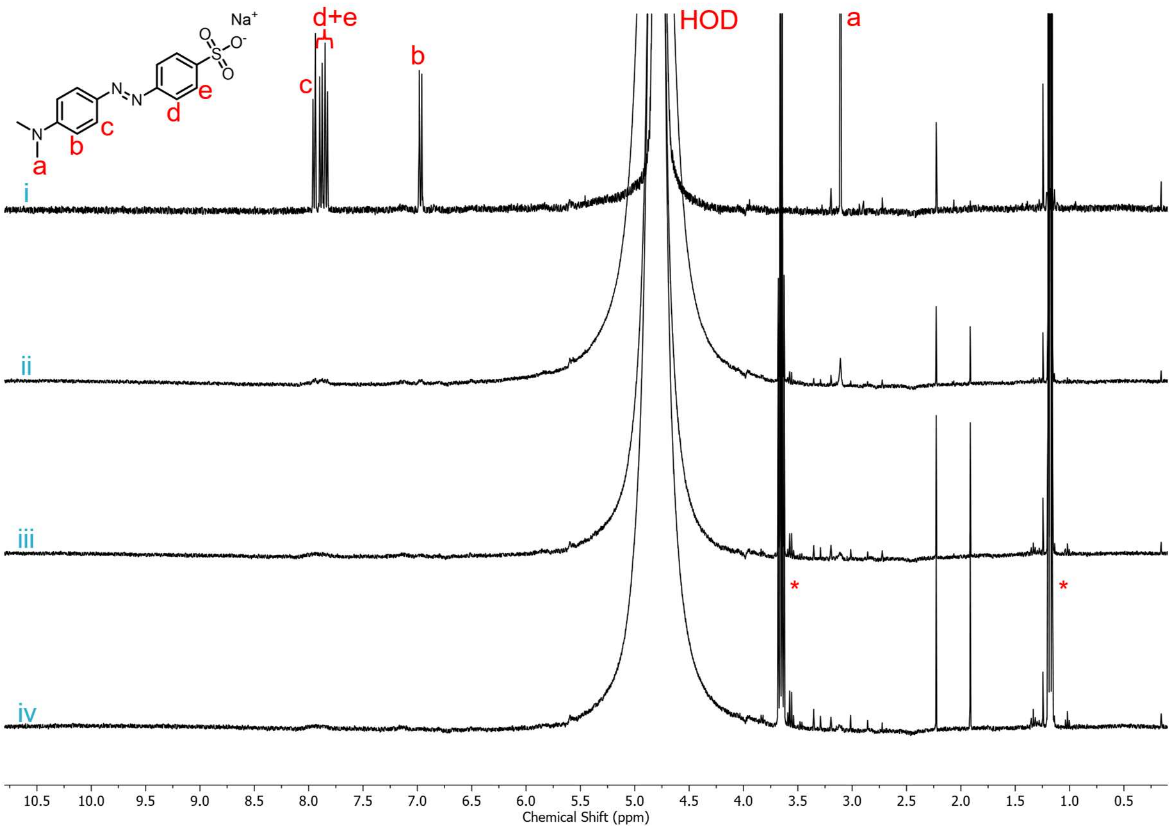

Figure S32. (From top to bottom) ^{1}H NMR spectra (400 MHz, D_2O:H_2O (1:1, v/v)) of i) methyl orange (90 µM), and inclusion complexes formed by mixing methyl orange and β-CD-BC in ii) 1:1, iii) 1:2, and iv) 1:3 molar ratios. * = reisdual ethanol from ultrasonication.

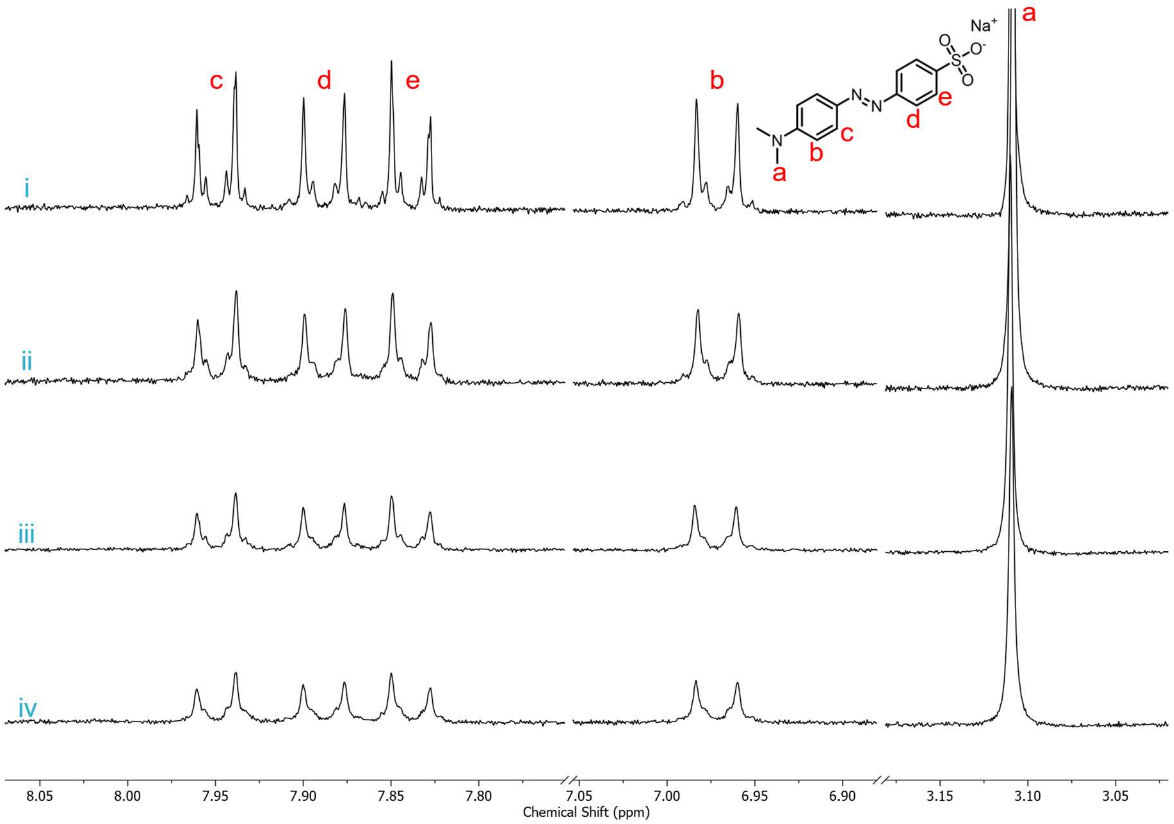

Figure S33. (From top to bottom) Partial ^{1}H NMR spectra (400 MHz, $D_2O:H_2O$ (1:1, v/v)) of i) methyl orange (59 μM), and ii-iv) mixtures of methyl orange and ungrafted BC with the concentrations of the ungrafted BC matching those of the α-CD-BC in Figure S31 ii-iv respectively.

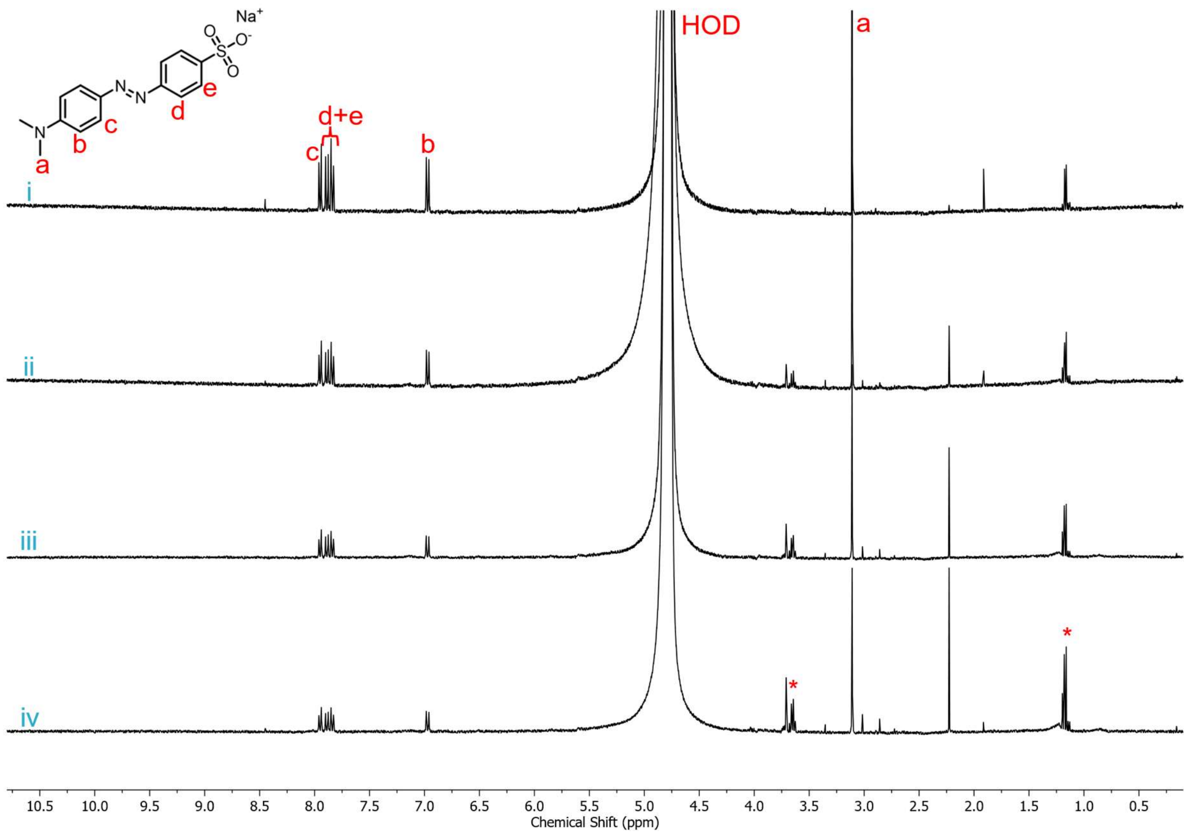

Figure S34. (From top to bottom) ^{1}H NMR spectra (400 MHz, D_2O:H_2O (1:1, v/v)) of i) methyl orange (59 μM), and ii-iv) mixtures of methyl orange and ungrafted BC with the concentrations of the ungrafted BC matching those of the α-CD-BC in Figure S31 ii-iv respectively. * = residual ethanol from ultrasonication.

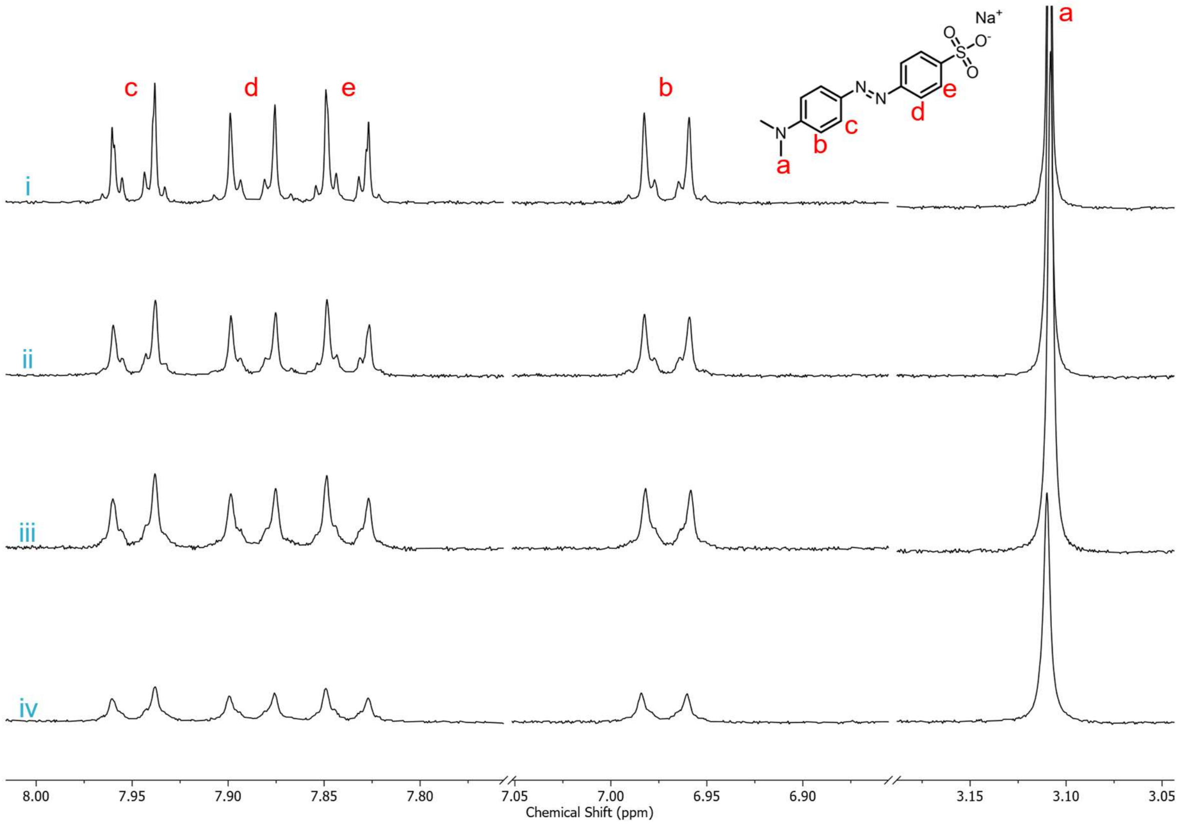

Figure S35. (From top to bottom) Partial ^1H NMR spectra (400 MHz, D_2O:H_2O (1:1, v/v)) of i) methyl orange (90 μM), and ii-iv) mixtures of methyl orange and ungrafted BC with the concentrations of the ungrafted BC matching those of the β-CD-BC in Figure S32 ii-iv respectively.

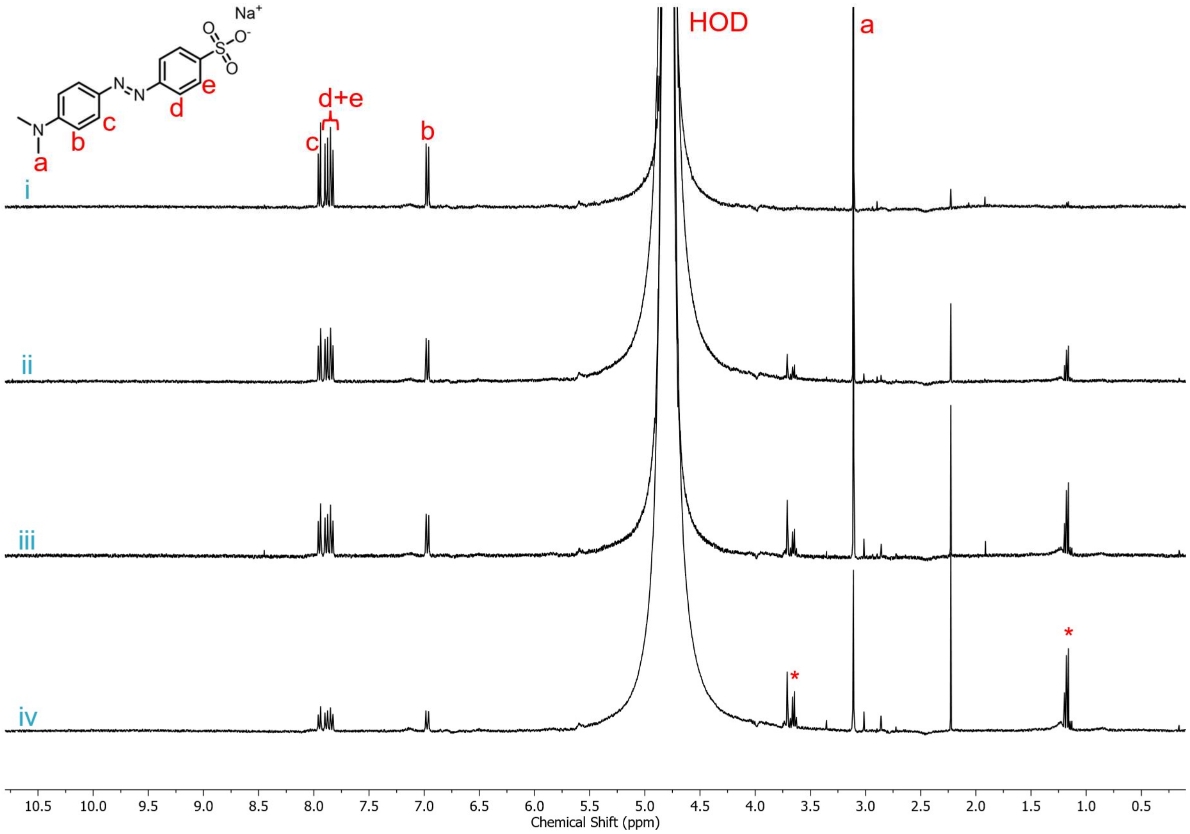

Figure S36. (From top to bottom) ^{1}H NMR spectra (400 MHz, D_2O:H_2O (1:1, v/v)) of i) methyl orange (90 μM), and ii-iv) mixtures of methyl orange and ungrafted BC with the concentrations of the ungrafted BC matching those of the β-CD-BC in **Figure S32 ii-iv** respectively. * = residual ethanol from ultrasonication.

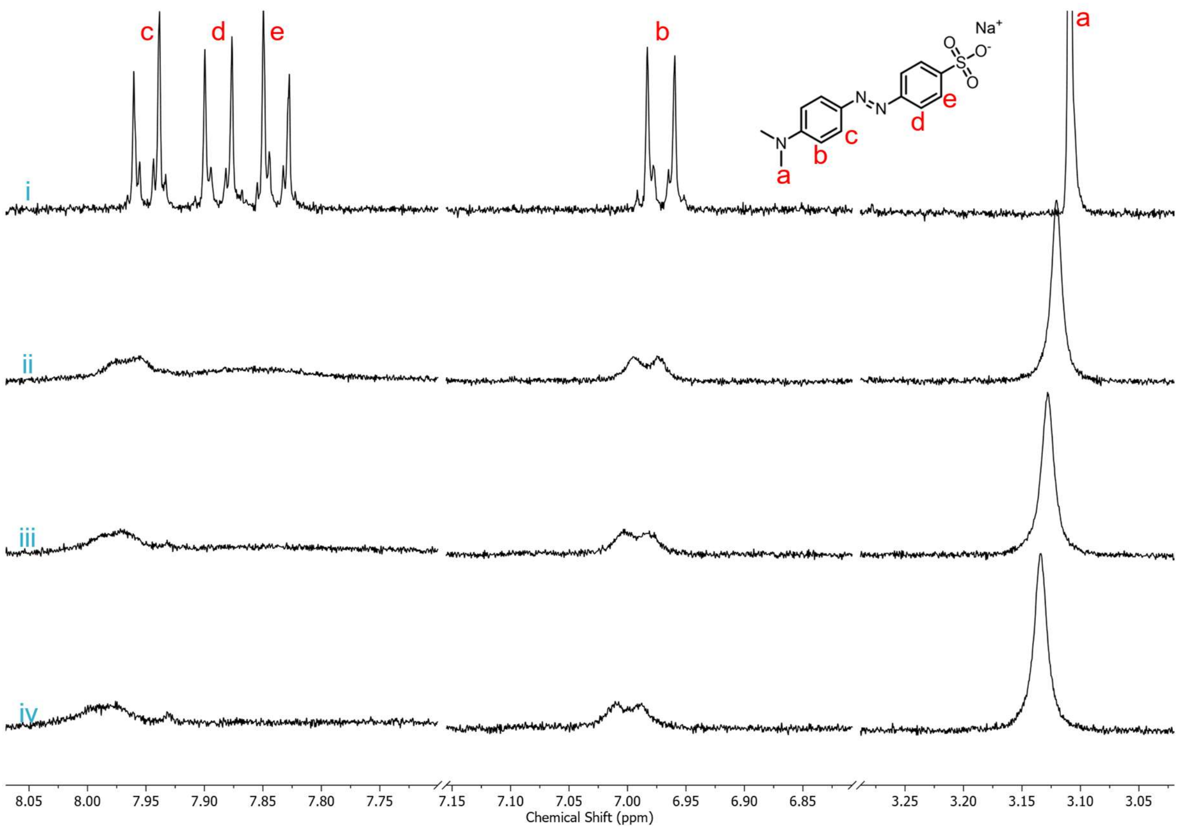

Figure S37. (From top to bottom) Partial ^{1}H NMR spectra (400 MHz, D_2O:H_2O (1:1, v/v)) of i) methyl orange (59 μM), and inclusion complexes formed by mixing methyl orange and α-CD in ii) 1:1, iii) 1:2, and iv) 1:3 molar ratios. There is no ungrafted BC in i) but ii-iv) contain ungrafted BC in the same concentration as those in **Figure S31 ii-iv** respectively.

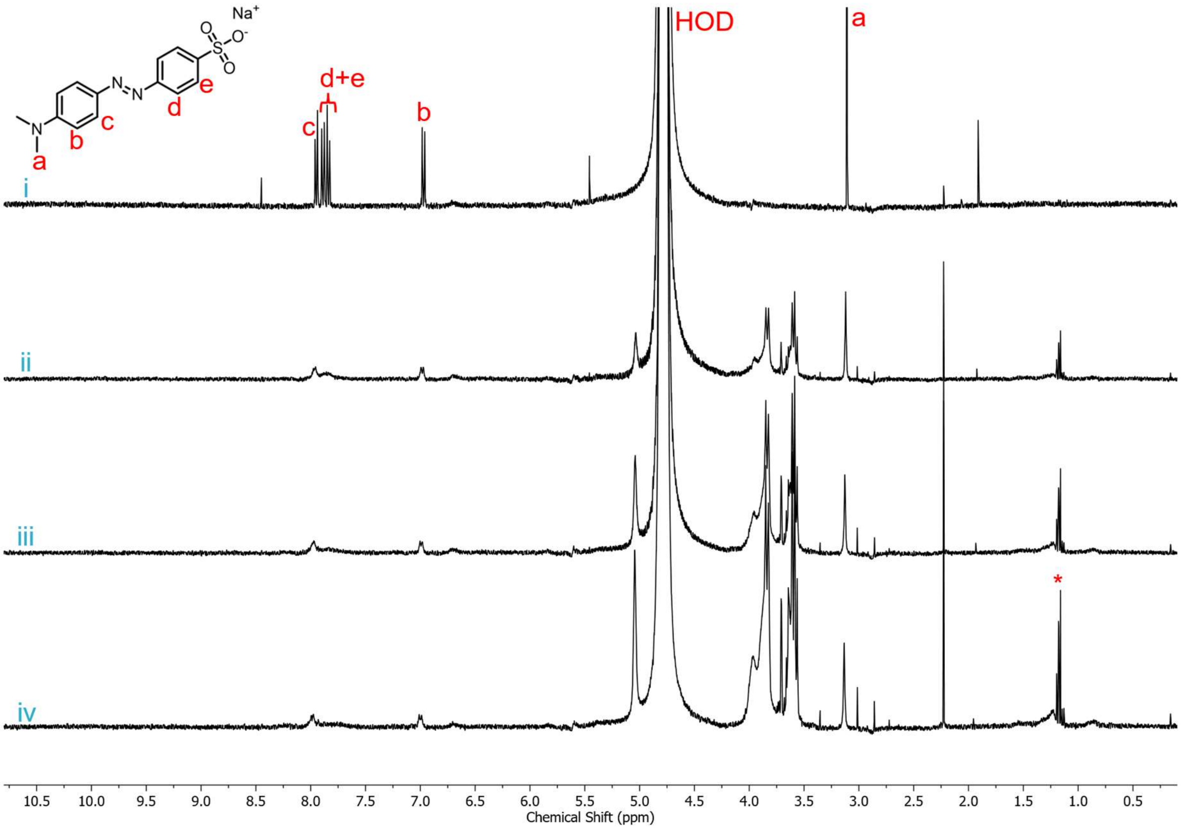

Figure S38. (From top to bottom) ^{1}H NMR spectra (400 MHz, D_2O:H_2O (1:1, v/v)) of i) methyl orange (59 μM), and inclusion complexes formed by mixing methyl orange and α-CD in ii) 1:1, iii) 1:2, and iv) 1:3 molar ratios. There is no ungrafted BC in i) but ii-iv) contain ungrafted BC in the same concentration as those in Figure S31 ii-iv respectively. * = residual ethanol from ultrasonication.

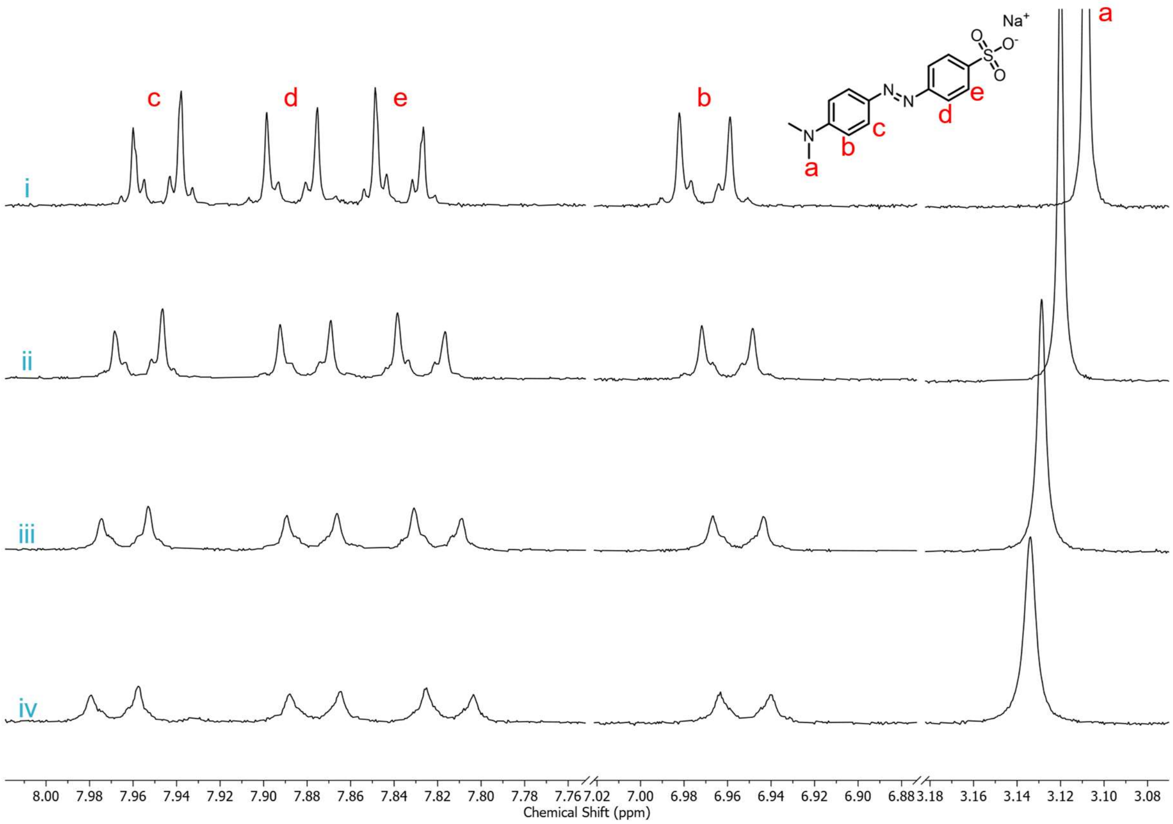

Figure S39. (From top to bottom) Partial ^{1}H NMR spectra (400 MHz, D_2O:H_2O (1:1, v/v)) of i) methyl orange (90 μM), and inclusion complexes formed by mixing methyl orange and β-CD in ii) 1:1, iii) 1:2, and iv) 1:3 molar ratios. There is no ungrafted BC in i) but ii-iv) contain ungrafted BC in the same concentration as those in Figure S32 ii-iv respectively.

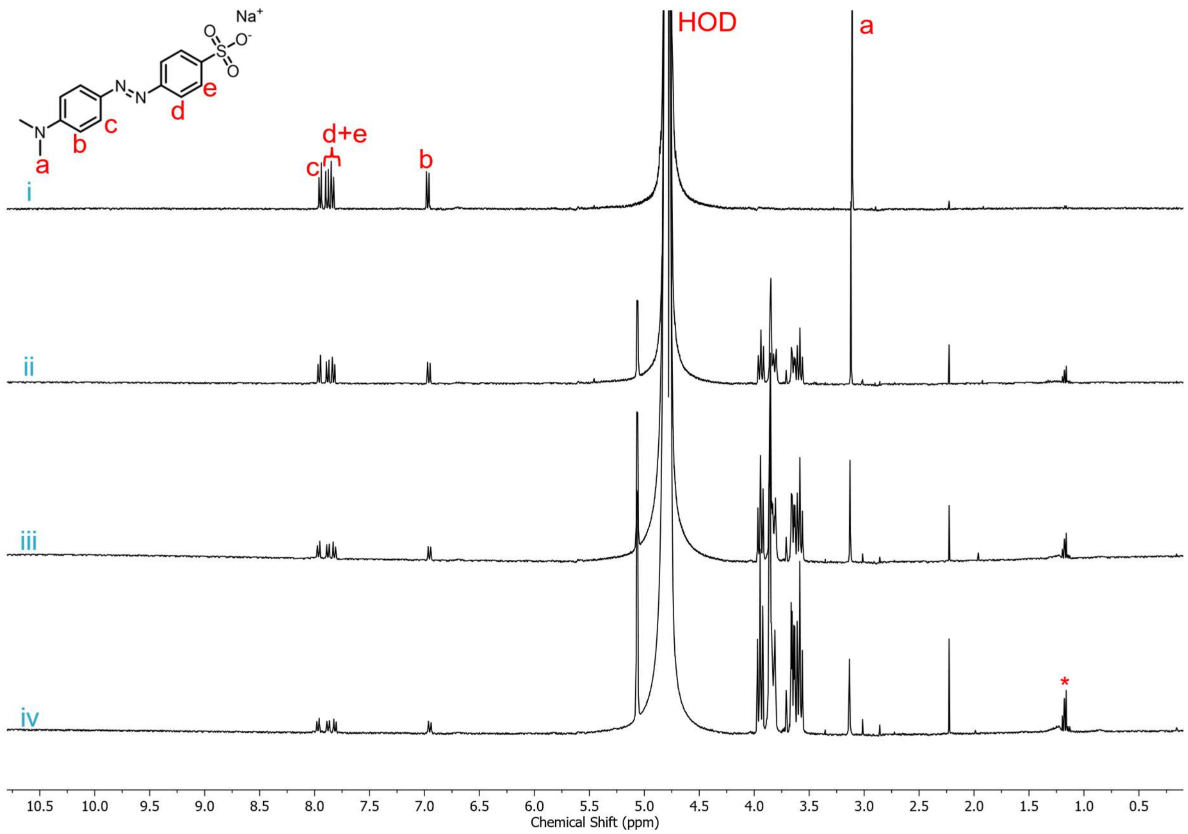

Figure S40. (From top to bottom) ^{1}H NMR spectra (400 MHz, D_2O:H_2O (1:1, v/v)) of methyl orange i) (90 μM), and inclusion complexes formed by mixing methyl orange and β-CD in ii) 1:1, iii) 1:2, and iv) 1:3 molar ratios. There is no ungrafted BC in i) but ii-iv) contain ungrafted BC in the same concentration as those in **Figure S32 ii-iv** respectively. * =residual ethanol from ultrasonication.

SUPPLEMENTARY DISCUSSION 3

In all of the ^{1}H NMR spectra containing ungrafted BC, α-CD-BC, and β-CD-BC, peaks attributed to ethanol (δ = 1.19 (t), 3.66 (q)) are visible. These result from a small amount of residual ethanol remaining on the ultrasonicator probe after cleaning. The ethanol can be removed by rotovaping, but this carries the risk of inducing aggregation of BC. As we endeavoured to avoid aggregation to improve image quality and increase the ease of AFM analysis, we opted to not apply this treatment.

As such, to verify that this residual ethanol does not affect the interaction of methyl orange with α-/β-CD, we carried out several controls; the results are given in Figures S41-S42 As shown by the ^{1}H NMR spectra, the presence of even a large amount of ethanol does not appear to result in any peak shift or attenuation for methyl orange alone and also does not appear to affect the methyl orange-CD interactions. Therefore, we are comfortable in concluding that the small amount of ethanol present in BC, α-CD-BC, and β-CD-BC has no bearing on the results of the ^{1}H NMR studies shown in this work.

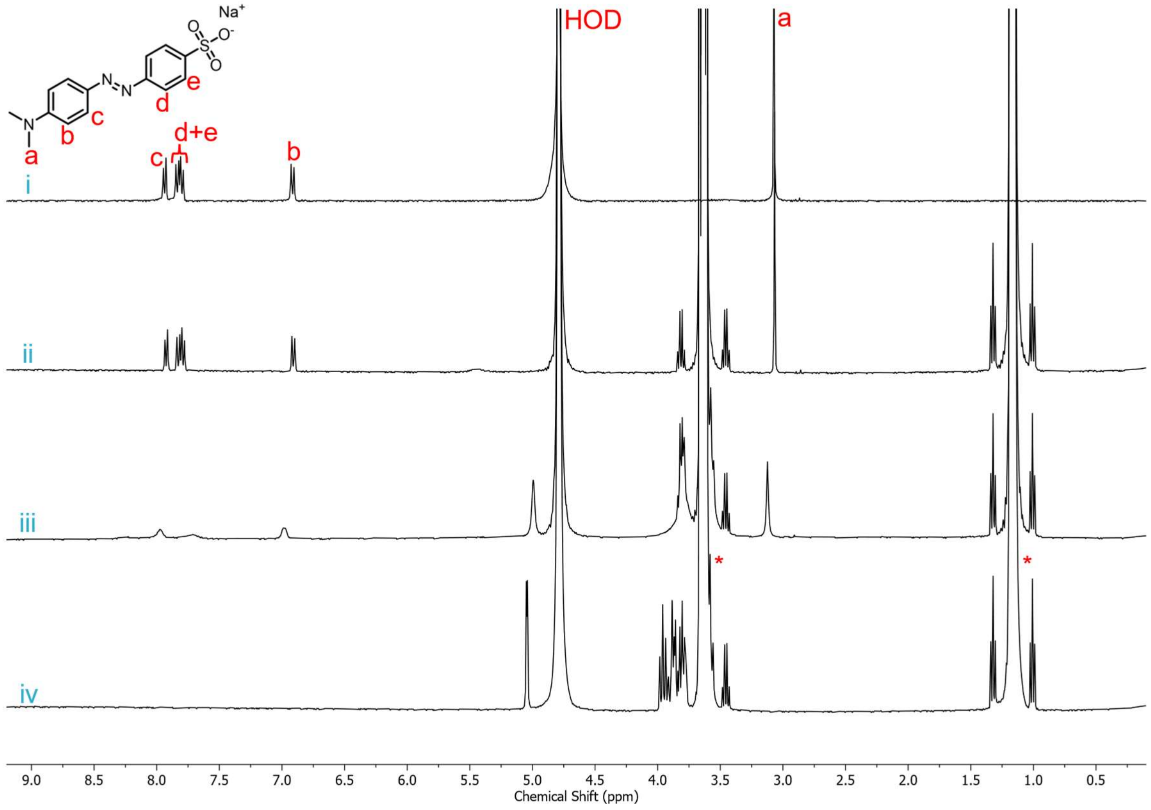

Figure S41. (From top to bottom) ^{1}H NMR spectra (400 MHz, D_2O) of i) methyl orange (2 mM), ii) methyl orange (2mM) and ethanol (0.55 M), iii) methyl orange (2 mM), α-CD (2 mM) and ethanol (0.55 M), and iv) α-CD (2 mM) and ethanol (0.55 M). * = ethanol.

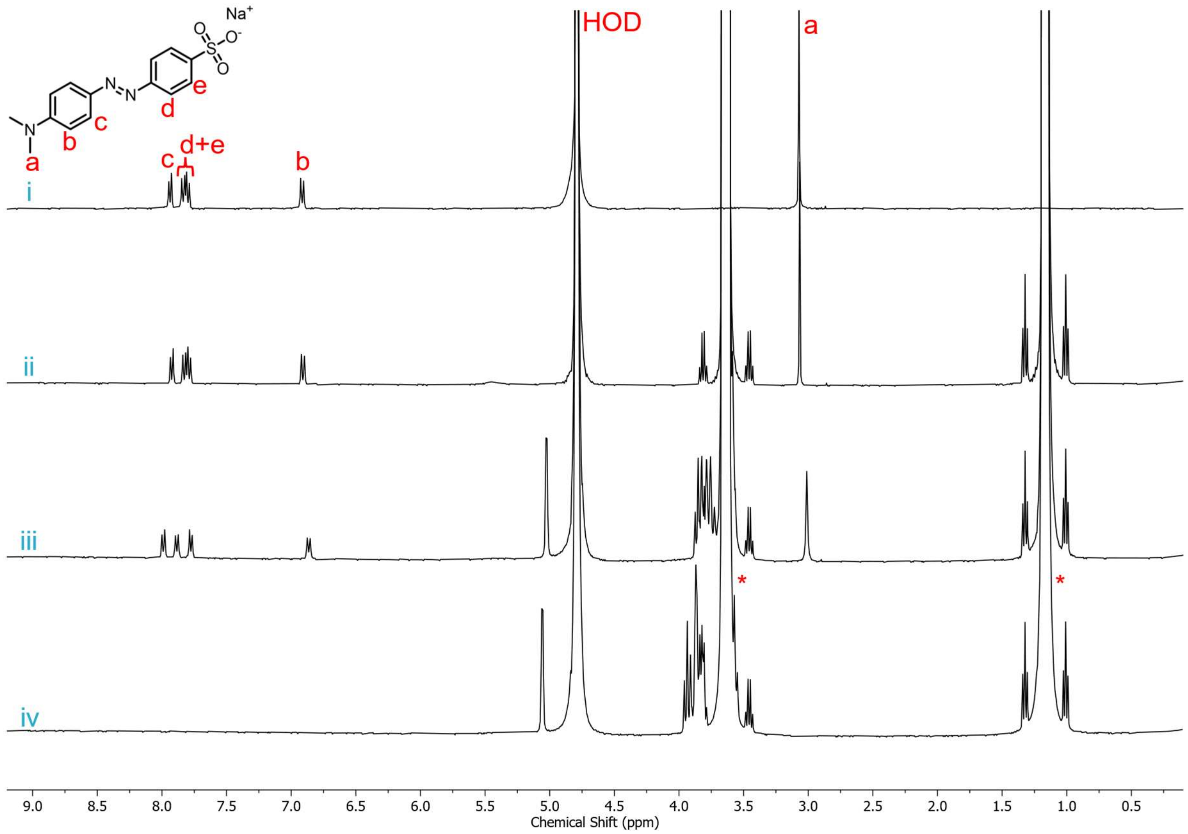

Figure S42. (From top to bottom) ^{1}H NMR spectra (400 MHz, D_2O) of i) methyl orange (2 mM), ii) methyl orange (2mM) and ethanol (0.55 M), iii) methyl orange (2 mM), β-CD (2 mM) and ethanol (0.55 M), and iv) β-CD (2 mM) and ethanol (0.55 M). * = ethanol.

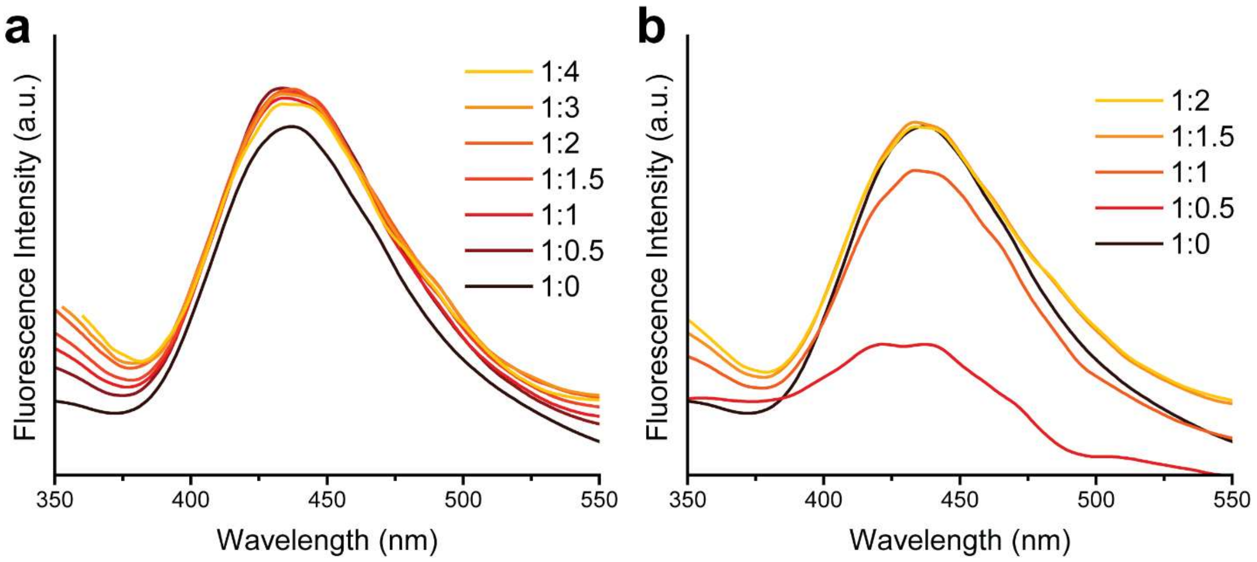

Figure S43. Fluorescence emission spectra (λ_{ex} = 310 nm) for a) methyl orange (59 µM) and α-CD-BC and b) methyl orange (90 µM) and β-CD-BC at increasing molar equivalents of α-/β-CD-BC (as shown in the inset). All spectra are corrected to control for the effect of adding suspended BC. To produce the displayed spectra, raw traces were smoothed in OriginPro 2021b using the Loess function with a span of 0.1.

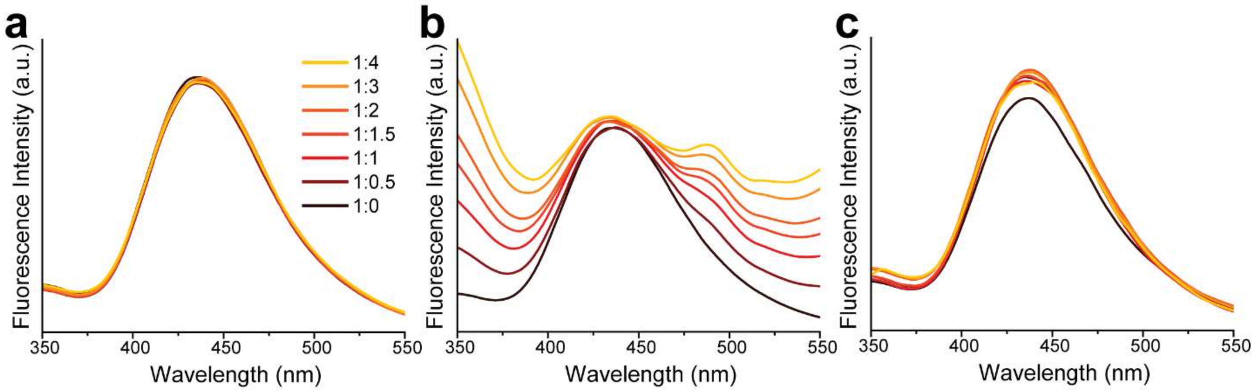

Figure S44. Fluorescence emission spectra (λ_{ex} = 310 nm) for a) methyl orange and α-CD, b) methyl orange and free BC, and c) methyl orange, free BC, and free α-CD (corrected to account for the effect of free BC). The concentration of methyl orange was kept constant for all measurements at 59 µM. In b) and c), free BC was added at the same concentration as for experiments with α-BC-CD. To produce the displayed spectra, all raw traces were smoothed in OriginPro 2021b using the Loess function with a span of 0.1.

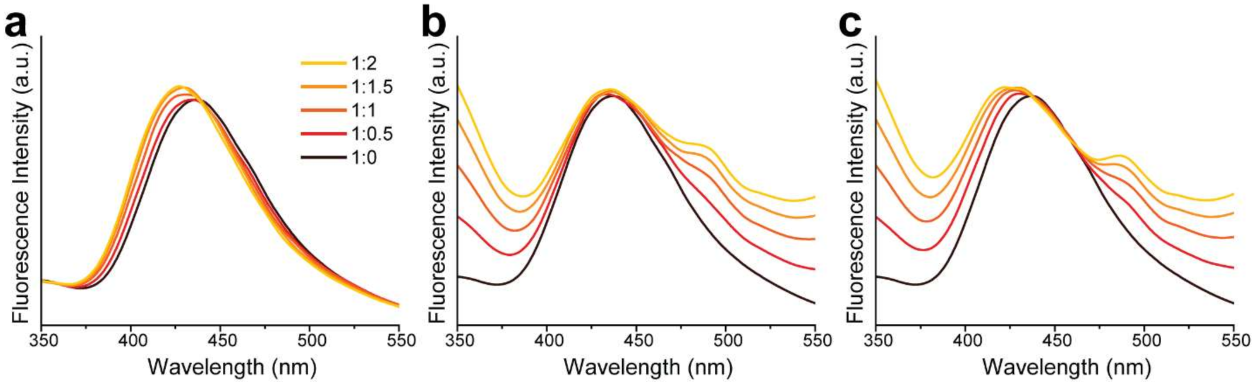

Figure S45. FL emission spectra (λ_{ex} = 310 nm) for a) methyl orange and β-CD, b) methyl orange and free BC, and c) methyl orange, free BC, and free β-CD. The concentration of methyl orange was kept constant for all measurements at 90 µM. In b) and c), free BC was added at the same concentration as for experiments with β-BC-CD. To produce the displayed spectra, all raw traces were smoothed in OriginPro 2021b using the Loess function with a span of 0.1.

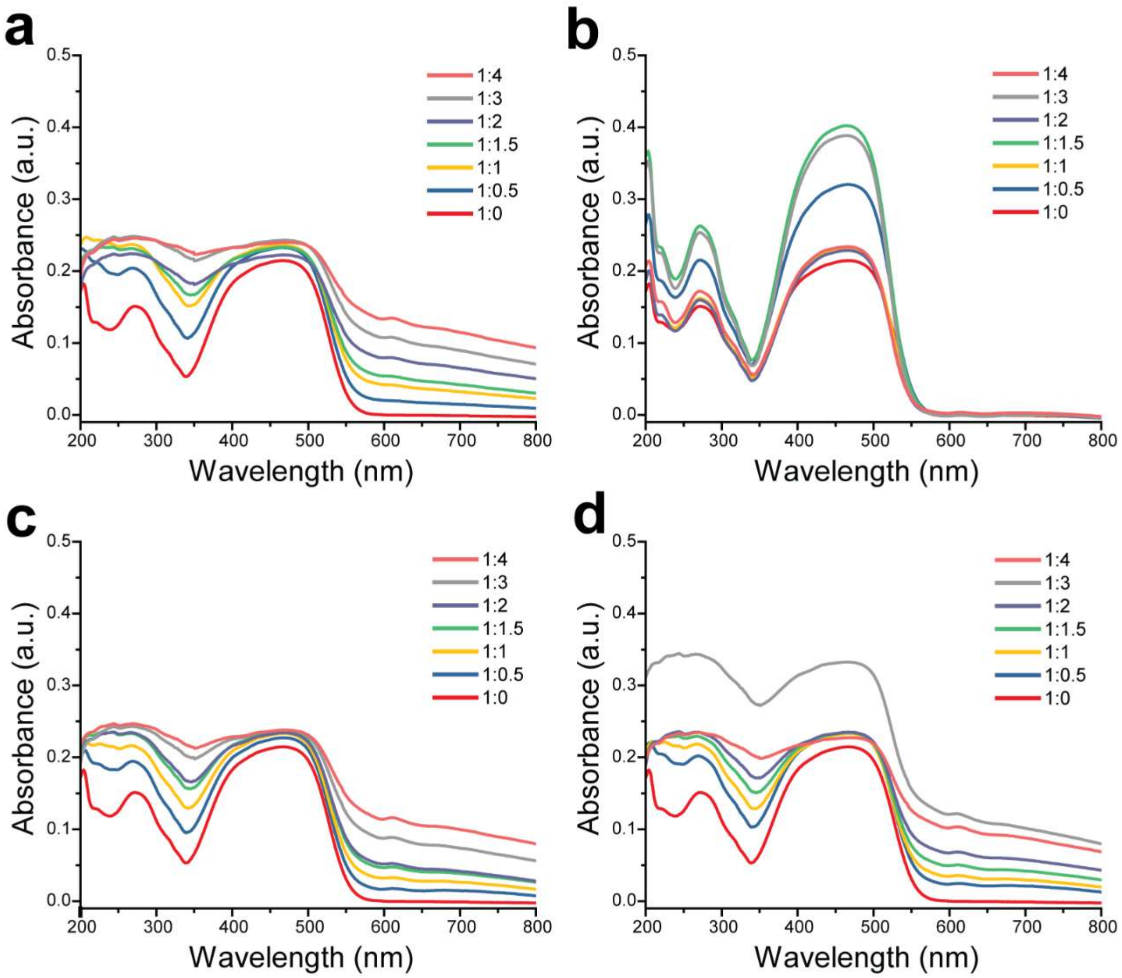

Figure S46. UV-vis spectra for methyl orange plus a) α-CD-BC, b) free α-CD, c) free BC, and d) free BC and free α-CD. The concentration of methyl orange was kept constant for all measurements at 59 µM. In b) and d), free BC was added at the same concentration as for experiments with α-BC-CD.

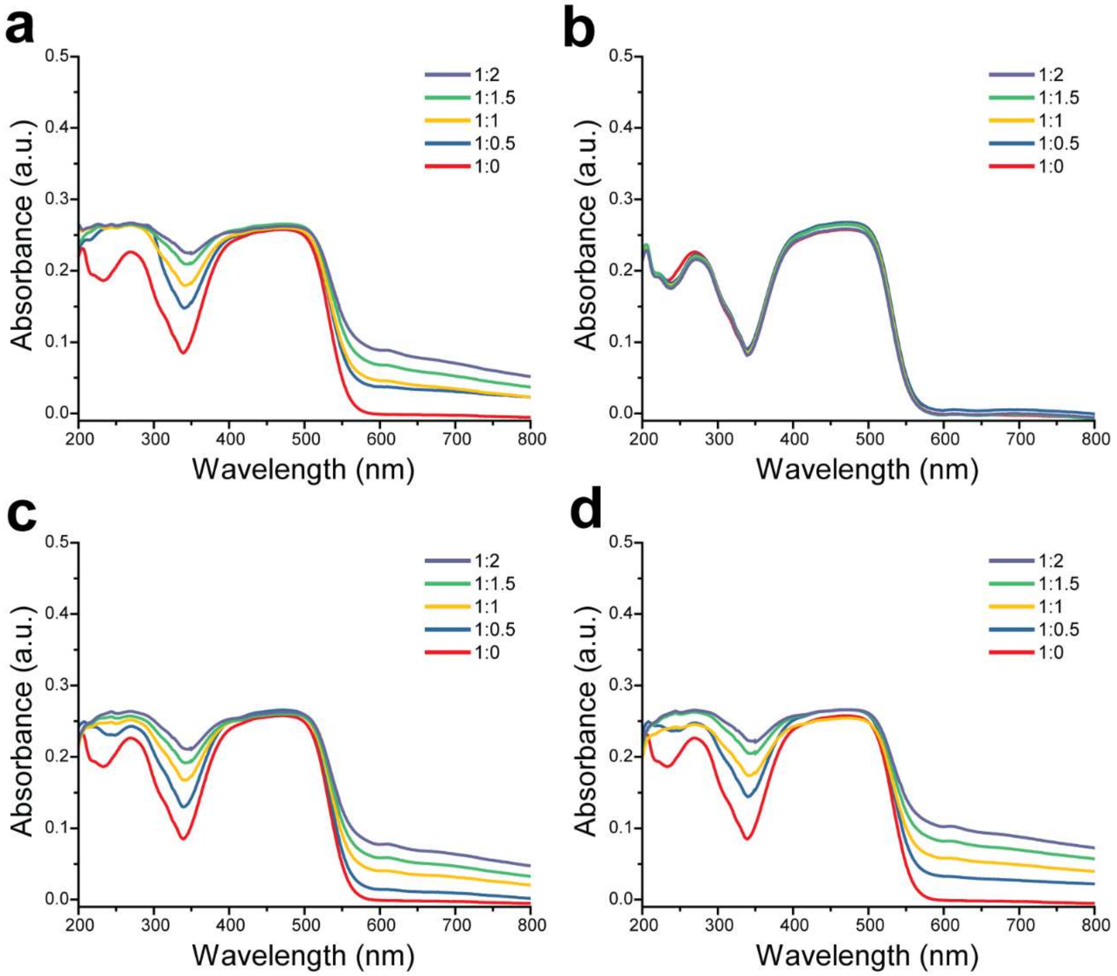

Figure S47. UV-vis spectra for methyl orange plus a) β-CD-BC, b) free β-CD, c) free BC, and d) free BC and free β-CD. The concentration of methyl orange was kept constant for all measurements at 90 μM. In b) and d), free BC was added at the same concentration as for experiments with β-BC-CD.
